# A review on lawsone-based benzo[*a*]phenazin-5-ol: synthetic approaches and reactions

**DOI:** 10.1039/d2ra02139k

**Published:** 2022-05-09

**Authors:** Abolfazl Olyaei, Mahdieh Sadeghpour

**Affiliations:** Department of Chemistry, Payame Noor University (PNU) PO BOX 19395-4697 Tehran Iran Olyaei_a@pnu.ac.ir +98-28-33374081 +98-28-33376366; Department of Chemistry, Takestan Branch, Islamic Azad University Takestan Iran mahdieh.sadeghpour@iau.ac.ir +98-28-35270165 +98-28-35270167

## Abstract

Phenazine systems are an important class of aza-polycyclic compounds that are easily found in nature and isolated as secondary metabolites primarily from *Pseudomonas*, *Streptomyces*, and a few other genera from soil or marine habitats. Moreover, various synthetic phenazine analogs are known for their pharmaceutical activities. Among various phenazines, benzo[*a*]phenazines are structural subunits in a variety of important natural products and have been given special attention due to their unique biological properties in various fields. In this review article, we highlight the synthesis of benzo[*a*]phenazin-5-ol derivatives from lawsone and benzene-1,2-diamines and their applications for the construction of a variety of five and six membered fused heterocycles such as pyranophenazines, spiropyranophenazines, pyridophenazines, furophenazines, benzochromenophenazines and oxazinophenazines during the period of 1995 to 2021.

## Abstract 2

Phenazine systems are an important class of aza-polycyclic compounds that are easily found in nature and isolated as secondary metabolites primarily from *Pseudomonas*, *Streptomyces*, and a few other genera from soil or marine habitats.

## Graphical abstract

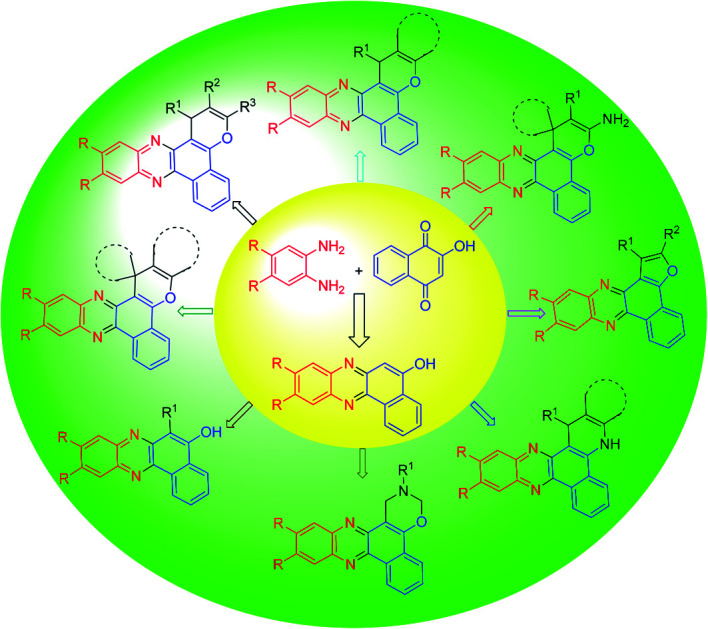

## 1 Introduction

Phenazine systems are an important class of aza-polycyclic compounds that are easily found in nature. More than 6000 phenazine-containing compounds have been recognized and reported during the past century, including natural phenazines and compounds synthesized based on the phenazine skeleton. Phenazine natural products are isolated as secondary metabolites primarily from *Pseudomonas*, *Streptomyces*, and a few other genera from soil or marine habitats. The biological properties of this class of natural products have been reviewed. In 1986, Turner and Messenger described the natural occurrence and some properties of phenazines, biosynthesis, secondary metabolism and the physiological significance of phenazine production in a review article.^1^ After that, the role of phenazine pigments as antibiotics and virulence factors was reviewed by Kerr in 2000.^2^ Next, Laursen and Nielsen described exclusively with a representative selection of biologically significant phenazines, their natural occurrence, their biosynthesis, the design and synthesis of analogues, and their biological function and possible mode of action in 2004.^3^ The progress in the isolation of new phenazine natural products, new insights in their biological function, and particularly the now almost completely understood biosynthesis has been briefly reviewed recently.^4^

Moreover, various synthetic phenazine analogs are known for their pharmaceutical activities such as antifungal, antimalarial, antileishmanial, antihepatitis C viral replication, trypanocidal, inhibition of the cyclooxygenase, interactions of serum albumins, antimicrobial, anti-inflammatory, antitumor, as well as insecticidal activity.^5–16^ Fluorescent phenazine derivatives both natural and synthetic, are also of interest because of their rapidly expanding applications as emitters for electroluminescence devices,^17^ organic semiconductors,^18^ photo-sensitizers in photodynamic therapy,^19^ promoter for proliferation,^20^ dye-sensitized solar cells (DSSCs),^21^ electrochemical, and biosensors sensitive to H_2_O_2_, glucose, and lactose.^22–24^ The synthetic routes for the synthesis of this scaffold have been reviewed. The general approaches for synthesis of phenazines include the Wohl–Aue method, Beirut method, condensation of 1,2-diaminobenzenes with 2C-units, reductive cyclization of diphenylamines, oxidative cyclization of 1,2-diaminobenzene/diphenylamines, Pd-catalyzed N-arylation, multicomponent approaches and benzyne intermediate has been reviewed by Chaudhary and Khurana in 2018.^25^ Recently, Elhady and co-workers reviewed the synthesis of phenazines, either chemically or biologically and, also the different reactions of them and some of their biological importance, and their applications in the development of electrochemical sensors, biosensors and dye-sensitized solar cells (DSSCs).^26^ Among various phenazine derivatives, benzo[*a*]phenazines that have a napthoquinone and phenazin backbone in their structures are structural subunits in a variety of important natural products and has been given special attention due to their unique biological properties in various fields such as dual inhibitors of topoisomerase I and II and are useful as antitumor agents.^27^

2-Hydroxy-1,4-naphthoquinone, or lawsone, or hennotannic acid, is one of the simplest naturally occurring naphthoquinones which can be obtained from the extract of dried powdered leaves of henna. Lawsone as a red-orange pigment is traditionally used for coloring hair and dying nails and skin, silk, wool and leather. It is reveals a long list of applications, including skin protection from ultraviolet radiation, corrosion inhibition for steel, antiaging additive to vulcanized natural rubber, oxidation of chlorinated compounds and sensitive colorimetric and electrochemical sensor for anions. Moreover, it has been used as the starting material for the synthesis of a variety of biologically active compounds and materials with interesting properties.^28–31^ This review highlights the synthesis of benzo[*a*]phenazin-5-ol derivatives from lawsone and benzene-1,2-diamines and their applications for the construction of a variety of five and six membered fused heterocycles.

## 2 Synthesis of benzo[*a*]phenazin-5-ols

**Scheme 1:**
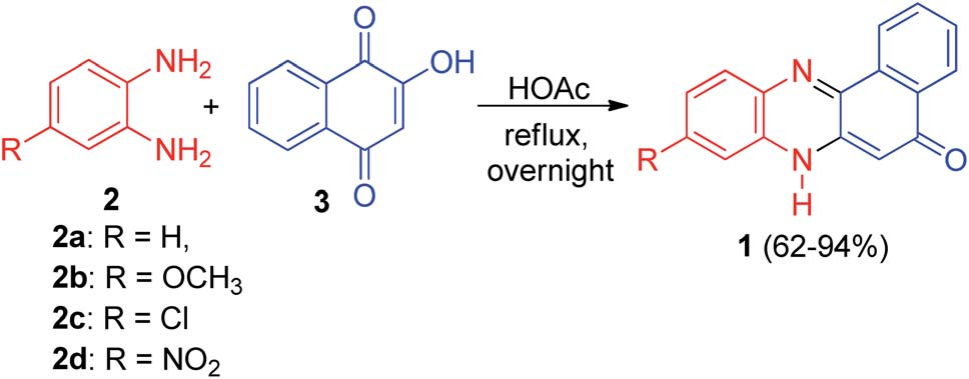
Synthesis of 5,7-dihydrobenzo[*a*]phenazin-5-one derivatives 1.

The first synthesis of 5,7-dihydrobenzo[*a*]phenazin-5-one derivatives 1 in good yields (62–94%) was reported by Rehberg and Rutherford in 1995. The general synthesis involved condensation of aromatic 1,2-diamines 2 with 2-hydroxy-1,4-naphthoquinone (lawsone) (3) in the presence of acetic acid under reflux conditions for overnight (Scheme 1).^32^

**Scheme 2:**
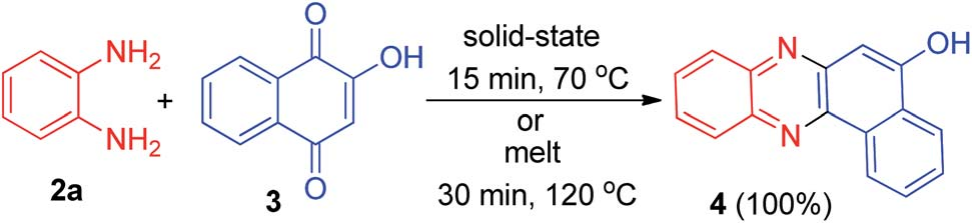
Quantitative synthesis of benzo[*a*]phenazin-5-ol (4).

In 2002, Kaupp and Naimi-Jamal reported synthesis of benzo[*a*]phenazin-5-ol (4) in 100% yield by the one-pot condensation reaction of 3 and *o*-phenylenediamine (2a) in solid-state 1 : 1 runs in 15 min at 70 °C. If the same reaction was performed as a melt at 120 °C for 30 min, a 100% yield was also obtained (Scheme 2).^33^

**Scheme 3:**
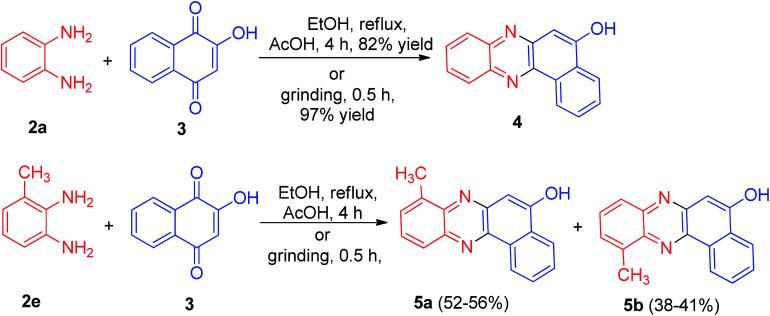
Synthesis of tetracyclic phenazine derivatives 4 and 5.

In 2013, Jain and co-workers described synthesis of tetracyclic phenazine derivatives 4 and 5 in 38–97% yields. The reaction of 3 with 2a in refluxing EtOH in the presence of AcOH as catalyst for 4 h afforded 4 while refluxing with 2,3-diaminotoluene (2e) gave the mixture of the regioisomers 5a–b. These reactions were also carried out under mortar-pestle grinding technique, where the reaction was complete in lesser time and in enhanced yield (Scheme 3).^34^

**Scheme 4:**
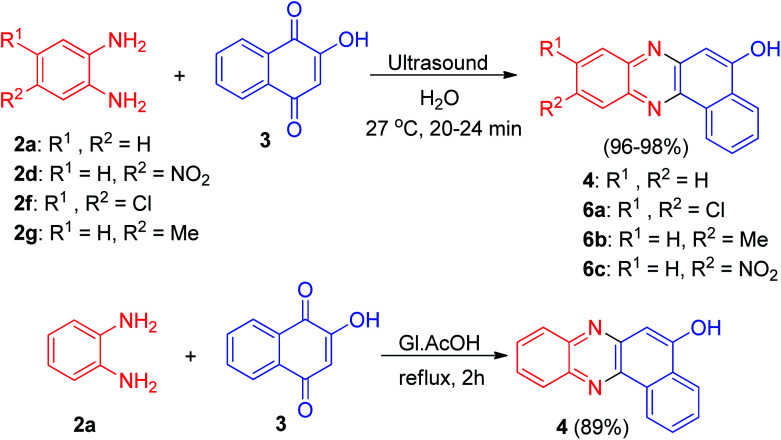
One-pot synthesis of benzophenazines 4 and 6a–c.

In 2014, Sekar and co-workers developed synthesis of benzophenazines 4 and 6a–c in 96–98% yields from lawsone (3) and 1,2-benzenediamines 2 under ultrasound irradiation in an aqueous media at 27 °C for 20–24 min. Also, the reaction of 3 and 2a was carried out by conventional method by refluxing in glacial acetic-acid for 2 h afforded the desired product 4 in 89% yield (Scheme 4).^35^

### 2.1 Synthesis of benzopyranophenazines

**Scheme 5:**
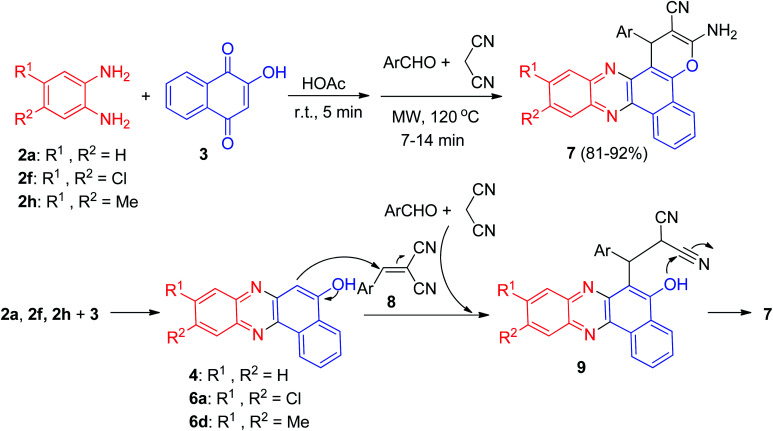
One-pot two-step synthesis of benzo[*a*]pyrano[2,3-*c*]phenazine derivatives 7.

In 2011, Jiang and co-workers synthesized highly functionalized benzo[*a*]pyrano[2,3-*c*]phenazine derivatives 7 in 81–92% yields *via* one-pot two-step reactions of 3, diamines, aldehydes and malononitrile in AcOH under microwave irradiation at 120 °C for 7–14 min. The formation of 7 is expected to proceed *via* initial condensation of 3 and diamine to afford benzo[*a*]phenazin-5-ol derivatives 4, 6a and 6d, which undergoes *in situ* Michael addition with 2-benzylidenemalononitrile 8, formed from condensation of aldehydes with malononitrile, to yield intermediate 9, which is then cyclized to afford the product 7 (Scheme 5).^36^

**Scheme 6:**
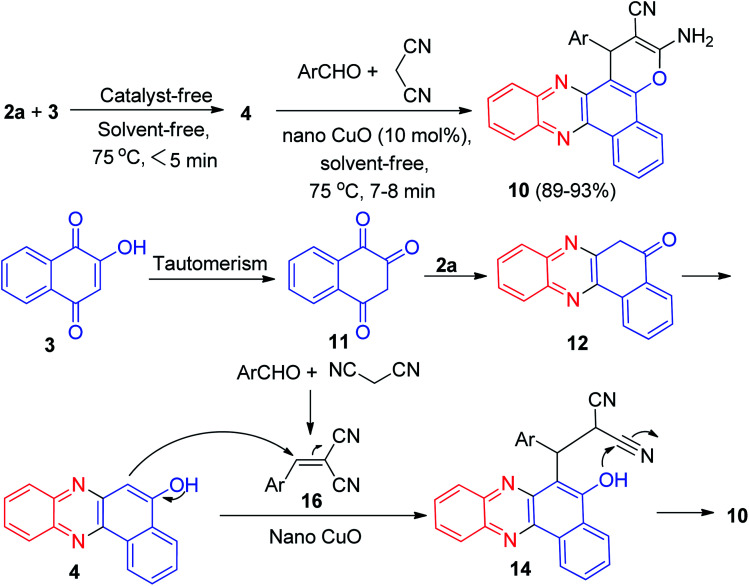
Nano CuO catalyzed synthesis of 3-amino-2-cyano-1-aryl-1*H*-benzo[*a*]pyrano[2,3-*c*]phenazines 10.

Next, an efficient one-pot two step quantitative procedure for the preparation of functionalized benzo[*a*]pyrano[2,3-*c*]phenazine derivatives 10 was reported from four-component reaction of 3, 2a, aromatic aldehydes, and malononitrile in the presence of nano CuO (10 mol%) as the catalyst at 75 °C under solvent-free conditions. The mechanism for the formation of the products has been suggested in Scheme 6. First, 3 tautomerizes to intermediate 11. The initial condensation of 11 with 2a affords 6*H*-benzo[*a*]phenazin-5-one (12), which in tautomerism equilibrium causes to prepare 4. In addition, standard Knoevenagel condensation of malononitrile and aryl aldehydes in the presence of nano CuO as the catalyst afforded benzylidenemalononitrile (13). The Michael addition of 4 with 13 formed intermediate 14, which in subsequent cyclization and tautomerism gave the corresponding product 10. The wide ranges of substituted and structurally diverse aldehydes afforded the corresponding products in high to excellent yields (89–93%).^37^

**Scheme 7:**
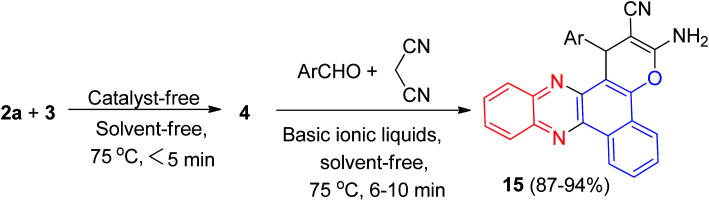
Basic ionic liquid catalyzed synthesis of 3-amino-2-cyano-1-aryl-1*H*-benzo[*a*]pyrano[2,3-*c*]phenazines 15.

After that, an efficient one-pot two-step quantitative procedure for the preparation of functionalized benzo[*a*]pyrano[2,3-*c*]phenazine derivatives 15 in 87–94% yields reported from four-component reaction of 3, 2a, aromatic aldehydes, and malononitrile in the presence of basic ionic liquids such as 1-butyl-3-methylimidazolium hydroxide, 3-hydroxypropanaminium acetate, pyrrolidinium formate, pyrrolidinium acetate, 1,8-diazabicyclo[5.4.0]-undec-7-en-8-ium acetate, and piperidinium formate as the catalysts under solvent-free conditions in 75 °C for 6–10 min (Scheme 7). The mechanism of the reaction is similar to the proposed mechanism in Scheme 6.^38^

**Scheme 8:**
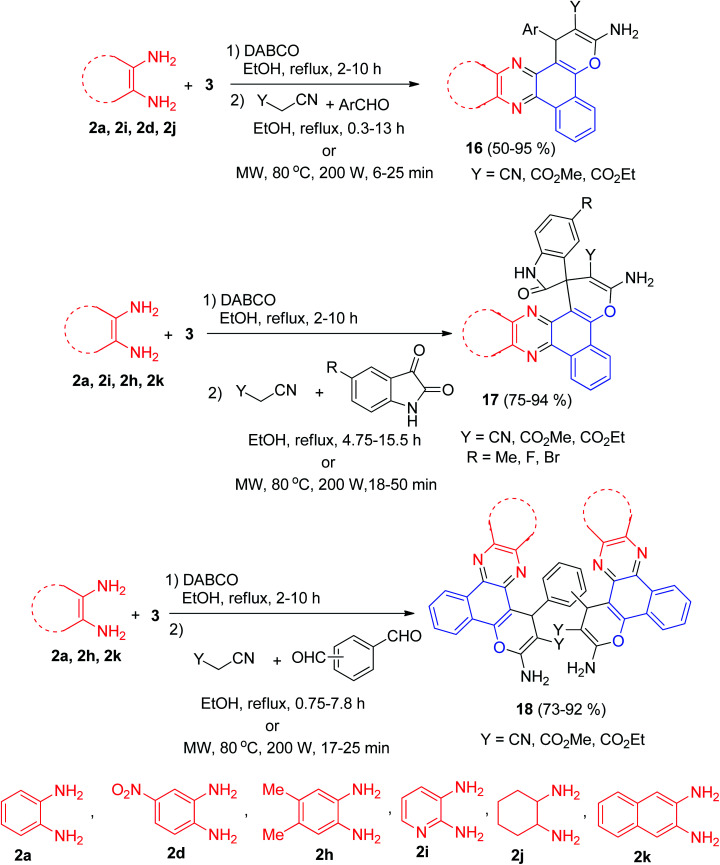
Synthesis of pyrano[3,2-*a*]phenazine derivatives 16–18.

Later, DABCO as an efficient and reusable solid base catalyst was used for the one-pot, two-step, four-component synthesis of benzo[*c*]pyrano[3,2-*a*]phenazines 16 in 50–95% yields, oxospirobenzo[*c*]pyrano[3,2-]phenazines 17 in 75–94% yields and bis-benzo[*c*]pyrano[3,2-*a*]phenazines 18 in 73–92% yields by the condensation reaction of 3, 1,2-diamines, carbonyl compounds and alkylmalonates under conventional heating (EtOH, under reflux conditions) as well as microwave irradiation (80 °C, 200 W) (Scheme 8).^39^

**Scheme 9:**
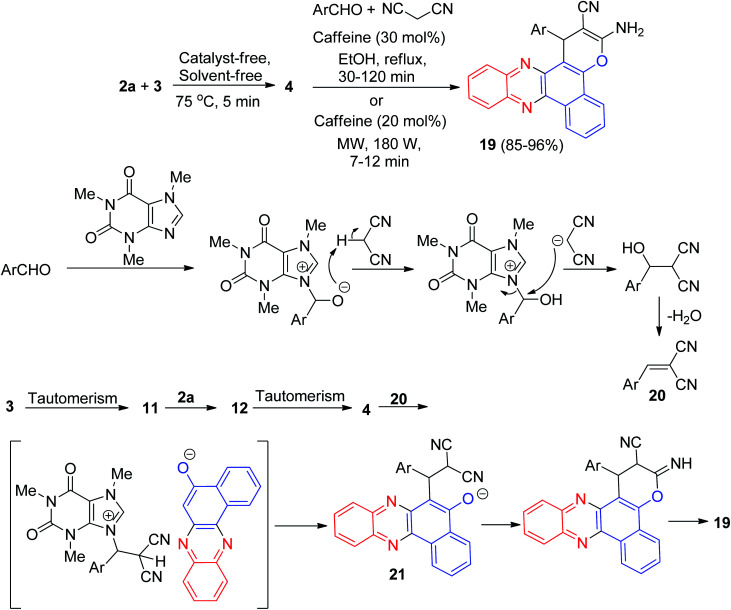
Synthesis of 3-amino-2-cyano-1-aryl-1*H*-benzo[*a*]pyrano[2,3-*c*]phenazines derivatives 19.

In 2016, a one-pot, two-step procedure was used to synthesize functionalized benzo[*a*]pyrano[2,3-*c*]phenazine derivatives 19 in 85–96% yields from a four-component condensation reaction of 3, 2a, aromatic aldehydes, and malononitrile in the presence of 1,3,7-trimethylpurine-2,6-dione (caffeine) as an expedient and reusable solid base catalyst under conventional heating and microwave irradiation. The mechanism for the formation of the products has been proposed in Scheme 9. On the basis of this mechanism, at first, 3 tautomerizes to intermediate 11. The primary condensation of 11 with 2a obtains 12, which in tautomerism equilibrium reasons to prepare 4. On this mechanism, caffeine is an impressive catalyst to form the olefin 20, which easily prepares *in situ* from Knoevenagel condensation of aldehyde with malononitrile. The Michael addition of 4 with 20 in the presence of caffeine finally give intermediate 21, which then causes the intermolecular ring to be formed after a tautomeric proton shift to produce 19.^40^

**Scheme 10:**
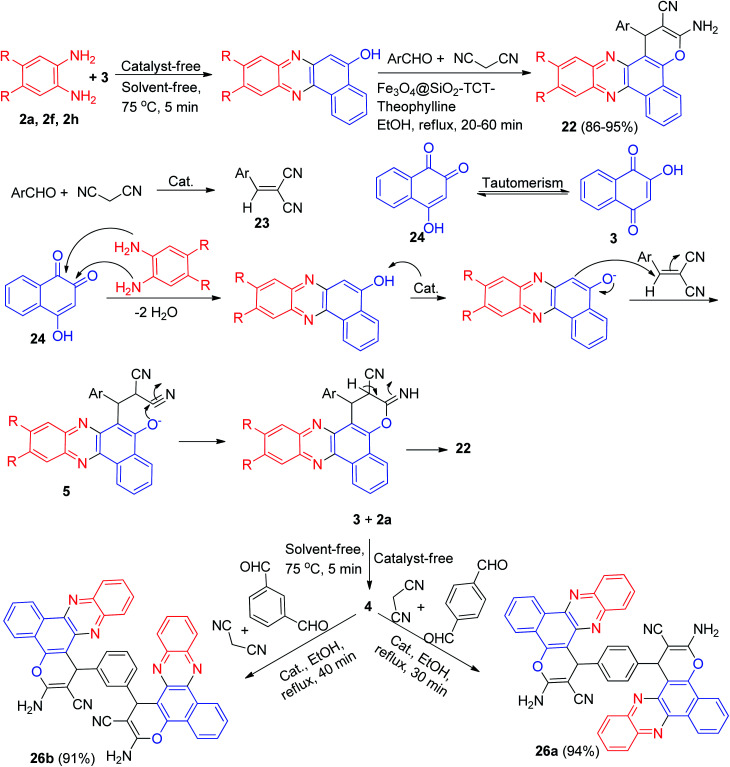
Synthesis of 3-amino-2-cyano-1-aryl-1*H*-benzo[*a*]pyrano[2,3-*c*]phenazine derivatives 22 and 26a–b.

Next, Firouzabadi and his group described theophylline immobilized on superparamagnetic Fe_3_O_4_@SiO_2_ nanoparticles catalyzed synthesis of poly-substituted benzo[*a*]pyrano-[2,3-*c*]phenazine derivatives 22 in 86–95% yields from four-component reaction of 3, diamines, aldehydes, and malononitrile in refluxing EtOH within 20–60 min. The proposed mechanism has been shown in Scheme 10. The intermediate 23 was constructed upon initial condensation of malononitrile with aldehyde in the presence of the catalyst. The condensation reaction of 3's tautomer 24 with phenylenediamine derivatives presents the corresponding benzo[*a*]phenazin-5-ol which reacts with intermediate 23 to form 25. Intramolecular cyclization of 25 affords the desired compound 22. Moreover, the synthesis of bis 3-amino-1*H*-benzo[*c*]pyrano[3,2-*a*]phenazine derivatives 26a–b in 91–94% yields has been reported *via* the reaction between 3 (2 mmol), 2a (2 mmol), malononitrile (2 mmol) and terephtalaldehyde or isophtalaldehyde (1 mmol) in the presence of Fe_3_O_4_@SiO_2_-TCT-theophylline in EtOH under reflux conditions for 30–40 min (Scheme 10).^41^

**Scheme 11:**
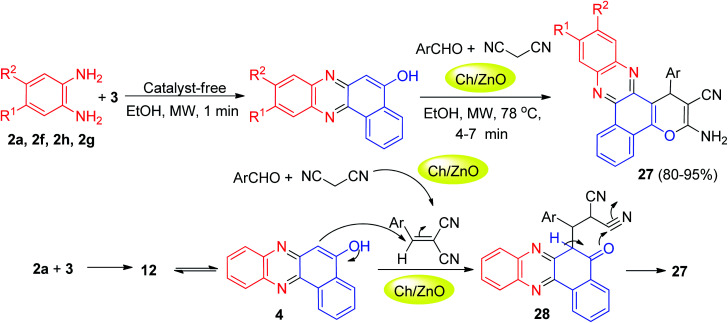
Ch/ZnO catalyzed synthesis of benzo[*a*]pyrano[2,3-*c*]phenazine derivatives 27.

After that, the nanostructured α-chitin/ZnO was used as reusable nanocatalyst in the green synthesis of benzo[*a*]pyrano(2,3-*c*)phenazine derivatives 27 in 80–95% yields through a four-component domino reaction of 3, *o*-phenylenediamines, aromatic aldehydes and malononitrile under microwave irradiation in EtOH at 78 °C within 4–7 min. The mechanism is shown in Scheme 11. 6*H*-Benzo[*a*]phenazin-5-one (12) in tautomeric equilibrium with 4 was obtained after the nucleophilic attack of 2a to 3 followed by dehydration. Subsequent Michael addition of 4 to benzylidenemalononitrile, produced *via* Knoevenagel condensation of arylaldehydes with malononitrile catalyzed by Ch/ZnO, provided the desired product 27 after cyclization of intermediate 28.^42^

**Scheme 12:**
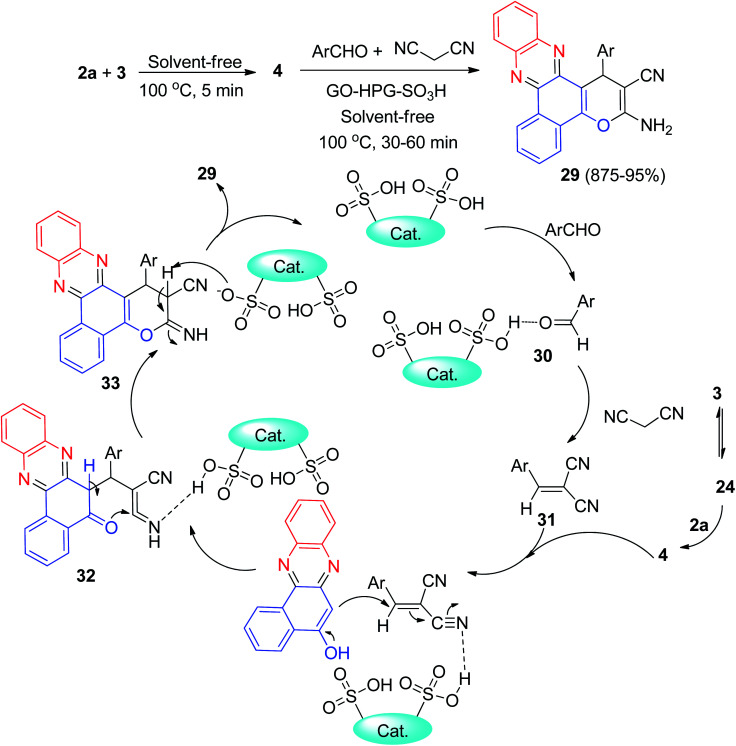
GO-HPG-SO_3_H catalyzed synthesis of benzo[*a*]pyrano-[2,3-*c*]phenazine dyes 29.

In addition, hyperbranched polyglycerol functionalized graphene oxide (GO-HPG-SO_3_H) as an efficient reusable catalyst was employed in the synthesis of benzo[*a*]pyrano-[2,3-*c*]phenazine dyes 29 in 85–95% yields *via* one-pot reaction between 3, 2a, aromatic aldehydes and malononitrile under solvent-free conditions at 100 °C for 30–60 min. A plausible mechanism for the synthesis of 29 is depicted in Scheme 12. Firstly, 3 tautomerizes to intermediate 24. The primary condensation of 24 with 2a gives 4. On this mechanism, the GO-HPG-SO_3_H catalyst activates the carbonyl group of the aromatic aldehyde to afford intermediate 30. The Knoevenagel condensation of 30 and malononitrile forms the arylidene malononitrile 31. Subsequently, the Michael addition of 4 with 31 in the presence of the catalyst gives intermediate 32. The intermediate 32 undergo tautomerization and intramolecular cyclization using the catalyst to form intermediate 33. Ultimately, after tautomerization of intermediate 33, the desired products 29 are formed.^43^

**Scheme 13:**
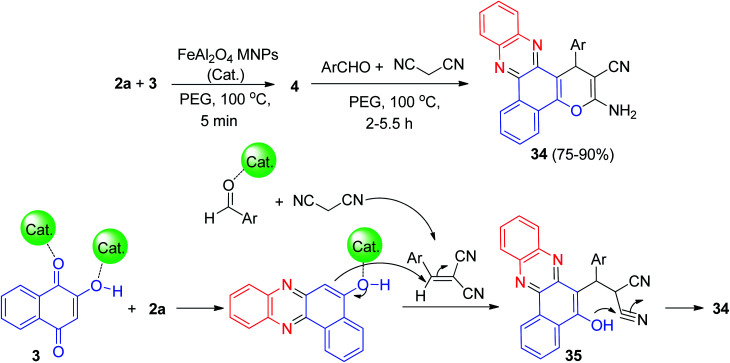
FeAl_2_O_4_ MNPs catalyzed synthesis of benzo[*a*]pyrano[2,3-*c*]phenazines 34.

Further, Ghorbani-Choghamarani and his group synthesized benzo[*a*]pyrano[2,3-*c*]phenazine derivatives 34 in 75–90% yields by the reaction of 3, 2a, aromatic aldehydes/isatine and malononitrile in the presence of spinel FeAl_2_O_4_ (hercynite) magnetic nanoparticles as recyclable catalyst in PEG-400 at 100 °C for 2–5.5 h. The suggested reaction mechanism is depicted in Scheme 13. Initially, intermediate 4 was formed from the Schiff-base condensation of 2a and 3 in the presence of FeAl_2_O_4_ MNPs. Sequentially; a possible intermediate 35 was formed *via* Michael addition of 2-benzylidenemalononitrile. 2-Benzylidenemalononitrile was formed *via* the Knoevenagel condensation of aldehyde with malononitrile. Finally, intermolecular cyclization of intermediate 35 produced a final product 34.^44^

**Scheme 14:**
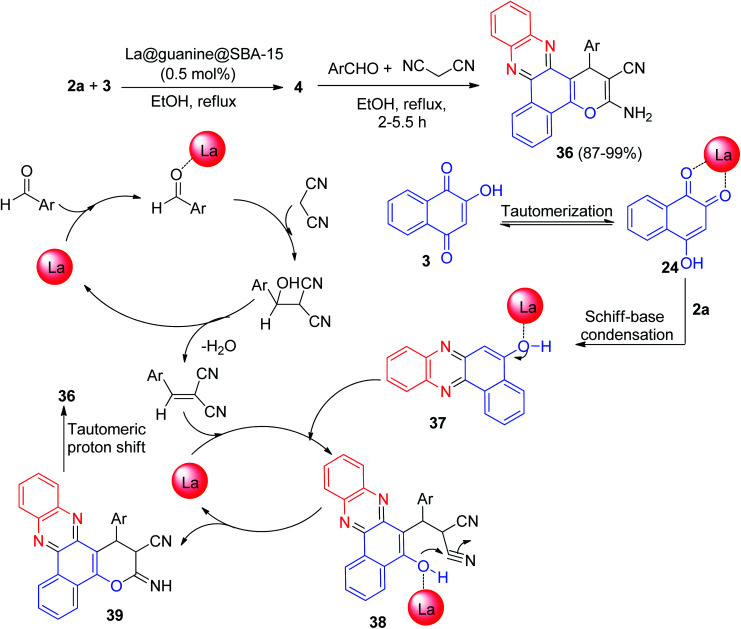
La@guanine@SBA-15 catalyzed synthesis of benzo[*a*]pyrano[2,3-*c*]phenazine derivatives 36.

In 2020, Nikoorazm *et al.* synthesized benzo[*a*]pyrano[2,3-*c*]phenazine derivatives 36 in 87–99% yields by the reaction of 3, 2a, aromatic aldehydes/isatine and malononitrile in the presence of La@guanine@SBA-15 (0.5 mol%), in EtOH at reflux conditions for 2–5.5 h. The suggested reaction mechanism has been depicted in Scheme 14. Initially, the intermediate 37 was formed from the Schiff-base condensation of 2a and 3 in the presence of the catalyst. Sequentially, a possible intermediate 38 was formed *via* Michael addition of 2-benzylidenemalononitrile with 37. In the next step, intermolecular cyclization of intermediate 38 produced intermediate 39. Finally, a tautomeric proton shift produced the final product 36.^45^

**Scheme 15:**
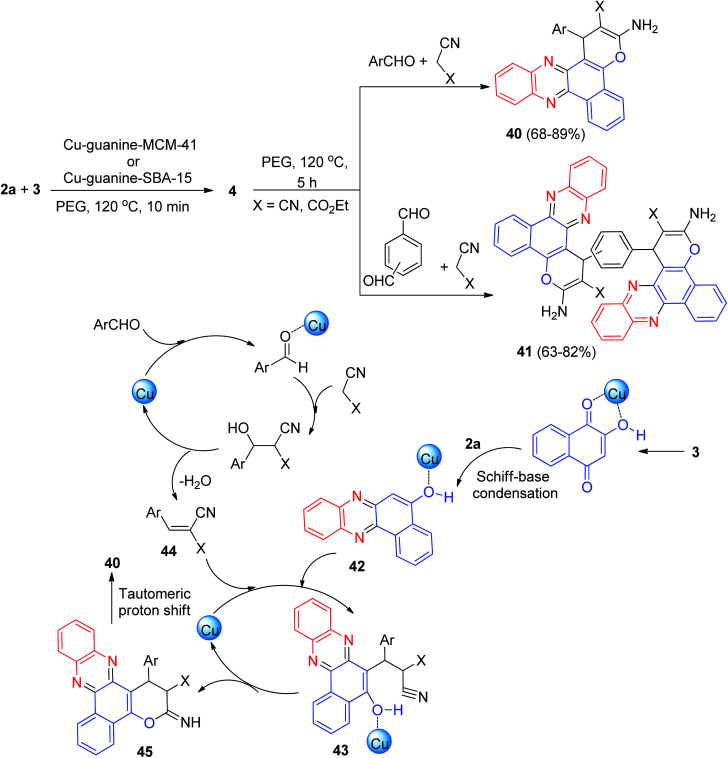
Preparation of benzo[*c*]pyrano[3,2-*a*]phenazines 40–41.

Next, Nikoorazm and Khanmoradi described the preparation of benzo[*c*]pyrano[3,2-*a*]phenazines 40 in 68–89% yields and bis-benzo[*c*]pyrano[3,2-*a*]phenazine derivatives 41 in 63–82% yields by the one-pot, two-step, four-component reaction of 3, 2a, carbonyl compounds and alkylmalonates using copper(ii) ions complexes of guanine (2-amino-1*H*-purin-6(9*H*)-one) supported into MCM-41(Cu-guanine-MCM-41) and SBA-15 (Cu-guanine-SBA-15) channels as efficient and heterogeneous catalysts in PEG at 120 °C for 5 h. Scheme 15 depicts the possible reaction mechanism for the synthesis of 40. Initially, the intermediate 42 was formed from the Schiff-base condensation of 2a and 3 in the presence of the catalyst. Sequentially, a possible intermediate 43 was formed *via* Michael addition of 44 with 42 (intermediate 44 is formed *via* the Knoevenagel condensation of aldehyde with malononitrile in the presence of Cu-guanine-MCM-41 and Cu-guanine-SBA-15 catalysts). In the next step, intermolecular cyclization of intermediate 43 produced intermediate 45. Ultimately, a tautomeric proton shift produced the final product 40.^46^

**Scheme 16:**
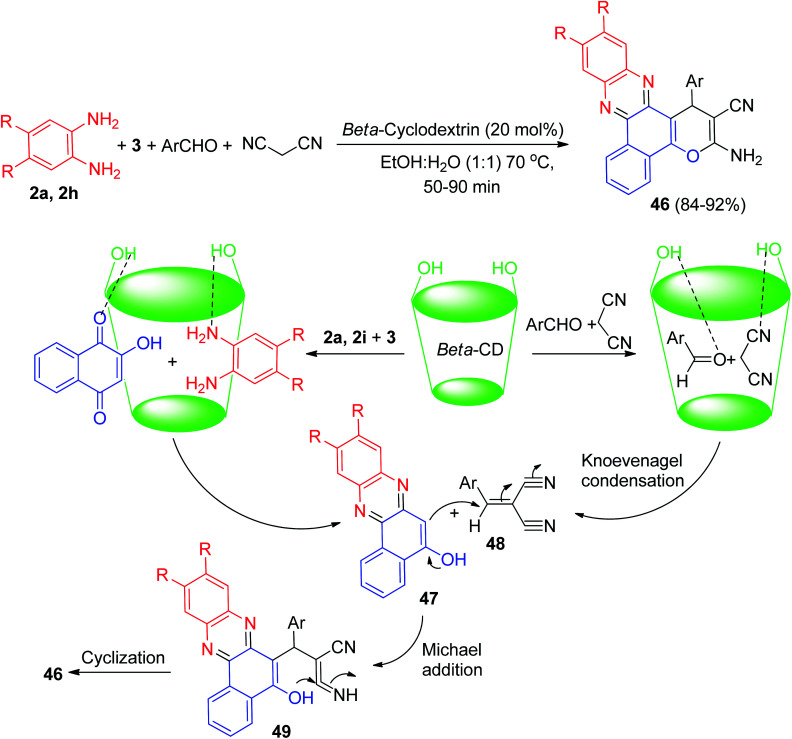
β-CD catalyzed synthesis of benzo[*a*]pyrano[2,3-*c*]phenazine derivatives 46.

Further, Singh *et al.* described a pragmatic and swift method for the synthesis of benzo[*a*]pyrano[2,3-*c*]phenazine derivatives 46 in 84–92% yields *via* one-pot, multi-component reaction of 3, benzene-1,2-diamines, aromatic aldehydes and malononitrile in the presence of supramolecular β-cyclodextrin as a biodegradable and reusable catalyst in EtOH : H_2_O (1 : 1) solvent at 70 °C for 50–90 min. A plausible reaction mechanism is depicted in Scheme 16. The desired product is expected to form by the Knoevenagel condensation followed by Michael addition and at last cyclization within the cavity of β-CD where it is anticipated that seven free primary –OH groups of β-CD execute synergistically as a proficient host and supramolecular catalyst. Initially, the condensation of 3 and diamine takes place to afford the intermediate 47. Similar condensation of aldehyde and malanonitrile occurs to form the intermediate 48. After that, intermediate 47 reacts with intermediate 48*via* Michael addition to yield an intermediate 49. Finally, intermediate 49 undergo cyclization to afford the desired product 46.^47^

**Scheme 17:**
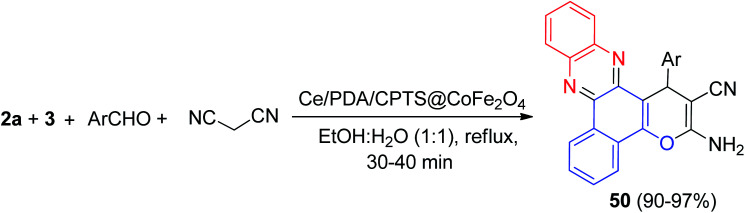
Synthesis of benzo[*a*]pyrano[2,3-*c*]phenazine derivatives 50.

In 2021, Heravi and his co-workers reported synthesis of benzo[*a*]pyrano[2,3-*c*]phenazine derivatives 50 in 90–97% yields by condensing 3, 2a, malononitrile and different aryl aldehydes in the presence of Ce/PDA/CPTS@CoFe_2_O_4_ as a nanocomposite catalyst in EtOH : H_2_O (1 : 1) under reflux conditions for 30–40 min (Scheme 17). In the proposed mechanism, initially, 3, an enolated 1,2-diketo compound, being activated by the Lewis acidity of Ce^4+^ ions attached on the catalyst surface, undergoes condensation with 2a to generate the orange colored benzo[*a*]phenazin-5-ol. In the mean time, Ce ions also trigger the aromatic aldehyde to condense with malononitrile, an active methylene compound, to form the Knoevenagel adduct. Now, benzo[*a*]phenazin-5-ol, adds up to the Knoevenagel adduct, being an excellent activated Michael acceptor, following 1,4-addition, intramolecular cycloacondensation affords the final desired product 50.^48^

**Scheme 18:**
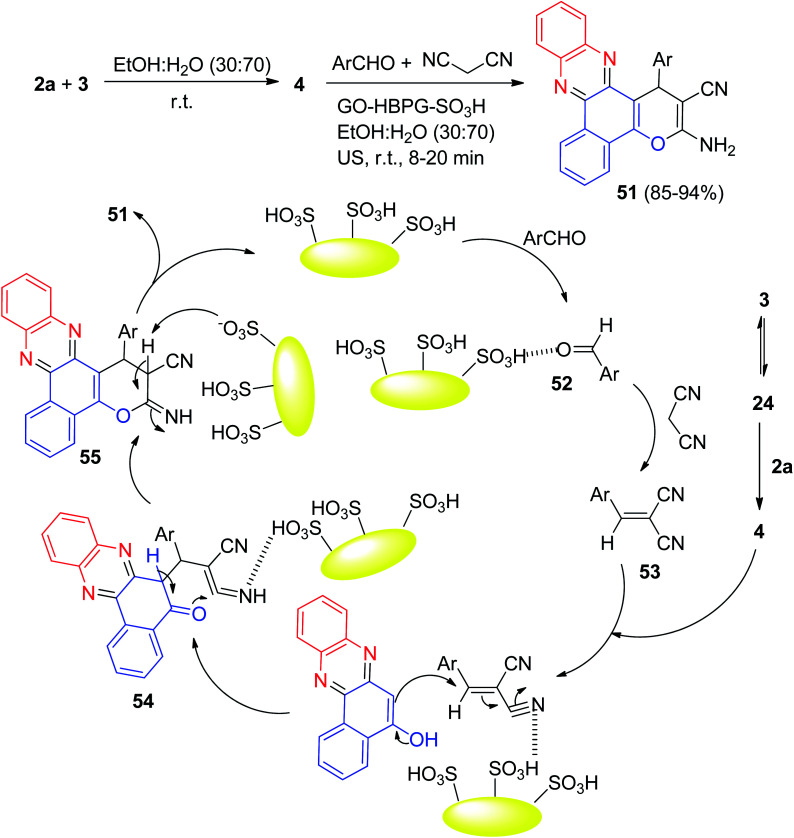
Preparation of the benzo[*a*]-pyrano[2,3-*c*]phenazines 51.

After that, a green and rapid sonochemical research to preparation of the benzo[*a*]-pyrano[2,3-*c*]phenazines 51 in 85–94% yields was carried out through a four-component reaction of 3, 2a, aldehydes, and malononitrile by using multisulfonic acid hyperbranched polyglycerol modified graphene oxide (GO-HBPG-SO_3_H) as an effective and recyclable nanocatalyst in EtOH : H_2_O (30 : 70) under ultrasonic irradiation at 45 kHz at room temperature for 8–20 min. A possible mechanism for the synthesis of 262 is outlined in Scheme 18. Firstly, 3 tautomerizes to intermediate 24. The primary condensation of intermediate 24 with 2a obtains 4. On this mechanism, the GO-HBPG-SO_3_H catalyst activates the carbonyl group of the aromatic aldehyde to afford intermediate 52. The Knoevenagel condensation of intermediate 52 and malononitrile forms the arylidene malononitrile 53. Subsequently, the Michael addition of 4 with intermediate 53 in the presence of GO-HBPG-SO_3_H catalyst gives intermediate 54. The intermediate 54 undergo tautomerization and intramolecular cyclization using the catalyst to form intermediate 55. Ultimately, after tautomerization of intermediate 55, the desired products 51 are formed.^49^

**Scheme 19:**
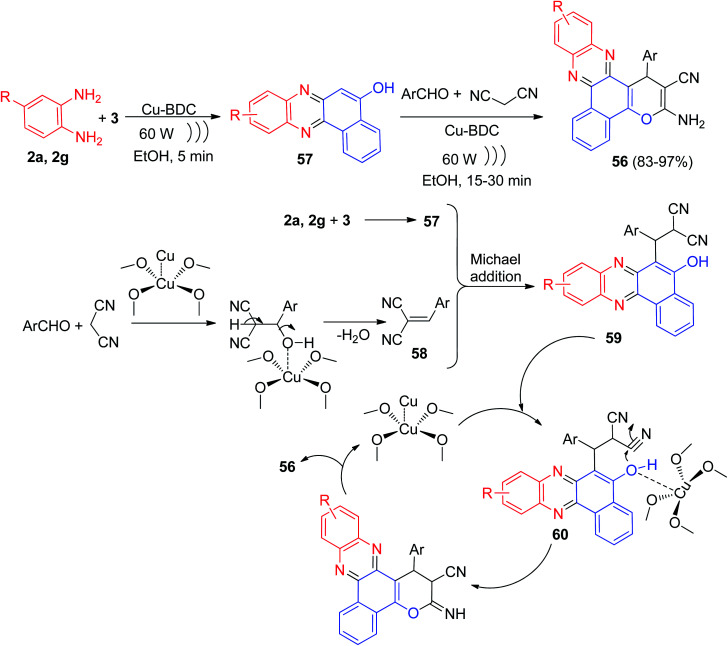
Cu-BDC catalyzed synthesis of benzophenazine derivatives 56.

Further, Taheri and his group described the reaction of 3 with benzene-1,2-diamines, aldehydes and malononitrile in the presence of Cu-benzene dicarboxylic acid (Cu-BDC) under ultrasonic irradiation at 60 W power for 15–30 min afforded benzophenazine derivatives 56 in 83–97% yields. The proposed mechanism for the production of 56 is presented in Scheme 19. In the first stage, the condensation of 3 and *o*-phenylenediamines leads to the production of benzo[*a*]phenazin-5-ol 57. Additionally, the Knoevenagel condensation aldehyde and malononitrile in the presence of Cu-BDC as acid catalyst produce intermediate 58. The Michael addition of 57 with 58 from Knoevenagel condensation leads to the production of 59 that occurs in the presence of acidic Cu-BDC and an intermediate 60 is produced. Finally, in the course of a cyclization followed by tautomerism, the final product 56 produced and the catalyst returns to the reaction cycle.^50^

**Scheme 20:**
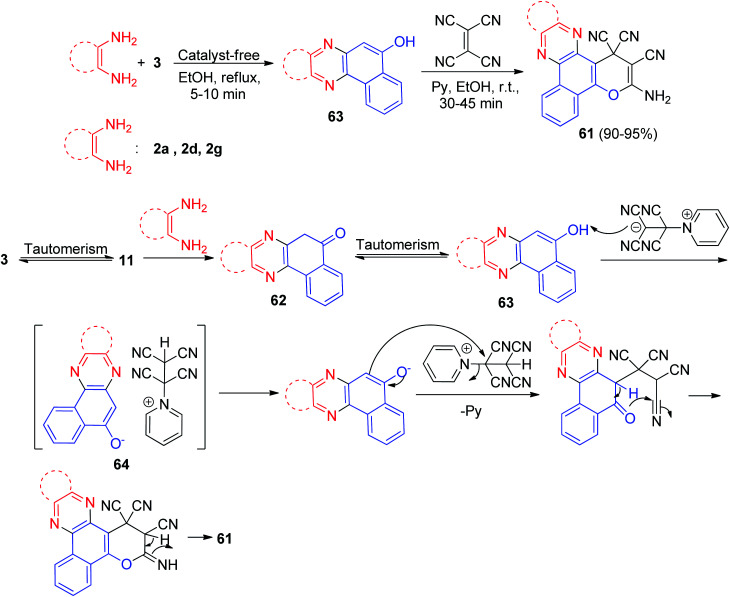
Synthesis of benzo[*a*]pyrano[2,3-*c*]phenazines 61.

In 2016, benzo[*a*]pyrano[2,3-*c*]phenazine derivatives 61 were synthesized in 90–95% yields *via* a one-pot, two-step procedure from a three-component condensation reaction of 3, 1,2-diamines, and tetracyanoethylene in the presence of pyridine (20 mol%) as an efficient catalyst in EtOH at room temperature for 30–45 min. The mechanism for the formation of the products is proposed in Scheme 20. On the basis of this mechanism, at first, 3 tautomerizes to intermediate 11. The primary condensation of 11 with 1,2-diamine gives compound 62, which in tautomerism equilibrium helps to prepare compound 63. Then, based on the nucleophilicity of pyridine, the nucleophilic addition of pyridine to the electron-deficient tetracyanoethylene and subsequent protonation in the presence of compound 63 gives intermediate 64, followed by the attack of the anion on the cation part of intermediate 64 to form the product 61*via* intramolecular cyclization and a tautomeric proton shift.^51^

**Scheme 21:**
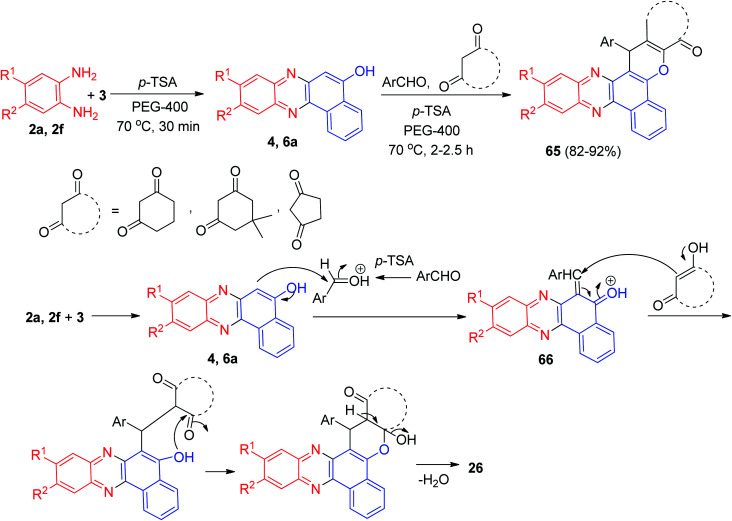
Synthesis of fluorescent benzo[*a*]phenazine fused derivatives 65.

In 2012, one-pot two-step domino protocol for the efficient synthesis of fluorescent benzo[*a*]-phenazine fused derivatives 65 in 82–92% yields was developed. The synthesis was achieved by reacting 3, *ortho*-phenylenediamines, aromatic aldehydes and cyclic 1,3-dicarbonyl compounds in the presence of a catalytic amount of *p*-TSA in PEG-400 at 70 °C for 2–2.5 h. A speculative mechanistic explanation for this reaction is provided in Scheme 21. The formation of 65 proceeds *via* initial condensation of 3 and diamine to afford benzo[*a*]phenazin-5-ol derivatives 4 and 6a as reported which *in situ* generates an *ortho*-quinone methide (*o*-QM) intermediate 66 upon nucleophilic addition to aldehyde. Subsequent Michael addition of the *o*-QM with a cyclic 1,3-dicarbonyl compound, followed by cyclization and dehydration leads to the formation of 65.^52^

**Scheme 22:**
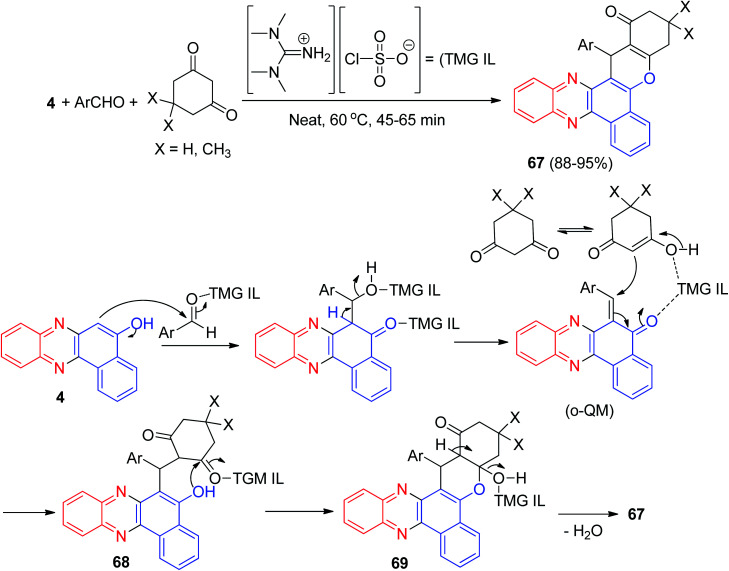
Synthesis of tetrahydro-1*H*-benzo[*a*]chromeno[2,3-*c*]phenazin-1-ones 67.

In 2015, Jeong and co-workers reported a synthetic route to produce tetrahydro-1*H*-benzo[*a*]-chromeno[2,3-*c*]phenazin-1-ones 67 in 88–95% yields by the straightforward, efficient and convenient approach of a three-component reaction between aromatic aldehydes, 4 and active methylene compounds under neat conditions in the presence of an ionic liquid, tetramethyl guanidiniumchlorosulfonate (TMG IL), at 60 °C for 45–65 min. The TMG IL was used as a solvent and as a catalyst under reusable conditions. The title compounds were screened for their *in vitro* antioxidant activity and it was found that most of the compounds are effective against reactive oxygen species. The majority of them also have excellent *in vitro* anti-cancer activity on two human cancer cell lines, HeLa and SK-BR-3, compared with standard drugs. The TMG IL-catalyzed synthetic sequence of the title compounds is presented in Scheme 22, and may proceed *via* an *ortho*-quinone methide (*o*-QM) intermediate. At the beginning, nucleophilic addition of 4 to an aldehyde takes place and subsequently Michael addition of the *o*-QM to an enolic form of a cyclic 1,3-dicarbonyl, followed by the addition of the benzyl hydroxy moiety to the carbonyl of the ketone 68, provides a cyclic hemiketal 69, which on dehydration affords 67.^53^

**Scheme 23:**
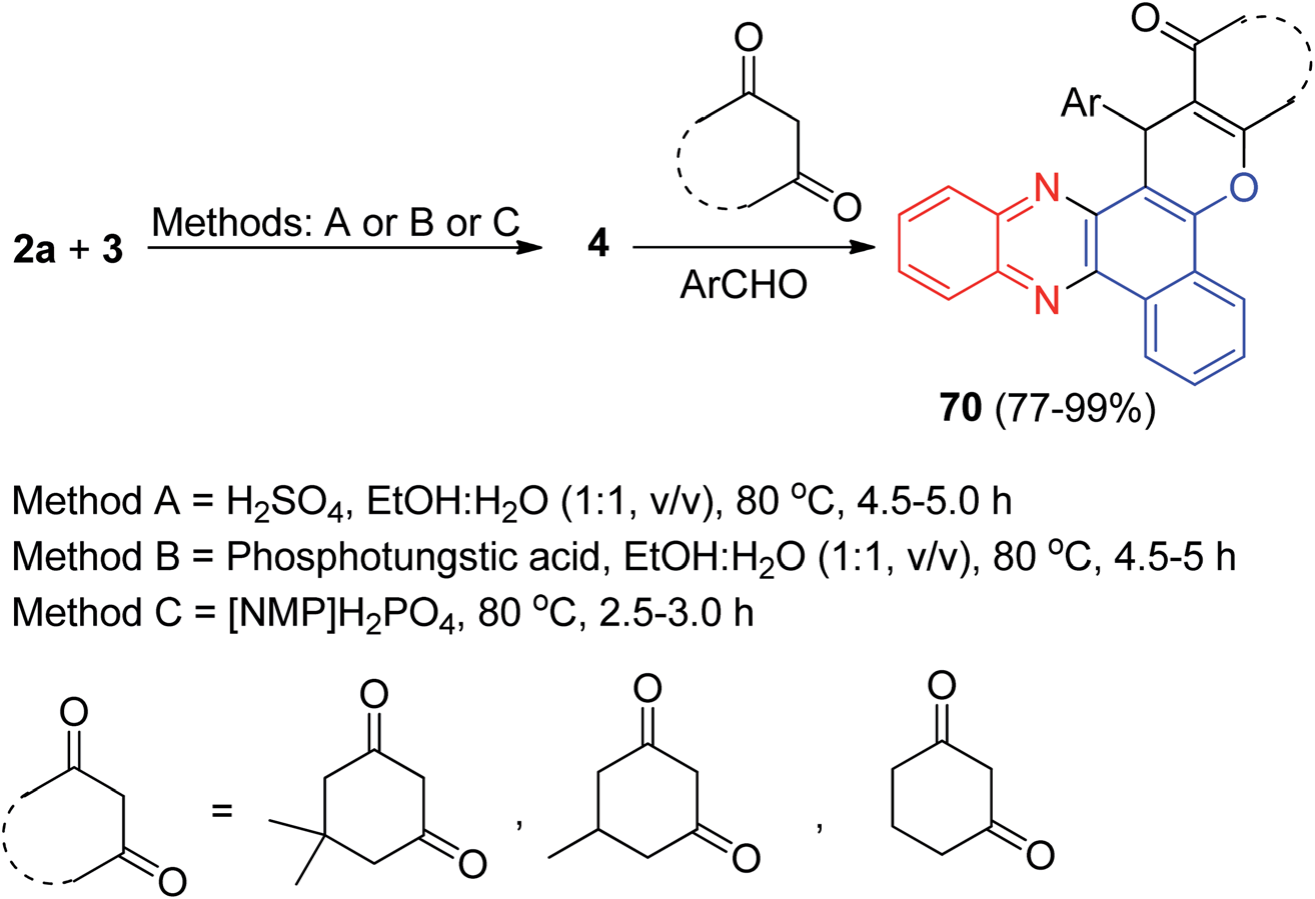
Synthesis of functionalized benzo[*c*]chromeno[2,3-*a*]phenazine derivatives 70.

Next, an efficient and quantitative procedure for the synthesis of functionalized benzo[*c*]chromeno[2,3-*a*]phenazine derivatives 70 in 77–99% yields by one-pot, two-step four-component condensation of 3, 2a, aromatic aldehydes, and cyclic 1,3-dicarbonyl compounds were developed using catalytic amounts of H_2_SO_4_ and phosphotungstic acid in EtOH/H_2_O (1 : 1) under reflux and also with Brønsted acidic ionic liquid [NMP]H_2_PO_4_, which acts as catalyst and medium at 80 °C (Scheme 23).^54^

**Scheme 24:**
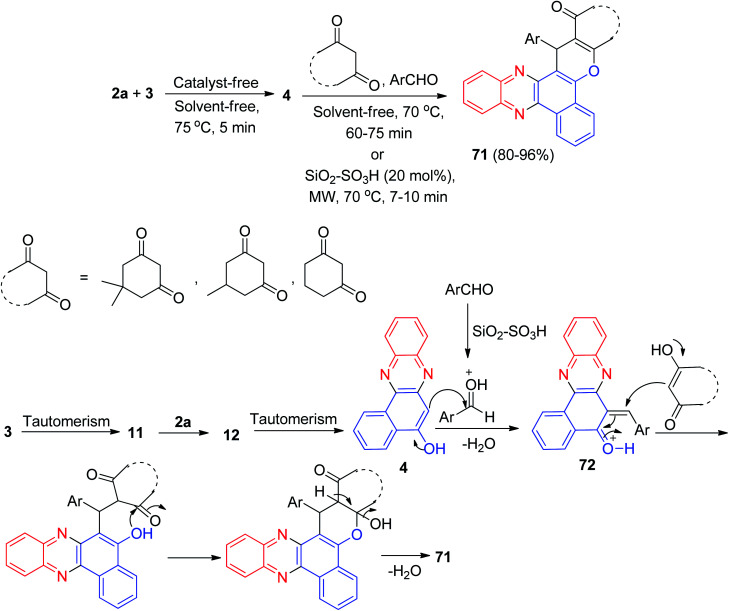
Synthesis of benzo[*a*]chromeno[2,3-*c*]phenazines 71.

In 2018, silica sulfuric acid (SiO_2_–SO_3_H) has been used as an effective and reusable solid catalyst for the one-pot, two-step, four-component synthesis of benzo[*a*]chromeno[2,3-*c*]phenazine derivatives 71 by the condensation reaction of 3, 2a, aldehydes, and cyclic 1,3-dicarbonyl compounds. The reaction was carried out under conventional heating at 70 °C or microwave irradiation at 70 °C afforded the desired products 71 in 60–75 min with 80–87% and 7–10 min with 88–96% yields, respectively. The probable mechanism is given in Scheme 24. On the basis of this mechanism, at first, 3 tautomerizes to intermediate 11. The primary condensation of 11 with 2a gives 6*H*-benzo[*a*]phenazin- 5-one 12, which in tautomerism equilibrium reasons to prepare 4. On this mechanism, SiO_2_–SO_3_H is an efficient catalyst to form (6-benzylidenebenzo[*a*]phenazin-5(6*H*)-ylidene)oxonium 72, which easily prepares *in situ* from condensation of aldehyde with 4. Subsequent Michael addition of cyclic 1,3-dicarbonyl compounds with 72, followed by cyclization and dehydration leads to the formation of product 71.^55^

**Scheme 25:**
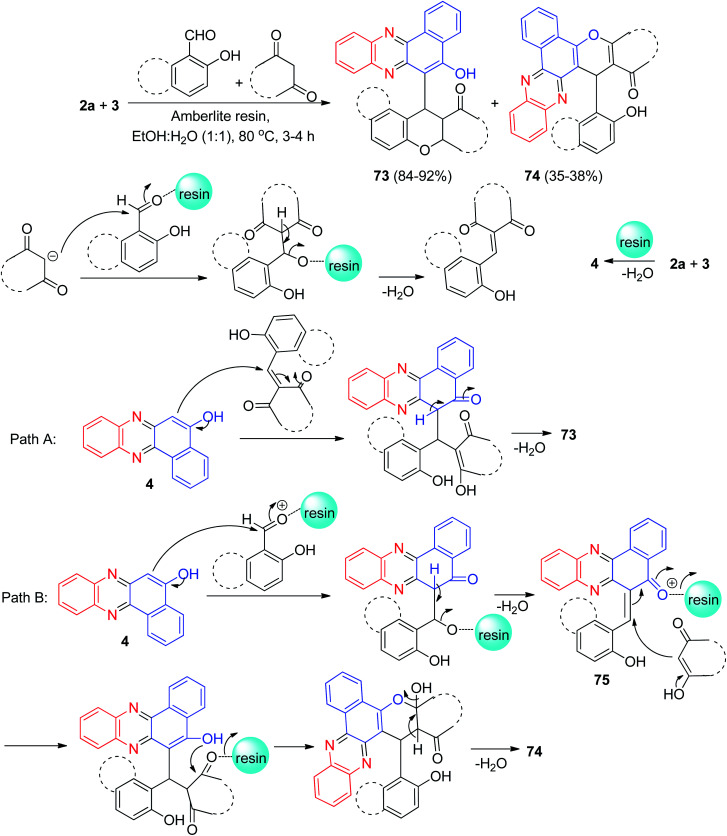
Synthesis of 4*H*-chromenes and benzo[*a*]chromenophenazines 73–74.

After that, Harichandran and co-workers developed an efficient protocol for the synthesis of fluorescent 4*H*-chromenes and benzo[*a*]chromenophenazines 73–74 in 35–92% yields starting from the reaction of 2a, 3, 2-hydroxy benzaldehydes, and 1,3-diketones in EtOH/H_2_O (1/1, v/v) as solvent in the presence of Amberlite resin at 80 °C for 3–4 h. These compounds have been found to be good photophysical properties such as solvatochromism, absorption, emission, Stocks shift and quantum yield, fluorescent chemosensors and metal ion sensors for the detection of Fe^3+^ and Cu^2+^ ions. A plausible mechanism for the formation of compounds 73–74 with Amberlite IR-120 H^+^ resin has been proposed in Scheme 25. Initially, condensation of 2a and 3 gives 4. Next two possible pathways of mechanism (pathway-A and pathway-B) are possible. Pathway-A explains the formation of compounds 73. In pathway B, the *in situ* generated *o*-quinonemethide (*o*-QM) intermediate 75 is believed to form from 4 upon nucleophilic addition of salicylaldehyde. Subsequent Michael addition of intermediate 75 to diketone followed by dehydration affords benzo[*a*]chromenophenazines 74.^56^

**Scheme 26:**
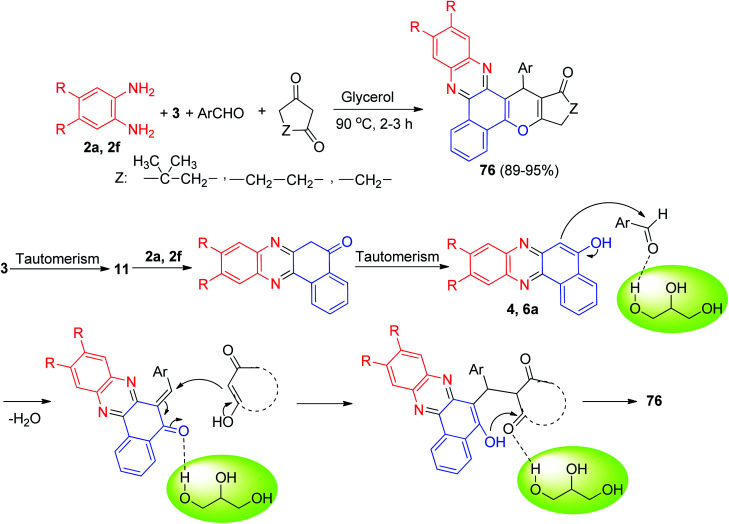
Preparation of benzo[*a*]chromeno[2,3-*c*]phenazine derivatives 76.

In 2020, Siddiqui *et al.* described the preparation of benzo[*a*]chromeno[2,3-*c*]phenazine derivatives 76 in 89–95% yields through an efficient one-pot, multi-component ecofriendly reaction of 3, *o*-phenylenediamines, cyclic 1,3-dicarbonyl compounds and aromatic aldehydes, promoted by glycerol at 90 °C for 2–3 h. A plausible mechanism for the disclosed synthetic transformation has been proposed in Scheme 26. The reaction is presumed to initiate *via* Knoevenagel condensation of 3 and *o*-phenylenediamines resulting in benzo[*a*]phenazin-5-ol derivative 4 and 6a as the first intermediate. A second Knoevenagel condensation of 4 or 6a with aromatic aldehydes leads to benzo[*a*]phenazin-5(6*H*)-ones. Finally, cyclization resulting from Michael attack of benzo[*a*]phenazin-5(6*H*)-ones on cyclic 1,3-dicarbonyl compounds followed by dehydration results in the desired product 76.^57^

**Scheme 27:**
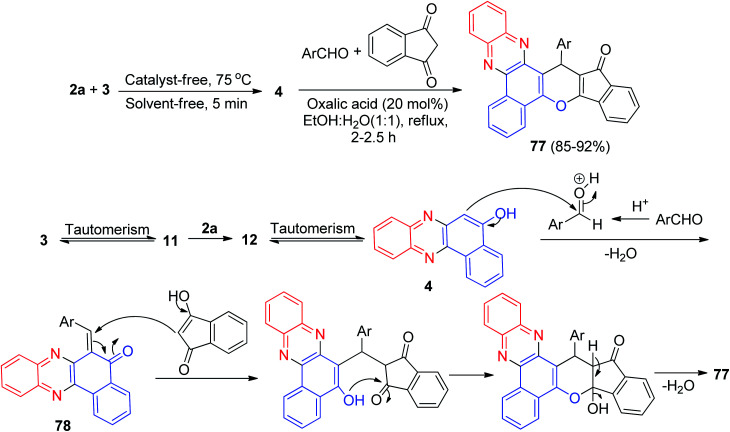
Oxalic acid catalyzed synthesis of 16-(aryl)benzo[*a*]indeno[2′,1′:5,6]pyrano[2,3-*c*]phenazin-15(16*H*)-ones 77.

In 2016, a sequential one-pot two-step four-component reaction for the efficient synthesis of 16-(aryl)benzo[a]indeno[2′,1′:5,6]pyrano[2,3-c]phenazin-15(16*H*)-one derivatives 77 in 85–92% yields was developed. The synthesis was achieved by reacting 3, 2a, aromatic aldehydes, and 1,3-indandione in the presence of oxalic acid (20 mol%) as a reusable and homogeneous organocatalyst in EtOH/H_2_O (1 : 1) under reflux for 2–2.5 h. A reaction mechanism is shown in Scheme 27. Oxalic acid plays a key role as a Brønsted-Lowry acid catalyst in this reaction. The formation of 77 proceeds *via* initial condensation of 3 and 2a to afford 4 as reported, which *in situ* generates an *ortho*-quinone methide (*o*-QM) intermediate 78 upon nucleophilic addition to aldehyde. Subsequent Michael addition of the *o*-QM with 1,3-indandione, followed by cyclization and dehydration, leads to the formation of product 77.^58^

**Scheme 28:**
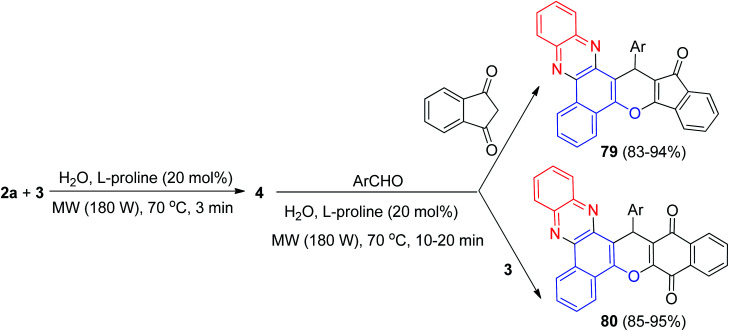
Microwave-assisted synthesis of benzo[*a*]phenazine annulated heterocycles 79–80.

After that, a highly efficient one-pot, two-step microwave-assisted procedure was applied for the rapid and green synthesis of benzo[*a*]phenazine annulated heterocyclic ring systems 79 in 83–94% and 80 in 85–95% yields from the three- or four-component condensation reactions of 3, 2a, aromatic aldehydes and 1,3-indandione or 3 using l-proline as a bifunctional organocatalyst in water at 70 °C for 10–20 min (Scheme 28). Moreover, the catalyst can be recovered and reused several times without much loss of its performance. Also, the reactions were examined with aliphatic aldehydes such as *n*-heptanal and *n*-octanal but the related products were not obtained in these reaction conditions even after 20 min. The probable mechanism for the domino synthesis of 79 and 80 using l-proline is similar to the proposed mechanism in Scheme 28.^59^

**Scheme 29:**
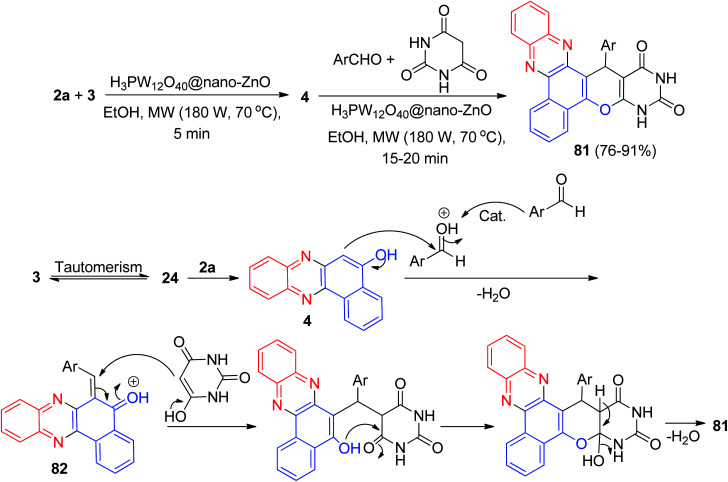
Domino synthesis of benzo[*a*]pyrimido[5′,4′:5,6]pyrano[2,3-*c*]phenazines 81.

In 2018, Mohebat and co-workers developed single-pot synthesis of heteroaryl-substituted benzo[*a*]pyrimido[5′,4′:5,6]pyrano[2,3-*c*]phenazines 81 in 76–91% yields *via* initial Knoevenagel, subsequent Michael, and final heterocyclization reactions of 3, 2a, aromatic aldehydes, and barbituric acid in the presence of H_3_PW_12_O_40_@nano-ZnO as a recyclable heterogeneous catalyst in EtOH under microwave irradiation (180 W, max. 70 °C) for 15–20 min. The proposed mechanism for this four-component sequential reaction is shown in Scheme 29. H_3_PW_12_O_40_@nano-ZnO plays a key role as a Brønsted acid catalyst in this reaction. The formation of 81 proceeds *via* initial condensation of 3 and 2a to afford 4 as reported, which *in situ* gives the *ortho*-quinone methide (*o*-QM) intermediate 82 upon nucleophilic addition to benzaldehyde. Subsequent Michael addition of the *o*-QM with barbituric acid, followed by cyclization and dehydration leads to the formation of the desired product 81.^60^

**Scheme 30:**
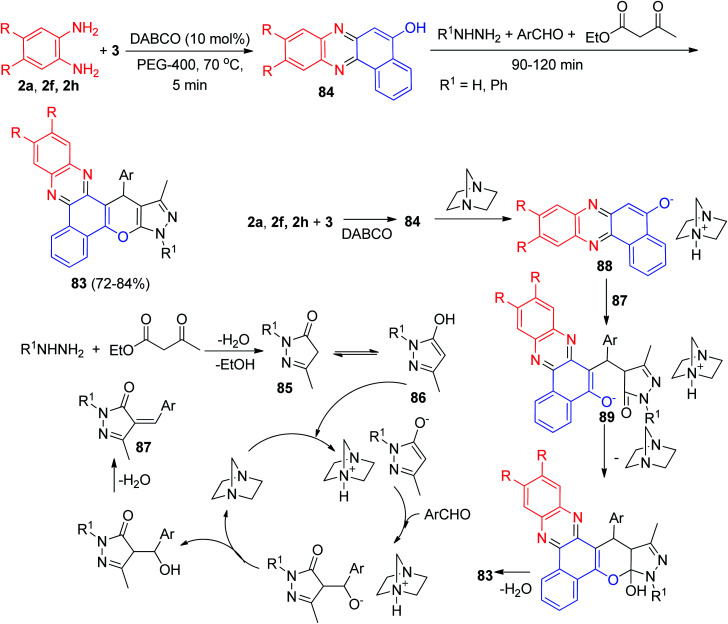
Synthesis of benzo[*a*]pyrazolo[4′,3′:5,6]pyrano[2,3-*c*]phenazine derivatives 83.

In 2020, DABCO-catalyzed five-component domino protocol for the synthesis of benzo[*a*]pyrazolo[4′,3′:5,6]pyrano[2,3-*c*]phenazines 83 in 72–84% yields has been reported by Mohebat and co-workers. The condensation reaction of 3, benzene-1,2-diamines, hydrazines, aromatic aldehydes and ethyl acetoacetate was carried out in PEG-400 as a green catalyst in the presence of BABCO (10 mol%) at 70 °C for 90–120 min. A detailed reaction mechanism is outlined in Scheme 30. The primary condensation of 3 with benzene-1,2-diamine in the presence of DABCO gives benzo[*a*]phenazin-5-ol 84. Then, hydrazine condenses with ethyl acetoacetate to generate the pyrazolone ring 85, which is then isomerized to intermediate 86. In this mechanism, DABCO is a catalyst to form the olefin 87, which is readily formed *in situ* from the Knoevenagel condensation of aromatic aldehyde with pyrazole 86. In the presence of DABCO, 84 converts to its corresponding enolate form 88, to react easily with olefin 87 (Michael addition) and give intermediate 89, which then produces 83.^61^

**Scheme 31:**
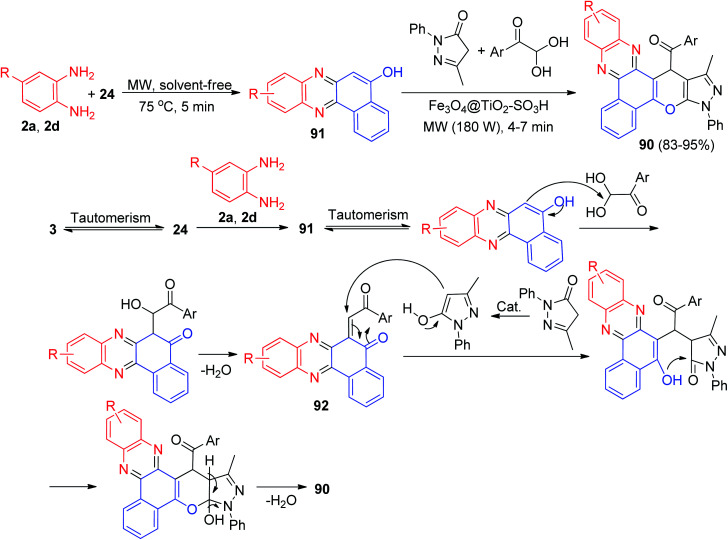
Synthesis of (pyrazolo[4′,3′:5,6]pyrano[2,3-*c*]phenazin-15-yl)methanone derivatives 90.

Next, one-pot method for the synthesis four-component of (pyrazolo[4′,3′:5,6]pyrano[2,3-*c*]phenazin-15-yl)methanone derivatives 90 in 83–95% yields has been developed by the reaction of 24, benzene-1,2-diamines, 5-methyl-2-phenyl-2,4-dihydro-3*H*-pyrazol-3-one and arylglyoxal derivatives in the presence of nano Fe_3_O_4_@TiO_2_–SO_3_H as a recoverable magnetic catalyst under microwave irradiation (180 W) and in a solvent-free environment at 75 °C for 4–7 min. A plausible rational mechanism is illustrated under the results in Scheme 31. Based on this mechanism, at first, 3 tautomerizes to intermediate 24. The primary condensation of 24 with benzene-1,2-diamine obtain benzo[*a*]phenazin-5-ol 91. Then, 91 condenses with arylglyoxal derivatives to generate intermediate 92 after dehydration. Subsequent Michael addition of 3-methyl-1-phenyl-1H-pyrazol-5-ol with intermediate 92, followed by cyclization and dehydration leads to the formation of product 90.^62^

**Scheme 32:**
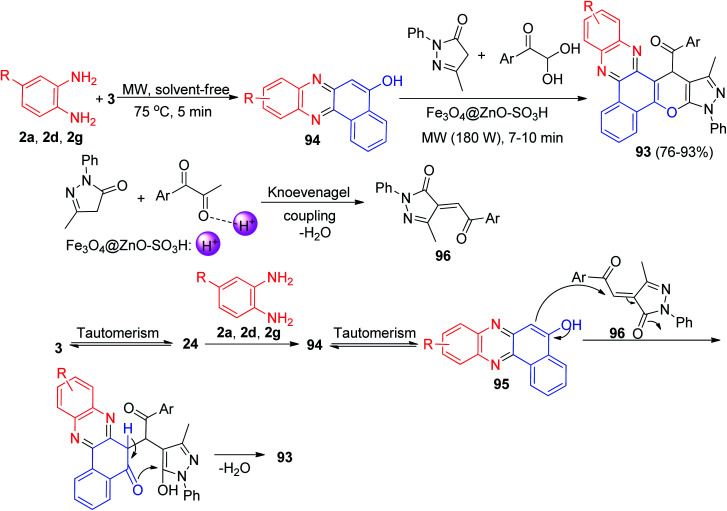
Preparation of (pyrano[2,3-*c*]phenazin-15-yl)methanone derivatives 93.

After that, (pyrano[2,3-*c*]phenazin-15-yl)methanone derivatives 93 were prepared in 76–93% yields by the reaction of 3, benzene-1,2-diamines, 5-methyl-2-phenyl-2,4-dihydro-3*H*-pyrazol-3-one and arylglyoxal in the presence of Fe_3_O_4_@ZnO–SO_3_H as a recyclable heterogeneous catalyst under solvent-free conditions, using microwave irradiation (180 W, 75 °C) for 7–10 min. A plausible mechanism is illustrated in Scheme 32. Based on this mechanism, at first, 3 tautomerizes to intermediate 24. The primary condensation of 24 with diamine gives 6*H*-benzo[*a*]-phenazin-5-one 94, which in tautomerizes to benzo[*a*]phenazin-5-ol 95. On the other hand, intermediate 96 was generated by nucleophilic addition of pyrazol to the arylglyoxal after elimination of water. Subsequent Michael addition of 95 to the intermediate 96, followed by cyclization and dehydration leads to the formation of product 93.^63^

**Scheme 33:**
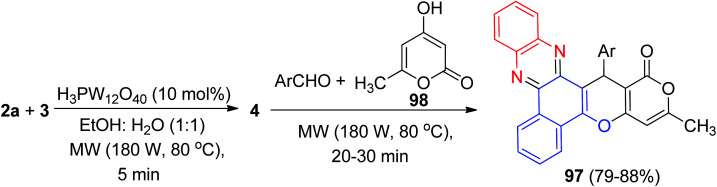
Microwave-assisted synthesis of benzo[*a*]pyrano[3′,4′:5,6]pyrano[2,3-*c*]phenazines 97.

Mohebat *et al.* constructed benzo[*a*]pyrano[3′,4′:5,6]pyrano[2,3-*c*]phenazines 97 in 79–88% yields by the reaction of 3, 2a, benzaldehydes and 4-hydroxy-6-methyl-2*H*-pyran-2-one 98 in the presence of phosphotungstic acid (H_3_PW_12_O_40_) under microwave irradiation (180 W, max. 70 °C) in EtOH/H_2_O (1 : 1) for 20–30 min (Scheme 33).^64^

**Scheme 34:**
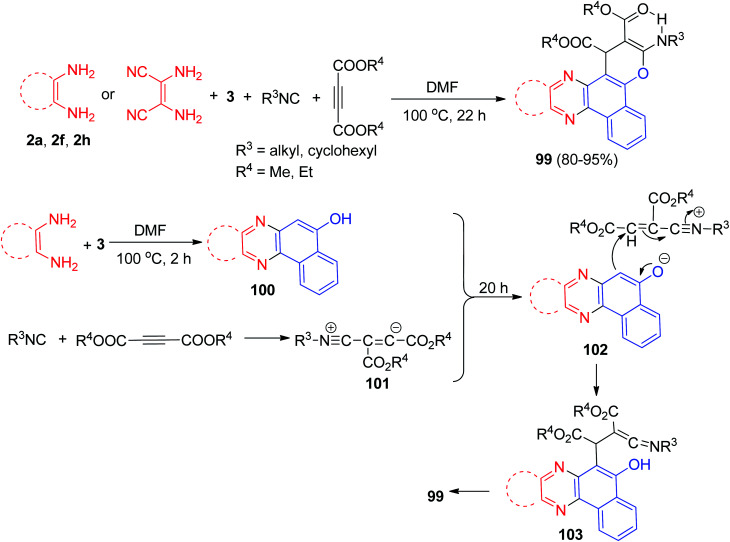
Synthesis of benzo[*a*]pyrano[2,3-*c*]phenazines 99.

Bazgir *et al.* described simple method for the synthesis of biologically interesting benzo[*a*]pyrano[2,3-*c*]phenazines 99 in 80–95% yields by a one-pot two step four-component reaction of 3, diamines, isocyanides, and dialkyl acetylenedicarboxylates in *N*,*N*-dimethylformamide at 100 °C for 22 h. A plausible mechanism for the synthesis of 99 has been shown in Scheme 34. First, the condensation of diamine and 3 gave intermediate 100. Then, the 1 : 1 zwitterionic ionic intermediate 101, formed from the isocyanide and the acetylenic ester, is protonated by 100 to furnish intermediate 102, which is attacked by the anion of the CH-acidic 102 in a Michael fashion to produce ketenimine 103. The latter then can undergo cyclization under the reaction conditions to afford the desired product 99.^65^

**Scheme 35:**
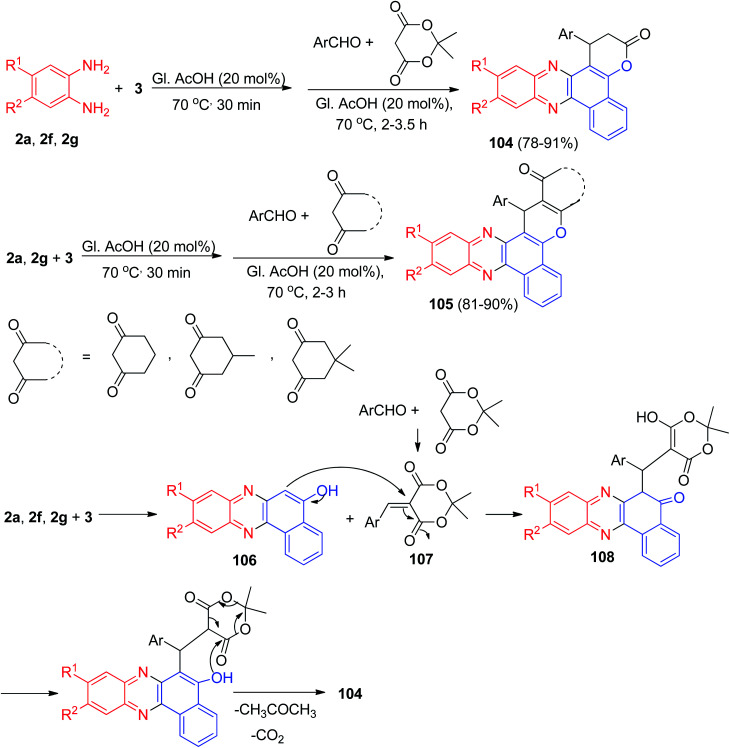
Synthesis of benzo[*a*]pyrano[2,3-*c*]phenazines 104 and benzo[*a*]chromeno[2,3-*c*]phenazines 105.

After that, Khurana *et al.* have published synthesis of fluorescent benzo[*a*]pyrano[2,3-*c*]phenazines 104 in 78–91% yields *via* one-pot, two-step condensation of 3, 1,2-phenylenediamines, aromatic aldehydes, and Meldrum's acid in glacial acetic acid as catalyst at 70 °C for 2–3.5 h. Photophysical studies of these compounds have been reported. Moreover, reactions involving cyclohexane-1,3-dione/5-methylcyclohexane-1,3-dione/dimedone in the place of Meldrum's acid yielded corresponding benzo[*a*]chromeno[2,3-*c*]phenazine derivatives 105 in 81–90% yields after 2–3 h. The synthesis of 104 is believed to be proceeding *via* sequential condensation, Michael addition, cyclization, and elimination (Scheme 35). Initially, 3 and 1,2-phenylenediamine undergo condensation to afford benzo[*a*]phenazin-5-ol 106. Simultaneously the Knoevenagel condensation between an aldehyde and Meldrum's acid yields the arylidene Meldrum's acid 107. Subsequently 106 undergoes Michael type addition to arylidene Meldrum's acid 107 to give intermediate 108 which undergoes cyclization with loss of acetone and carbon dioxide simultaneously to afford the desired compound 104.^66^

**Scheme 36:**
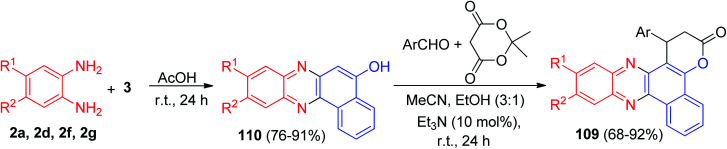
Synthesis of pyrano-phenazine derivatives 109.

Next, the pyrano-phenazine derivatives 109 were synthesized by an efficient procedure using the reaction between benzo[*a*]phenacin-5-ols with the condensation product of an aldehyde with Meldrum's acid in the presence of a catalytic amount of Et_3_N at ambient temperature. The first step consists in the condensation reaction between diamines and 3 in AcOH as solvent for 24 h to afford benzo[*a*]phenazin-5-ols 110 in 76–91% yields. The latter were used as C-nucleophiles to react with the condensation product of aromatic aldehyde with electron-donating and electron-withdrawing functional groups with Meldrum's acid in MeCN/EtOH (3 : 1) in the presence of Et_3_N (10 mol%) for 24 h to furnish the desired product 109 in 68–92% yields (Scheme 36).^67^

**Scheme 37:**
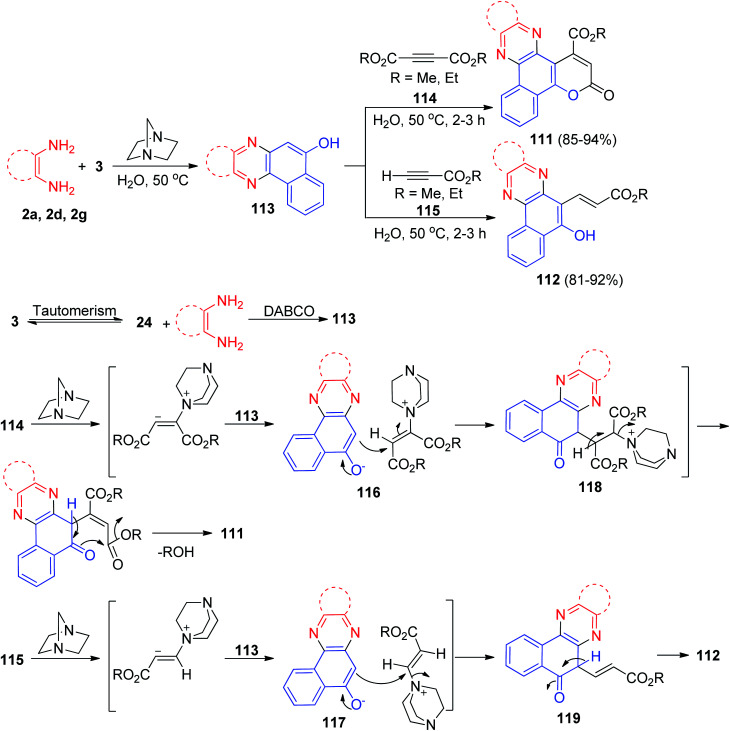
DABCO-catalyzed synthesis of 3-oxo-3*H*-benzo[*a*]pyrano[2,3-*c*]phenazine-1-carboxylate and 3-(5-hydroxybenzo[*a*]phenazin-6-yl)acrylate derivatives 111–112.

Further, Yazdani-Elah-Abadi and his co-workers reported an efficient and environmentally benign procedure for the synthesis of 3-oxo-3*H*-benzo[*a*]pyrano[2,3-*c*]phenazine-1-carboxylates 111 in 85–94% yields and 3-(5-hydroxybenzo[*a*]phenazin-6-yl)acrylate derivatives 112 in 81–92% yields has been developed by domino three-component condensation reaction between 3, benzene-1,2-diamines and acetylenic esters in the presence of a catalytic amount of DABCO as an expedient, eco-friendly and reusable base catalyst in water at 50 °C for 2–3 h. The suggested mechanism for the formation of the products is shown in Scheme 37. At first, 3 tautomerizes to intermediate 24. The primary condensation of 24 with benzene-1,2-diamine obtain benzo[*a*]phenazin-5-ol 113. Then, based on nucleophilicity of DABCO, the nucleophilic addition of DABCO to the acetylenic ester 114 or 115 and subsequent protonation in the presence of compound 113 gives intermediates 116 or 117, followed by attack of the anion on the cation part of intermediates 116 or 117 to form the intermediates 118 or 119. Intramolecular lactonization of the intermediate 118 leads to produce compound 111 and also, intermediate 119 followed by a tautomeric proton shift leads to the formation of desired product 112.^68^

**Scheme 38:**
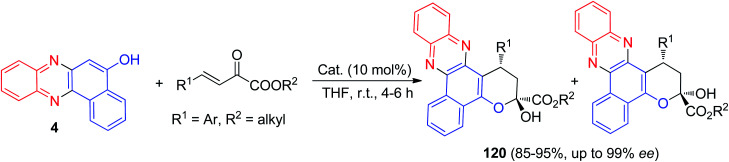
Synthesis of enantioselectively tetrahydropyran-fused benzo[*a*]phenazins 120.

In 2019, Kucherenko and co-workers reported synthesis of enantioselectively tetrahydropyran-fused benzo[*a*]phenazins 120 in 85–95% yields from β,γ-unsaturated α-keto esters and benzo[*a*]phenazin-5-ol (4) in the presence of bifunctional tertiary amine-squaramide catalyst in THF at room temperature for 4–6 h (Scheme 38).^69^

A one pot three-component reaction for the synthesis of benzo[*a*]pyrano-[2,3-*c*]phenazine derivatives 121 in 76–93% yields has been reported by Padmaja and co-workers. The synthesis was achieved by reacting benzo[*a*]phenazin-5-ol (4), aromatic aldehydes and (*E*)-*N*-methyl-1-(methylthio)-2-nitroethenamine at 120 °C under neat reaction conditions within 10 min. In addition, the synthesized products were screened for their *in vitro* anticancer properties.

**Scheme 39:**
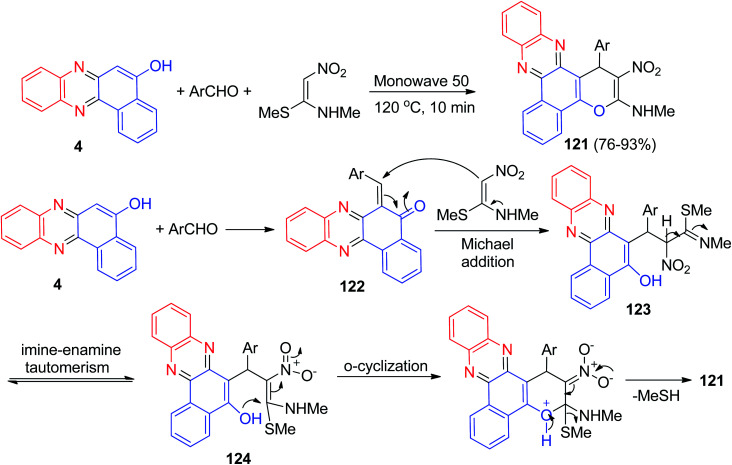
Synthesis of benzo[*a*]pyrano-[2,3-*c*]phenazine derivatives 121.

Some of these compounds displayed good antiproliferative activity against B16–F10 cells compared to the standard drug doxorubicin. A proposed mechanism for the formation of 121 is shown Scheme 39. At first, the condensation reaction of 4 and aromatic aldehyde affords adduct 122. Then the adduct 122 upon Michael-type addition with (*E*)-*N*-methyl-1-(methylthio)-2-nitroethenamine affords the open-chain intermediate 123. The intermediate 123 obviously tautomerized to another intermediate 124*via* an imine–enamine tautomerism. Finally, the intermediate 124 undergoes intramolecular O-cyclization to form 121 through the elimination of MeSH.^70^

**Scheme 40:**
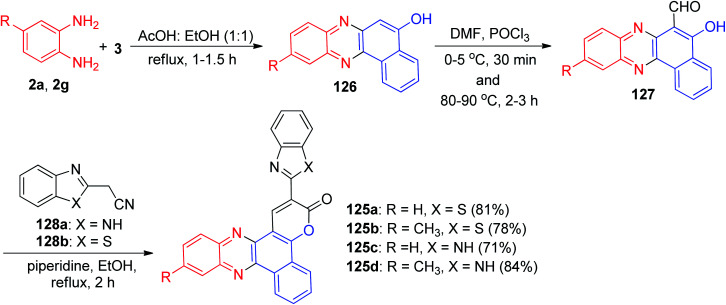
Synthesis of phenazine fused benzo coumarins 125.

The authors proposed synthesis of phenazine fused benzo coumarins 125a–d with negative solvatochromism and positive solvatochromic emission. The coumarin derivatives 125a–d were synthesized by following the sequence of reactions illustrated in Scheme 40. 2-Hydroxy-1,4-napthaquinone (3) was condensed with the substituted 1,2-diaminobenzenes in AcOH : EtOH (50 : 50) at 80 °C for 1–1.5 h to afford 126 in excellent yield. The electron rich 126 were subjected to formylation reaction under Vilsmeier–Haack conditions to obtain 5-hydroxybenzo[*a*]phenazine-6-carbaldehyde 127 in 55–59% yields. The condensation of 127 with the active methylene compounds 128a–b in refluxing EtOH in the presence of piperidine for 2 h under Knoevenagel conditions followed by an intramolecular cyclization gave 125 in 71–84% yields. Solutions of benzimidazole containing dyes (125c–d) in various solvents exhibited yellow to orange fluorescence while benzothiazole containing dyes (125a–b) showed brilliant bluish green fluorescence.^71^

A facile ruthenium(ii)-catalyzed regiospecific C–H/O–H oxidative annulation methodology was developed to construct isochromeno[8,1-*ab*]phenazines 129 in 39–80% yields by the reaction of benzo[*a*]phenazin-5-ols with alkynes in 1,2-DCE at reflux conditions for 12 h. The synthesized compounds showed prominent.

**Scheme 41:**
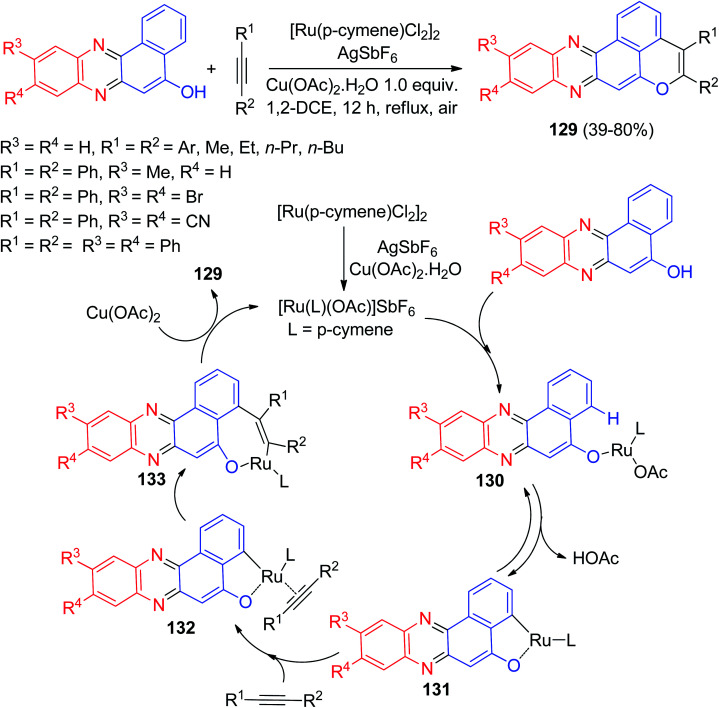
Synthesis of isochromeno[8,1-*ab*]phenazines 129.

FR fluorescence, with high quantum yield, and exhibited better cancer cell-imaging properties, with excellent biocompatibility. The plausible mechanism for the formation of 129 is shown in Scheme 41. The additive AgSbF_6_ likely initiates the catalytic reaction by dissociation of the dimeric form of the ruthenium complex [RuCl_2_(*p*-cymene)]_2_ and gives a reactive cationic ruthenium species. Then, the ruthenium complex gets attached with the directing group OH and forms the intermediate 130. Then, *peri*-C–H metalation of the intermediate 130 provides a five-membered ruthenacycle intermediate 131. Now, alkyne attaches with the intermediate 131 giving rise to intermediate 132. At this point, coordinative regioselective insertion of alkyne into the Ru–C bond of intermediate 132 gives intermediate 133. Finally, the ruthenium complex that is regenerated by Cu(OAc)_2_ forms the desired product 129.^72^

**Scheme 42:**
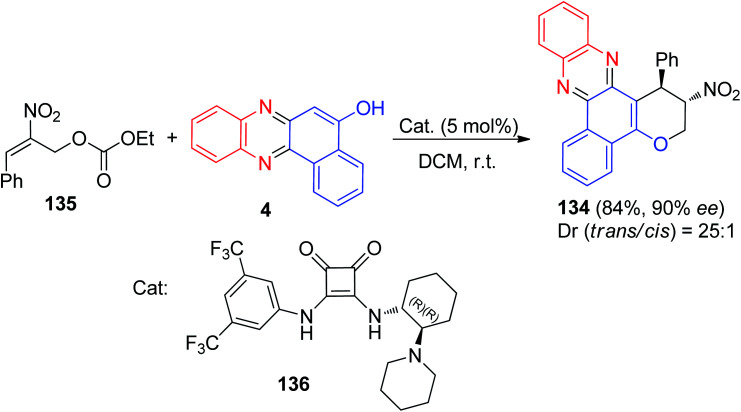
Enantioselective synthesis of 2-nitro-1-phenyl-2,3-dihydro-1*H*-benzo[*a*]pyrano[2,3-*c*]phenazine 134.

Recently, Kucherenko *et al.* reported highly stereo and enantioselective synthesis of 2-nitro-1-phenyl-2,3-dihydro-1*H*-benzo[*a*]pyrano[2,3-*c*]phenazine 134 (84% yield, 90% ee) by the reaction of 2-nitroallylic carbonate 135 with 4 in the presence of bifunctional Rawal-type tertiary amine 136 (5 mol%) in DCM at room temperature (Scheme 42).^73^

### 2.2 Synthesis of spirobenzopyranophenazines

In 2013, Mahdavinia and his co-workers described an efficient regio- and chemoselective method for the synthesis of 3-amino-2′-oxospiro[benzo[*c*]pyrano[3,2-*a*]phenazine-1,3′-indoline]-2-carbonitrile/carboxylate derivatives 137 in 95–100% yields *via* the one-pot, two-step four-component domino coupling of 3, benzene-1,2-diamines, isatins, and malononitrile/cyanoacetic ester in the presence of DABCO in EtOH under reflux conditions for 10–15 min.

**Scheme 43:**
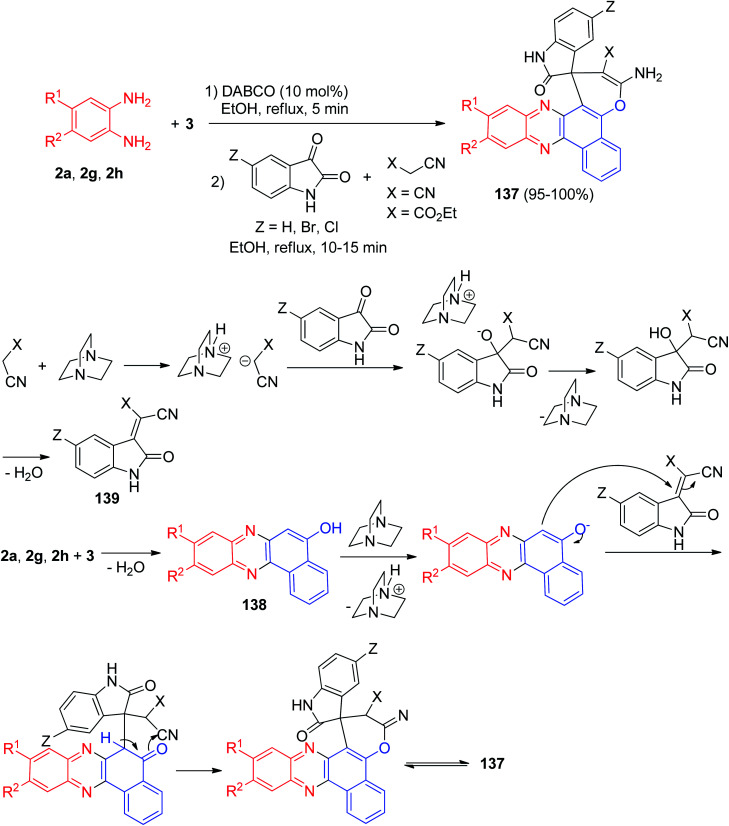
Synthesis of 3-amino-2′-oxospiro[benzo[*c*]pyrano[3,2-*a*]phenazine-1,3′-indoline]-2-carbonitrile/carboxylate derivatives 137.

A plausible mechanism for the formation of 137 is shown in Scheme 43. 2-Hydroxynaphthalene-1,4-dione (3) is converted into the corresponding benzo[*a*]phenazin-5-ol 138 on reaction with benzene-1,2-diamines; isatin can be easily attacked by the carbon nucleophilic center of malononitrile leading to Knoevenagel condensation products (intermediate 139) finally; the Knoevenagel products attacked by 138 leading to the desired product 137.^74^

**Scheme 44:**
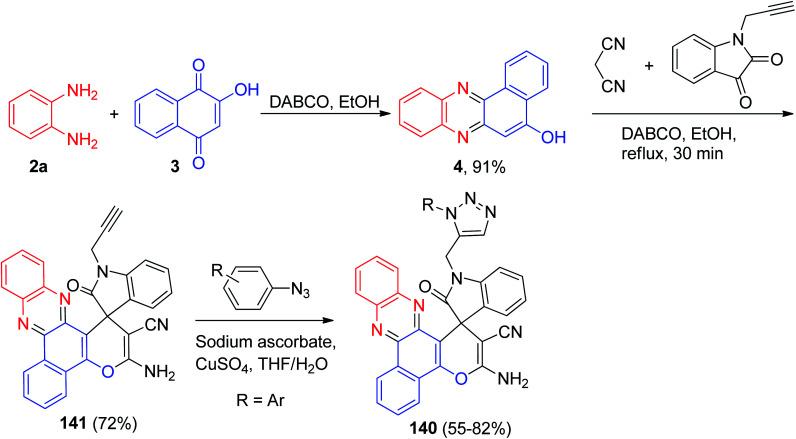
Preparation of benzo[*a*]-phenazine derivatives 140.

In 2017, a series of benzo[*a*]-phenazine derivatives 140 as hybrid molecules of phenazine, pyran, indole and 1,2,3-triazole pharmacophores were constructed in 55–82% yields. Firstly, the reaction of 3, 2a, malononitrile and 1-(prop-2-yn-1-yl)indoline-2,3-dione in the presence of DABCO in refluxing EtOH for 30 min afforded the desired compound 141 in 72% yield. Finally, target compounds were synthesized using compound 141, and aromatic azide in the presence of sodium ascorbate and CuSO_4_ in THF/H_2_O (Scheme 44). Cytotoxic evaluation indicated that some compounds exhibited moderate cytotoxicity against HCT116, MCF7, HepG2 and A549 cancer cell lines *in vitro*. Moreover, all compounds had low or no effect against L02 and HUVEC non-cancer cell lines.^75^

**Scheme 45:**
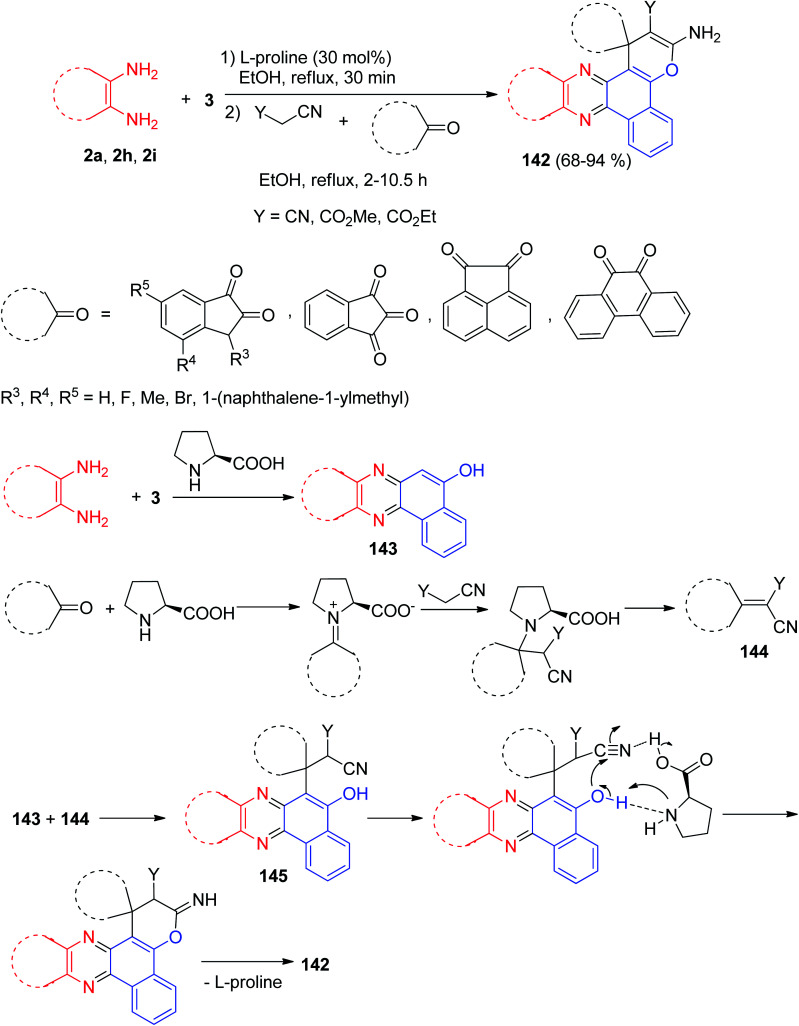
Synthesis of spiro[benzo[*c*]pyrano[3,2-*a*]phenazines] 142.

In addition, Hasaninejad *et al.* reported synthesis of polyfunctionalized spiro[benzo[*c*]pyrano[3,2-*a*]phenazine] derivatives 142 in 68–94% yields by the one-pot two-step condensation of 3 with aromatic 1,2-diamines to form the corresponding quinoxalines and then cyclo-condensation with an alkylmalonate and a cyclic carbonyl compound in EtOH under reflux conditions in the presence of l-proline as a bifunctional catalyst leads to the corresponding products 142 in high yields (68–94%) and short reaction times (2–10.5 h). The proposed mechanism for the synthesis of 142 is shown in Scheme 45. Initially, 3 and the benzene-1,2-diamine react to form the corresponding quinoxalinone 143. Knoevenagel condensation of cyclic ketones with malono derivative affords an intermediate 144, which undergoes Michael addition with 143 to form intermediate 145. The enolate O-atom of the formed intermediate 145 attacks the CN group, and a subsequent H-atom shift leads to compound 142.^76^

**Scheme 46:**
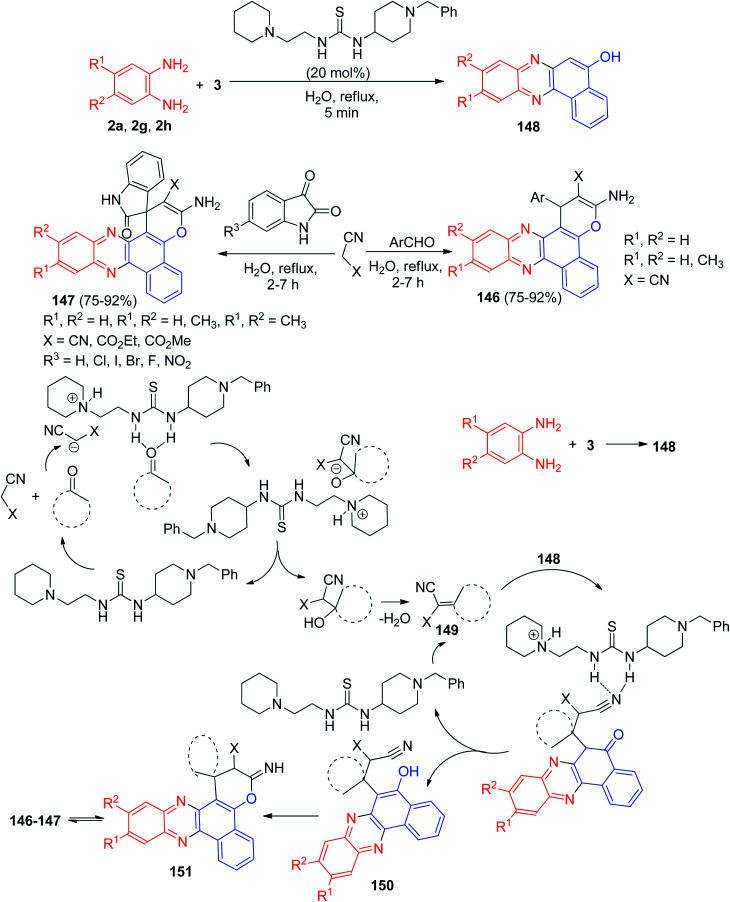
Synthesis of pyrano-fused benzophenazines 146–147.

Further, a series of pyrano-fused benzophenazines 146–147 in 75–92% yields were synthesized using a bifunctional thiourea-based organocatalyst from the one-pot, two-step four-component reaction of 3, benzene-1,2-diamines, malononitrile or its derivatives and isatins or aromatic aldehydes in water under reflux conditions for 2–7 h. The proposed mechanism is outlined in Scheme 46. They believe that the condensation reaction between 3 and the benzene-1,2-diamine leading to the corresponding benzo[*a*]phenazin-5-ol 148 does not need any catalyst. However, organocatalyst plays significant role in other steps, and it activates both the electrophile and nucleophile through its thiourea moiety and basic amine moiety, respectively. The Knoevenagel condensation of isatin or aldehyde with malononitrile affords 149, which undergoes a Michael addition with 148 to form intermediate 150 in the presence of organocatalyst. A subsequent cyclization leads to the formation of 151 which undergoes tautomerization to form the corresponding final products 146–147.^77^

**Scheme 47:**
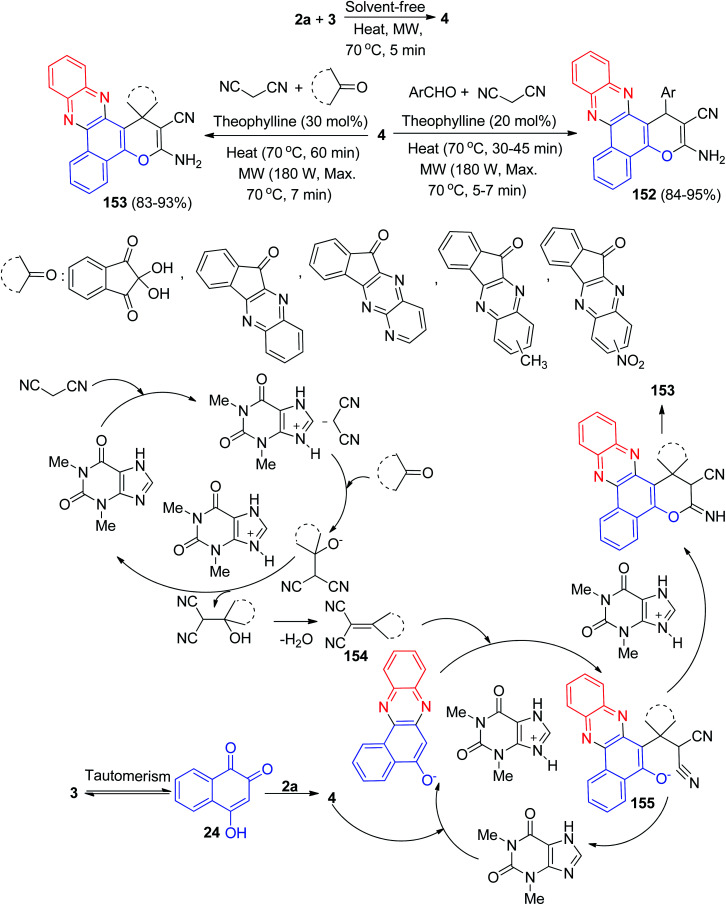
Theophylline catalyzed synthesis of benzo[*a*]pyrano[2,3-*c*]phenazines 152 and spiro[benzo[*a*]pyrano[2,3-*c*]phenazine] derivatives 153.

In addition, Maghsoodlou *et al.* have demonstrated a green one-pot procedure for the synthesis of benzo[*a*]pyrano[2,3-*c*]phenazine derivatives 152–153 in 83–95% yields by domino multi-component condensation reaction between 3, 2a, malononitrile and cyclic ketones or aromatic aldehydes in the presence of a catalytic amount of 1,3-dimethyl-7*H*-purine-2,6-dione (theophylline) as are usable solid base catalyst under thermal (70 °C), microwave irradiation (180 W, max. 70 °C) and solvent-free conditions. The suggested mechanism for the formation of the products is shown in Scheme 47. On the basis of this mechanism, at first, 3 tautomerizes to intermediate 24. The primary condensation of 24 with 2a obtains 4. On this mechanism, theophylline is an efficient catalyst to form the olefin 154, which readily prepares *in situ* from Knoevenagel condensation of carbonyl groups of aldehyde or cyclic ketones with malononitrile. The Michael addition of 4 with olefin 154 in the presence of theophylline finally give intermediate 155, which then makes the inner molecular ring to be formed after a tautomeric proton shift to produce benzo[*a*]pyrano[2,3-*c*]phenazines 152 and spiro[benzo[*a*]pyrano[2,3-c] phenazine] derivatives 153.^78^

**Scheme 48:**
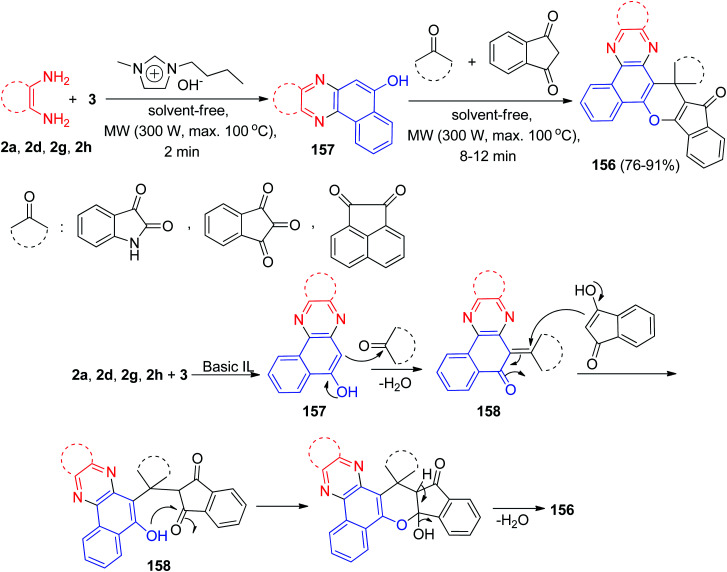
MW-assisted synthesis of spiro-benzo[*a*]phenazines 156 in the presence of [BMIM]OH.

Later, a green strategy for the synthesis of a biologically and pharmaceutically interesting multi-functionalized diverse spiro-benzo[*a*]phenazine annulated heterocycles 156 in 76–91% yields by one-pot, two-step domino reaction starting from 3, benzene-1,2-diamines, a cyclic carbonyl compound, and 1,3-indandione in the presence of a basic ionic liquid (1-butyl-3-methylimidazolium hydroxide: [BMIM]OH) as a reusable catalyst with the assistance of microwave irradiation (300 W) under solvent-free conditions at 100 °C for 8–12 min. The probable mechanism is given in Scheme 48. On the basis of this suggested mechanism, the primary condensation of 3 with benzene-1,2-diamines in the presence of [BMIM]OH gives benzo[*a*]phenazin-5-ols 157. Then, the Knoevenagel condensation between 157 and cyclic ketones produce adduct 158, which act as a Michael acceptor. The 1,3-indandione attacks the Knoevenagel adduct 158 in a Michael-type addition to produce the intermediate 159, which then makes the inner molecular ring to be formed after a tautomeric proton shift to generate 156.^79^

**Scheme 49:**
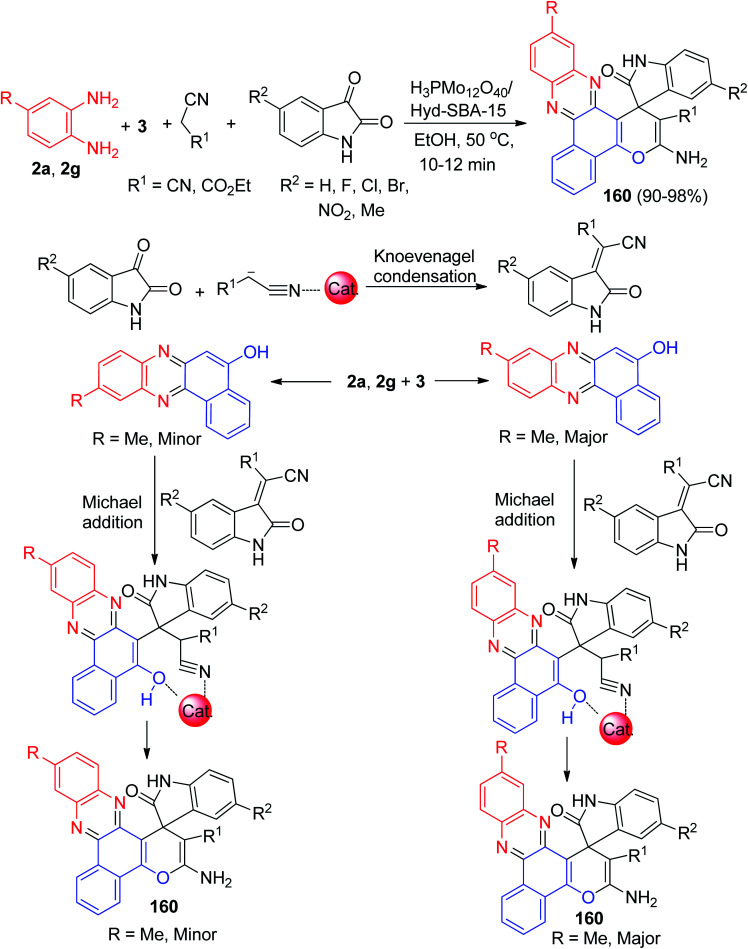
Synthesis of 3-amino-2′-oxospiro[benzo[*c*]pyrano[3,2-*a*]phenazine-1,3′-indoline]-2-carbonitrile/carboxylate derivatives 160 catalyzed by H_3_PMo_12_O_40_/Hyd-SBA-15.

After that, for synthesis of 3-amino-2′-oxospiro[benzo[*c*]pyrano[3,2-a]phenazine-1,3′-indoline]-2-carbonitrile/carboxylate derivatives 160 in 90–98% yields, Safaei-Ghomi and Bakhtiari developed a domino coupling reaction involving 3, benzene-1,2-diamines, malone derivatives and isatin derivatives catalyzed by H_3_PMo_12_O_40_/Hyd-SBA-15 in EtOH at 50 °C for 10–12 min. A mechanism for a plausible catalytic cycle for this domino MCR is outlined in Scheme 49. Coordination of the carbonyl groups of 3 to a molybdenum sites at the surface of H_3_PMo_12_O_40_/Hyd-SBA-15 would increase the activity. On the other side, the electrophilicity of malono derivatives increased by coordination with the H_3_PMo_12_O_40_/Hyd-SBA-15. Molybdenum sites at the surface of H_3_PMo_12_O_40_/Hyd-SBA-15 increased the electrophilicity of reactants, which simplifies the reaction. Moreover, in the presence of monosubstituted benzene-1,2-diamine, major and minor isomers of the corresponding products are generated.^80^

Next, Kumar *et al.* described a domino protocol for the synthesis of structurally diverse spiroannulated pyrimidophenazines 161 in 89–96% yields involving a four-component reaction of 3, 2a, cyclic ketones and amino derivatives in the presence of erbium doped.

**Scheme 50:**
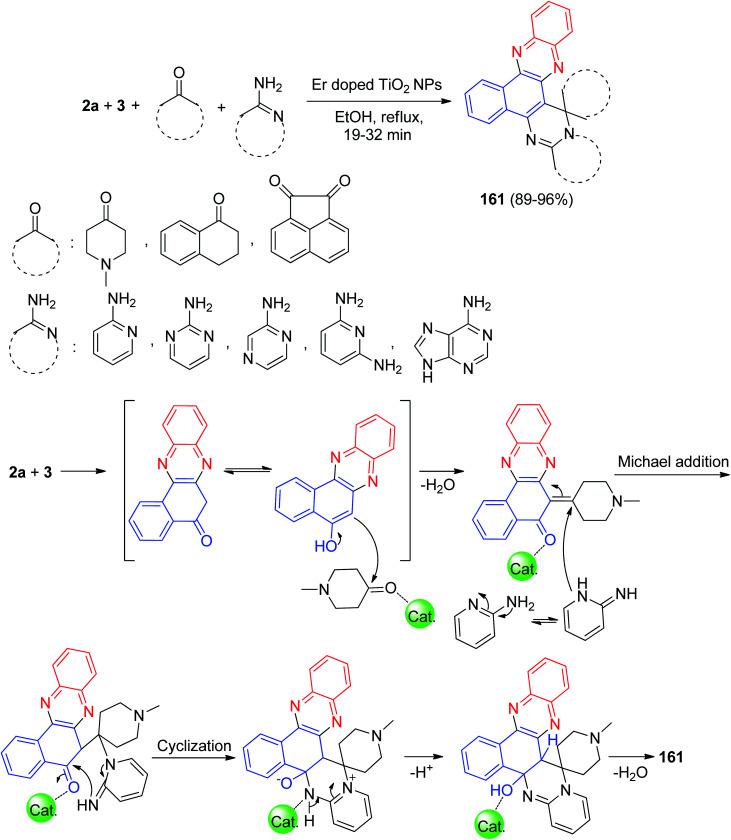
Synthesis of spiroannulated pyrimidophenazines 161.

TiO_2_ nanoparticles as a recyclable and reusable heterogeneous acid catalyst in EtOH under reflux conditions for 19–32 min. The mechanism of the reaction proceeds with the following steps involving the Michael addition, cyclization and dehydration as presented in Scheme 50. The doping of erbium with TiO_2_ NPs increased the efficiency of the resulting catalyst and thus facilitated the reaction in better way as compared with TiO_2_ NPs.^81^

**Scheme 51:**
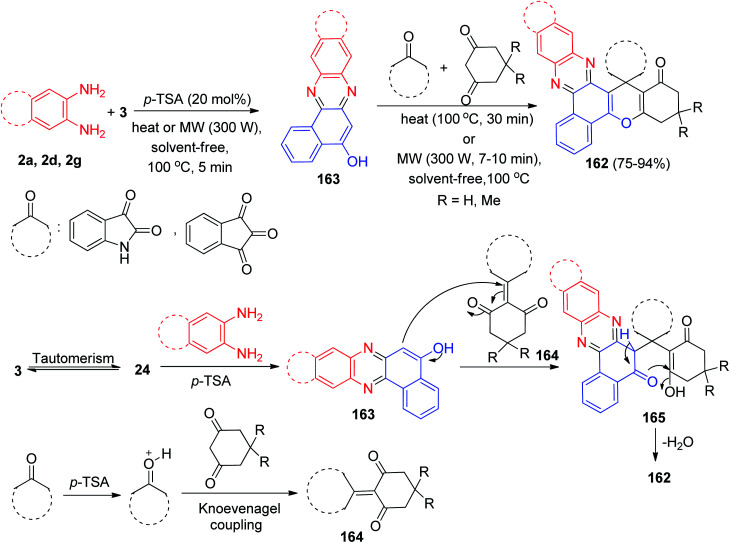
Synthesis of novel spiro[benzo[*a*]chromeno[2,3-*c*]phenazine] derivatives 162 in the presence of *p*-TSA.

In 2019, *p*-toluenesulfonic acid was applied as an efficient and solid acid catalyst for the one-pot, four-component condensation between 3, benzene-1,2-diamines, cyclic 1,3-dicarbonyl compounds and isatin or ninhydrin to afford the corresponding spiro[benzo[*a*]chromeno[2,3-*c*]phenazine] derivatives 162 in 75–94% yields *via* a new two-step domino protocol under conventional heating (100 °C, 30 min) and microwave irradiation (300 W, 100 °C, 7–10 min) under solvent-free conditions. The probable mechanism for the domino synthesis of 162 using *p*-TSA is given in Scheme 51. Initially, 3 tautomerizes to intermediate 24. The early condensation of 24 with benzene-1,2-diamines obtain benzo[*a*]phenazin-5-ol 163. Then, the Knoevenagel condensation between the dimedone and cyclic ketone to produce adduct 164, which acts as a Michael acceptor. The enol 163 attacks Knoevenagel adduct 164 in a Michael-type addition to produce intermediate 165 which then makes the inner molecular ring to be formed after a tautomeric proton shift to generate 162.^82^

### 2.3 Synthesis of benzo[*a*]benzochromeno phenazine

**Scheme 52:**
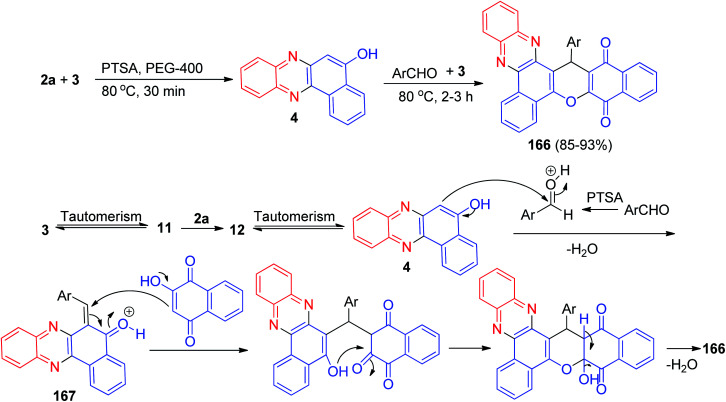
*p*-TSA catalyzed synthesis of 11*H*-benzo[*a*]benzo[6,7]chromeno[2,3-*c*]phenazine-11,16(17*H*)-diones 166.

In 2016, an efficient *p*-toluenesulfonic acid catalyzed synthesis of 11*H*-benzo[*a*]benzo[6,7]chromeno[2,3-*c*]phenazine-11,16(17*H*)-dione derivatives 166 in 85–93% yields has been described by one-pot, two-step four-component condensation of 3, 2a, aromatic aldehydes using polyethylene glycol as solvent at 80 °C for 2–3 h. The plausible mechanism of this domino reaction is depicted in Scheme 52. Initially, 3 tautomerizes to intermediate 11. The primary condensation of 11 with 2a obtains 6*H*-benzo[*a*]phenazin-5-one (12), which in tautomerism equilibrium prepares 4. The catalyst *p*-TSA appears to play a key role as acid in the reaction to form (6-benzylidenebenzo[*a*]phenazin-5(6*H*)-ylidene)oxonium 167, which prepares *in situ* from condensation of aldehyde with 4. Subsequent Michael addition of 3 with 167, followed by cyclization and dehydration, leads to the formation of the desired product 166.^83^

**Scheme 53:**
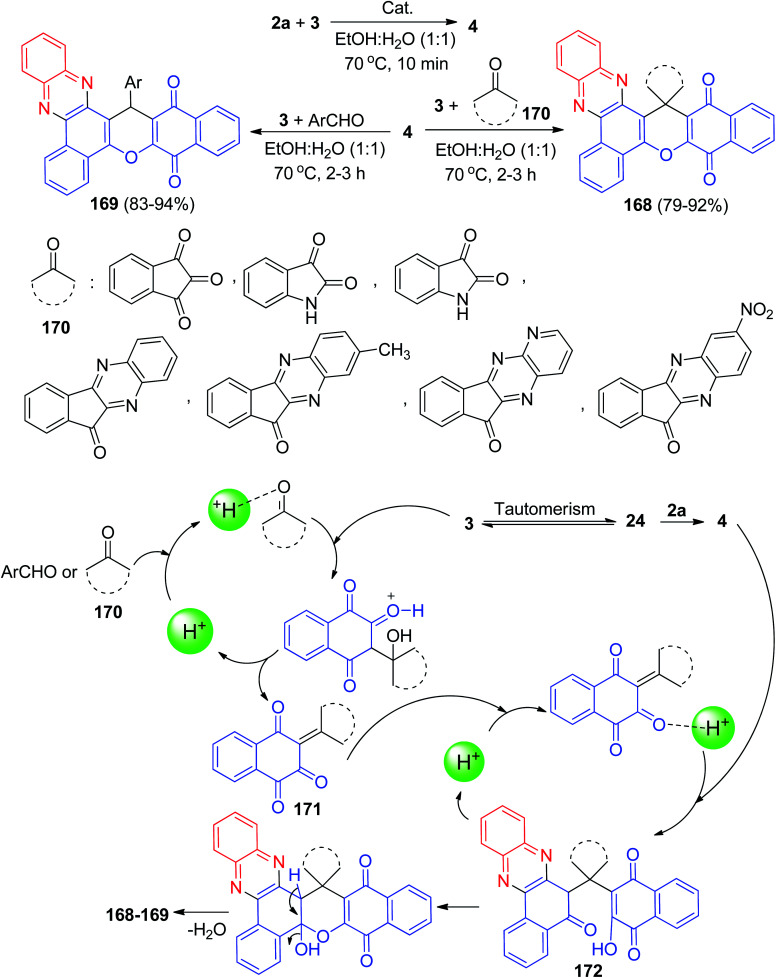
Thioglycolic acid catalyzed synthesis of benzo[*a*]benzo[6,7]chromeno[2,3-*c*]phenazine derivatives 168–169.

Next, Yazdani-Elah-Abadi *et al.* reported superparamagnetic nanoparticles of modified thioglycolic acid (γ-Fe_2_O_3_@SiO_2_–SCH_2_CO_2_H) as a green catalyst for the one-pot synthesis of spiro[benzo[*a*]benzo[6,7]chromeno[2,3-*c*]phenazine] derivatives 168 in 79–92% yields and benzo[*a*]benzo[6,7]chromeno[2,3-*c*]phenazine 169 in 83–94% yields *via* domino Knoevenagel–Michael–cyclization reaction of 3, 2a and ninhydrin or isatin or cyclic ketones 170 or aromatic aldehydes in EtOH : H_2_O (1 : 1) at 70 °C for 2–3 h. This magnetic organocatalyst was easily isolated from the reaction mixture by magnetic decantation using an external magnet. The suggested mechanism for the formation of the products is shown in Scheme 53. On the basis of this mechanism, at first, 3 tautomerizes to intermediate 24. The primary condensation of 24 with 2a produces 4. With this mechanism, MNPs-thioglycolic acid is an efficient catalyst for forming the olefin 171, which is readily prepared *in situ* from Knoevenagel condensation of carbonyl groups of aldehyde or cyclic ketones 170 with 3. The Michael addition of 4 with olefin 171 in the presence of MNPs-thioglycolic acid finally gives intermediate 172, which then makes the inner molecular ring to be formed after a tautomeric proton shift to produce the target products 168–169.^84^

**Scheme 54:**
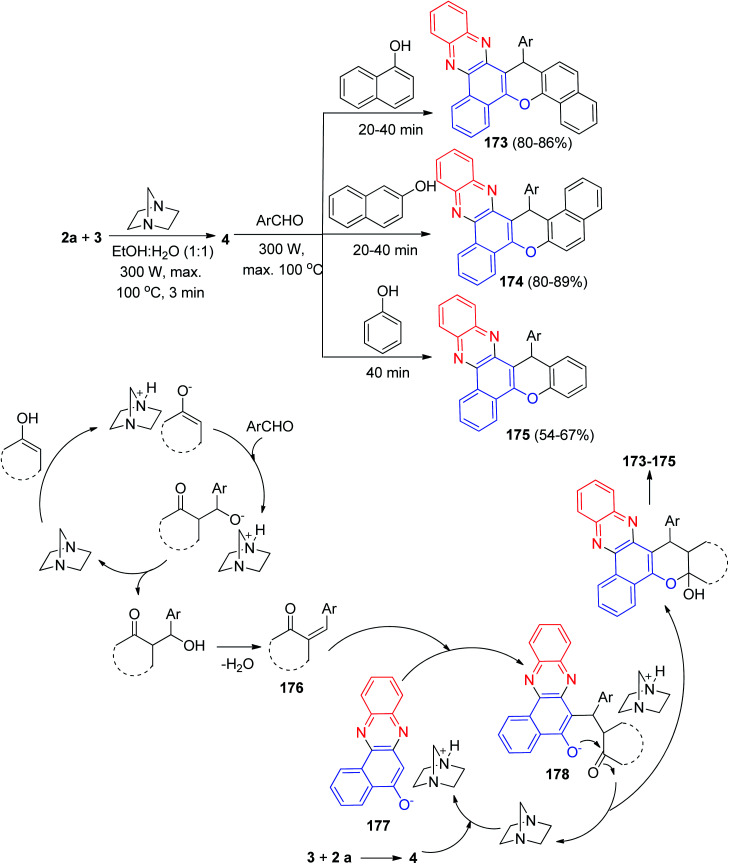
DABCO catalyzed synthesis of benzo[*a*]chromeno[2,3-*c*]phenazine derivatives 173–175.

After that, Yazdani-Elah-Abadi and his co-workers described the preparation of benzo[*a*]chromeno[2,3-*c*]phenazine derivatives 173–175 in 54–89% yields by domino four-component condensation reaction between 3, 2a, aromatic aldehydes, and naphthols or phenol in the presence of a catalytic amount of DABCO (20 mol%) as a reusable base catalyst under microwave irradiation (at 300 W and max. 100 °C) in EtOH/H_2_O (1 : 1) within 20–40 min. The probable mechanism is outlined in Scheme 54. On the basis of this mechanism, the primary condensation of 3 with 2a in the presence of DABCO gives 4. Based on this mechanism, DABCO is an efficient catalyst to form the olefin 176, which is readily prepared *in situ* from the Knoevenagel condensation of aromatic aldehyde with naphthols. In the presence of DABCO, 4 converts to its corresponding enolate form 177, to be able to react (Michael addition) easily with 176 and to eventually give rise to the formation of intermediate 178, which then makes the inner molecular ring be formed after a tautomeric proton shift to produce the desired products 173–175.^85^

### 2.4 Synthesis of benzopyridophenazines

**Scheme 55:**
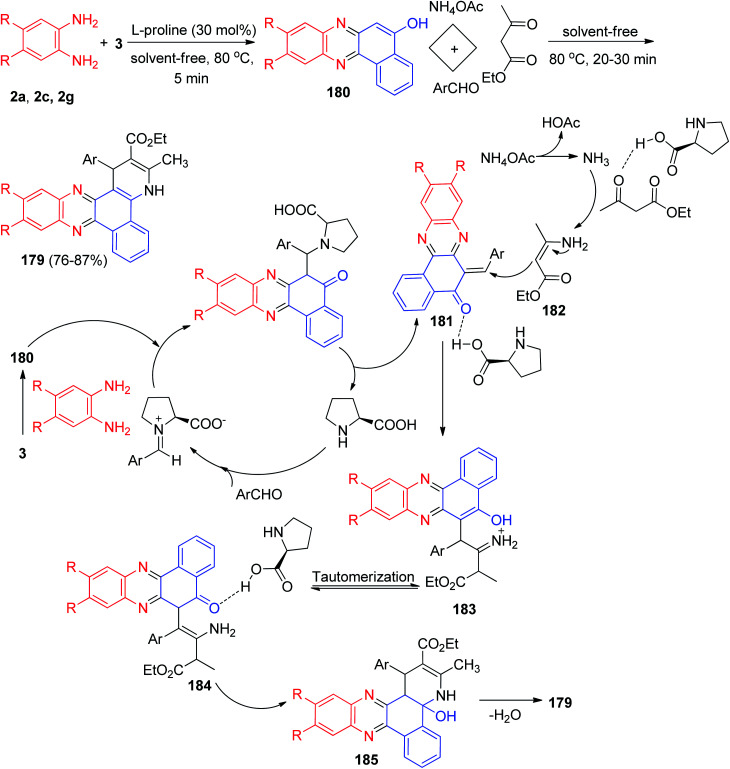
l-Proline catalyzed synthesis of 1,4-dihydrobenzo[*a*]pyrido[2,3-*c*]phenazines 179.

In 2017, l-proline has been used as a reusable and bifunctional organocatalyst for the one-pot, two-step, five-component synthesis of 1,4-dihydrobenzo[*a*]pyrido[2,3-*c*]phenazines 179 in 76–87% yields by the condensation reaction of 3, aromatic 1,2-diamines, aldehydes, ammonium acetate and ethyl acetoacetate under conventional heating in solvent-free conditions at 80 °C for 20–30 min. The probable mechanism is outlined in Scheme 55. On the basis of this mechanism, the primary condensation of 3 with benzene-1,2-diamines in the presence of l-proline gives benzo[*a*]phenazin-5-ol 180. On this mechanism, l-proline is an efficient catalyst to form the olefin 181, which readily prepares *in situ* from Knoevenagel condensation of aromatic aldehyde with 180. On the other hand, NH_3_ resulting from ammonium acetate and ethyl acetoacetate by l-proline yields enamine 182. Subsequently, the reaction between olefin 181 and enamine 182 gives the corresponding intermediate 183. Tautomerization of intermediate 183 affords intermediate 184 which suffer an intramolecular nucleophilic attack of the NH_2_ group to the activated carbonyl group providing intermediate 185. At the end, dehydration of this intermediate yields the desired target molecule 179.^86^

**Scheme 56:**
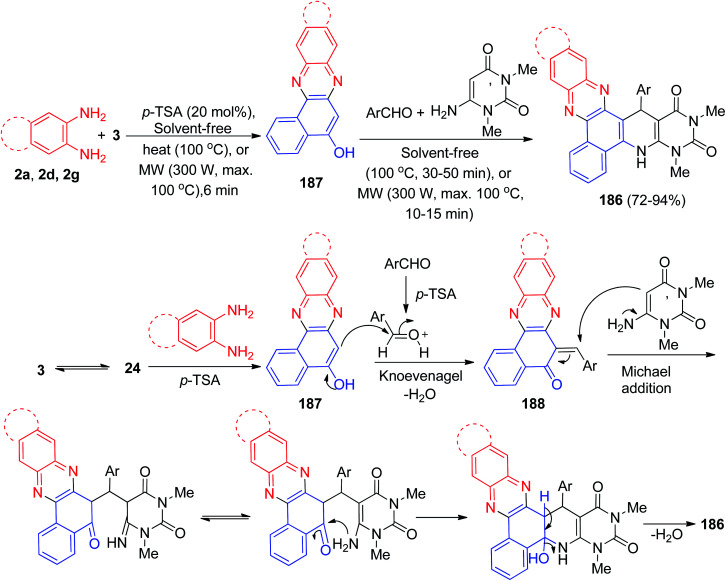
*p*-TSA catalyzed synthesis of pyrimido-fused benzophenazines 186.

After that, Mohebat *et al.* synthesized polyfunctionalized benzo[*a*]pyrimido[5′,4′:5,6]pyrido[2,3-*c*]phenazine derivatives 186 in 72–94% yields by a one-pot, four-component sequential reaction between 3, benzene-1,2-diamines, benzaldehydes, and 6-amino-1,3-dimethyluracil in the presence of *p*-TSA as solid acid catalyst under solvent-free microwave irradiation (300 W for 100 °C, 10–15 min) or conventional heating conditions at 100 °C for 30–50 min. A suggested mechanism is proposed in Scheme 56. First, the organization of benzo[*a*]phenazin-5-ol (187) can be explained *via* a condensation of 3 and benzene-1,2-diamines. Then the efficient Knoevenagel condensation of 187 and aryladehyde created product 188. Lastly, compound 186 was offered by a sequence of facile Michael addition/cyclization/dehydration reactions between 188 and 6-amino-1,3-dimethyluracil.^87^

**Scheme 57:**
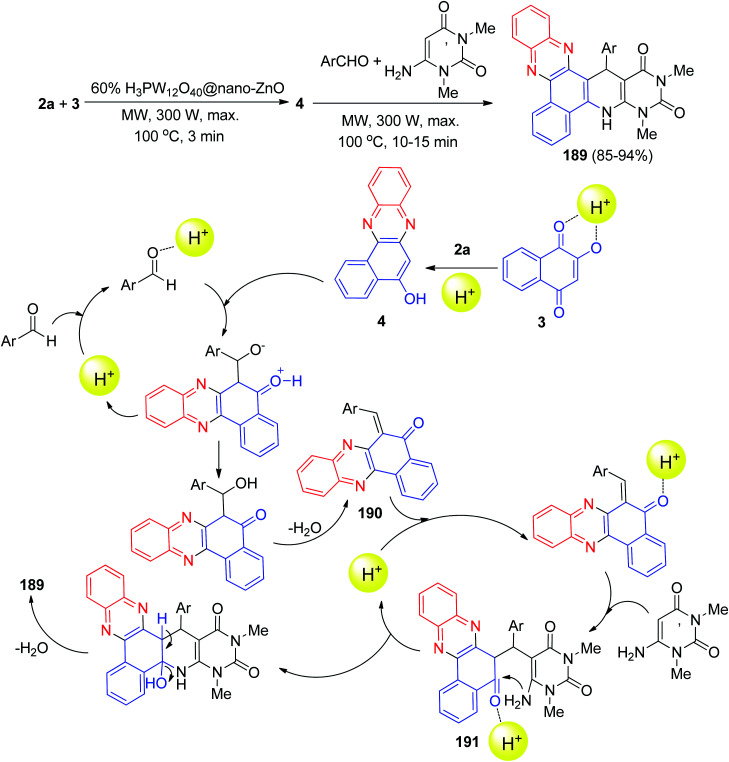
H_3_PW_12_O_40_@nano-ZnO-catalyzed synthesis of pyrimido-fused benzophenazines 189.

Next, an environmentally benign procedure for the synthesis of heteroaryl-substituted dihydrobenzo[*a*]pyrimido[5′,4′:5,6]pyrido[2,3-*c*]phenazines 189 in 85–94% yields has been developed *via* condensation/Knoevenagel/Michael/heterocyclization reactions of 3, 2a, aromatic aldehydes, and 6-amino-1,3-dimethyluracil in the presence of H_3_PW_12_O_40_@nano-ZnO as a recyclable heterogeneous catalyst in aqueous medium under microwave irradiation (300 W, max. 100 °C) for 10–15 min. A detailed reaction mechanism is outlined in Scheme 57. On the basis of this mechanism, the primary condensation of 3 with 2a in the presence of H_3_PW_12_O_40_@nano-ZnO gives 4. On the other hand, the catalyst to form the olefin 190, which readily prepares *in situ* from Knoevenagel condensation of aromatic aldehyde with 4. The Michael addition of 6-amino-1,3-dimethyluracil with olefin 190 in the presence of the catalyst finally give intermediate 191, which then makes the inner molecular ring to be formed after a tautomeric proton shift to produce the corresponding product 189.^88^

**Scheme 58:**
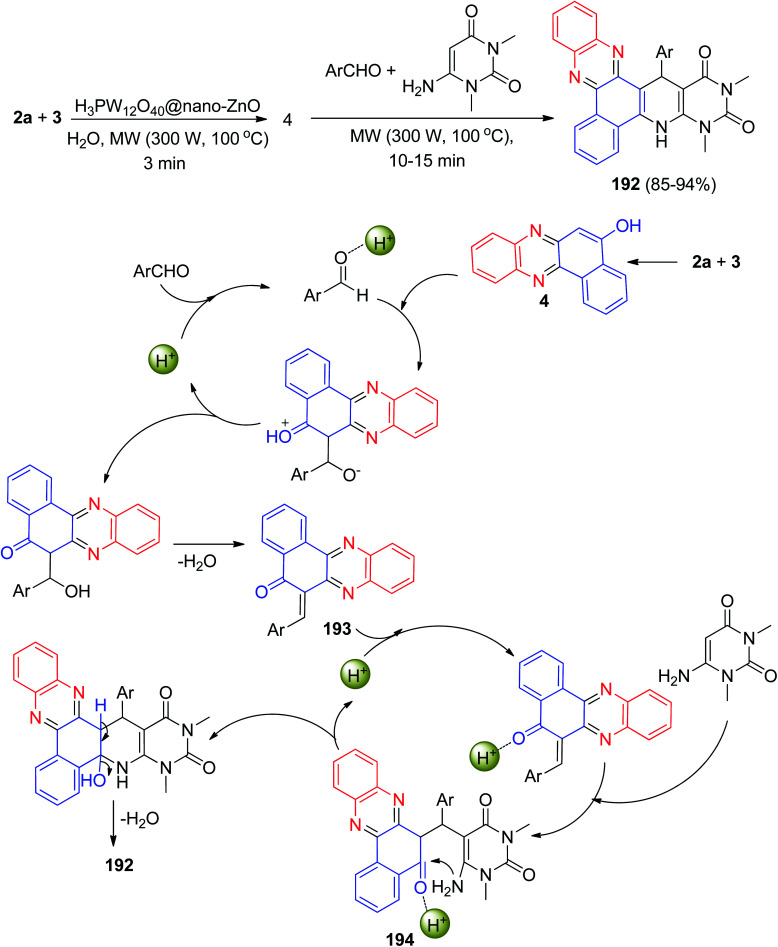
Synthesis of heteroaryl-substituted dihydrobenzo[*a*]pyrimido[5′,4′:5,6]pyrido[2,3-*c*]phenazines 192.

In 2020, an environmentally benign procedure for the synthesis of heteroaryl-substituted dihydrobenzo[*a*]pyrimido[5′,4′:5,6]pyrido[2,3-*c*]phenazines 192 in 85–94% yields has been developed *via* condensation/Knoevenagel/Michael/heterocyclization reactions of 3, 2a, aromatic aldehydes, and 6-amino-1,3-dimethyluracil in the presence of H_3_PW_12_O_40_@nano-ZnO as a recyclable heterogeneous catalyst in aqueous medium under microwave irradiation (300 W, max. 100 °C) for 10–15 min. A detailed reaction mechanism is outlined in Scheme 58. On the basis of this mechanism, the primary condensation of 3 with 2a in the presence of the catalyst gives 4. On the other hand, H_3_PW_12_O_40_@nano-ZnO is an efficient catalyst to form the olefin 193, which readily prepares *in situ* from Knoevenagel condensation of aromatic aldehyde with 4. The Michael addition of 6-amino-1,3-dimethyluracil with olefin 193 in the presence of the catalyst finally give intermediate 194, which then makes the inner molecular ring to be formed after a tautomeric proton shift to produce the corresponding product 192.^89^

### 2.5 Synthesis of benzofurophenazines

**Scheme 59:**
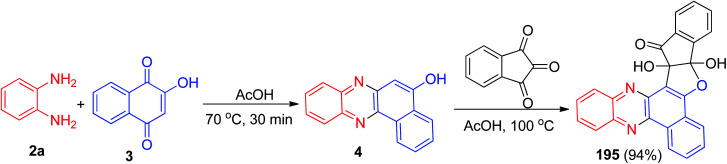
Synthesis of indeno-furan derivative 195.

In 2015, pH on–off fluorescent chemosensors based on indeno-furan derivative 195 has been synthesized. The synthesis of the compound was achieved firstly synthesize benzo[*a*]phenazine-5-ol (4) *via* condensation of 3 and 2a in glacial acetic acid at 70 °C for 30 min. Then, the reaction of ninhydrin with 4 catalyzed by glacial acetic acid at 100 °C led to the formation of desired compound 195 in 94% yield (Scheme 59). The change in fluorescence of 195 is reversible within the wide pH range of 2 to 11. The color change of this sensor could also be detected by naked eyes thus making them promising candidate as colorimetric pH indicator also.^90^

**Scheme 60:**
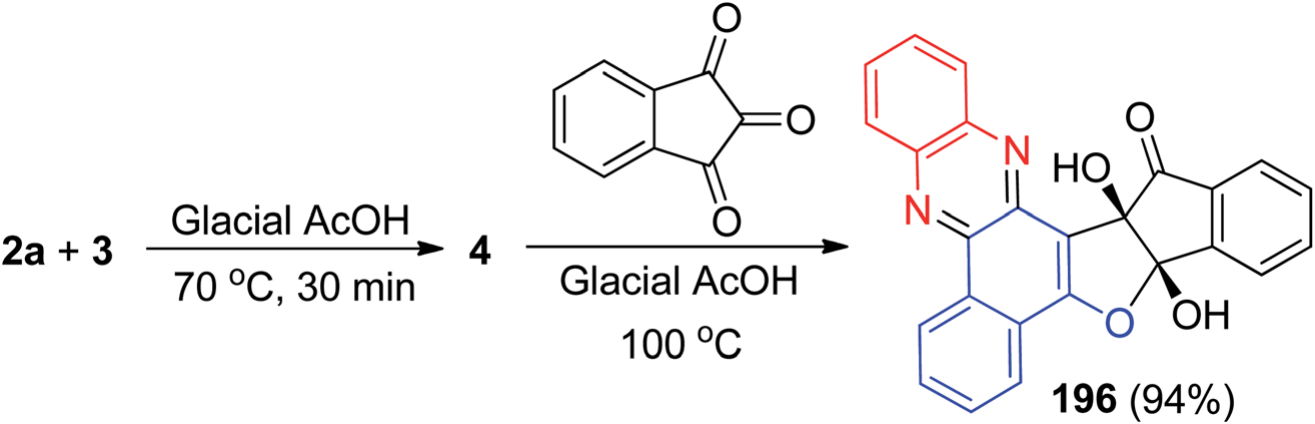
Synthesis of 10a,15a-dihydroxy-10a-*H*-benzo[*a*]indeno[2′,1′:4,5]furo[2,3-*c*]phenazin-15(15a*H*)-one 196.

Next, Khurana *et al.* have prepared 10a,15a-dihydroxy-10a-*H*-benzo[*a*]indeno[2′,1′:4,5]furo[2,3-*c*]phenazin-15(15a*H*)-one 196 in 94% yield as a fluorescent sensor *via* a one-pot, two-step procedure from a three-component condensation reaction of 3, 2a and ninhydrin in glacial acetic acid (Scheme 60). Compound 196 exhibits high sensitivity and selectivity towards Cu^2+^ and Pb^2+^ ions over other metal ions by fluorescence quenching. Moreover, this sensor exhibited a visible color change from light orange to pink, and yellow in the presence of Cu^2+^ and Pb^2+^, respectively.^91^

**Scheme 61:**
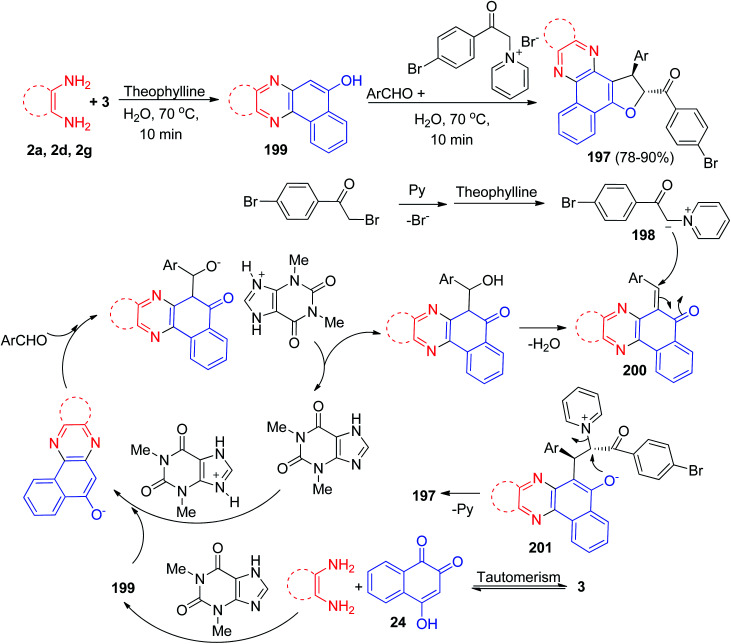
Synthesis of 1,2-dihydrobenzo[*a*]furo[2,3-*c*]phenazines 197 by using theophylline.

After that, Mohebat *et al.* reported a one-pot, two-step procedure for the synthesis of 1,2-dihydrobenzo[*a*]furo[2,3-*c*]phenazine derivatives 197 in 78–90% yields with high diastereoselectivity by condensation reaction between 3, benzene-1,2-diamines, aromatic aldehyde and pyridinium ylide 198 in the presence of a catalytic amount of theophylline (20 mol%) as an expedient, eco-friendly and reusable solid base catalyst in water at 70 °C for 3–4 h. The suggested mechanism is depicted in Scheme 61. At first, 3 tautomerizes to intermediate 24. The primary condensation of 24 with benzene-1,2-diamine obtain 199. On this mechanism, theophylline is an efficient catalyst to form the 6-benzylidenebenzo[*a*]phenazin-5(6*H*)-one 200, which easily prepares *in situ* from Knoevenagel condensation of 199 with carbonyl group of aldehyde. On the other hand, the pyridinium ylide 198, which forms from the reaction of 1-(2-(4-bromophenyl)-2-oxoethyl)pyridinium with theophylline undergoes Michael addition to intermediate 200 to afford the enolate intermediate 201. Eventually, the enolate 201 eliminates pyridine (intramolecular S_N_2) and cyclizes instantly to produce 197.^92^

**Scheme 62:**
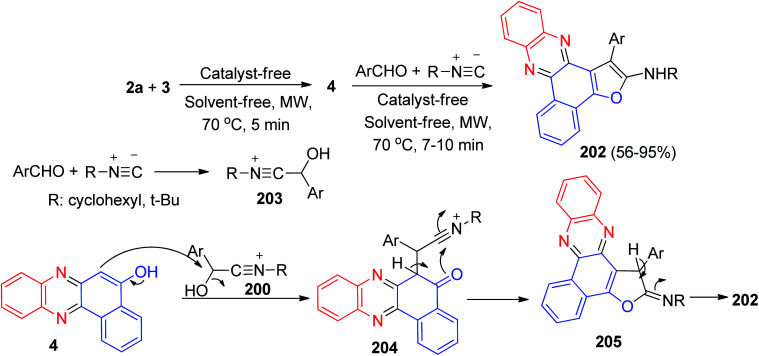
Synthesis of benzo[*a*]furo[2,3-*c*]phenazines 202.

In addition, Mohebat *et al.* developed an efficient protocol for the one-pot four-component synthesis of benzo[*a*]furo[2,3-*c*]phenazines 202 in 56–95% yields starting from the reaction of 3, 2a, isocyanide and aromatic aldehydes under catalyst- and solvent-free microwave conditions (180 W) at 70 °C for 7–10 min. The mechanism of these reactions is plausibly based on the key intermediate 4 of 3 and 2a, as analyzed from the experimental results. On the other side, condensation of alkyl isocyanides with aryl aldehydes afforded intermediate 203. In the following, intermediate 4 attacks intermediate 203 to give the formed intermediate 204, which in subsequent cyclization formed intermediate 205 and tautomerism affords the corresponding product 202 (Scheme 62).^93^

**Scheme 63:**
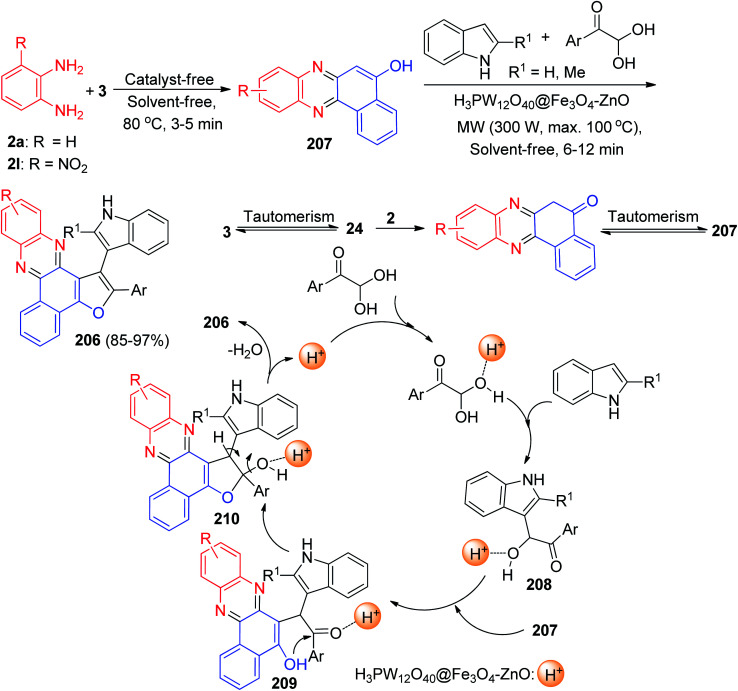
Synthesis of benzo[*a*]furo[2,3-*c*]phenazine derivatives 206.

In 2021, one-pot synthesis of benzo[*a*]furo[2,3-*c*]phenazine derivatives 206 reported in 85–97% yields *via* a multi-component of 3, benzene-1,2-diamines, arylglyoxal and indoles in the presence of H_3_PW_12_O_40_@Fe_3_O_4_–ZnO magnetic core–shell nanoparticles (MCNPs) under solvent-free conditions using microwave irradiation (300 W, max. 100 °C) for 6–12 min. A plausible mechanism is depicted in Scheme 63. Based on this mechanism, at first, 3 tautomerizes to intermediate 24. The primary condensation of 24 with benzene-1,2-diamine obtains benzo[*a*]phenazin-5-ol 207. On the other hand, the reaction of indole with arylglyoxal afforded intermediate 208. After that, intermediate 207 reacts with intermediate 208 to give an intermediate 209. This then forms intermediate 210 through the intramolecular ring closure, followed dehydration leads to the formation of the desired product 206.^94^

**Scheme 64:**
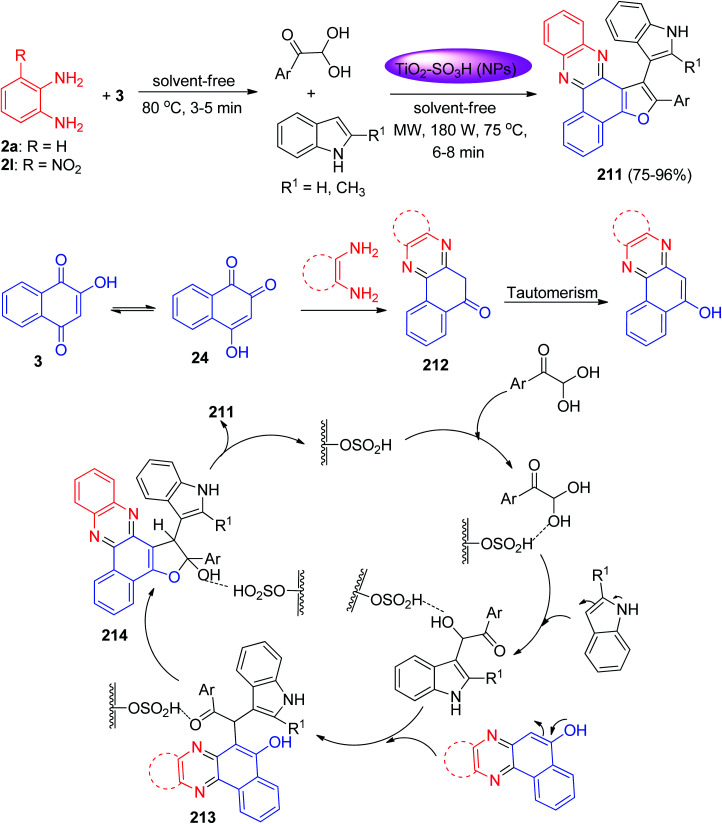
Microwave-assisted synthesis of benzo[*a*]furo[2,3-*c*]phenazine derivatives 211.

Next, Mohebat and co-workers developed an environmentally benign procedure for the synthesis of furo[2,3-*c*]phenazine derivatives 211 in 75–96% yields *via* reactions of 3, 1,2-phenylenediamines, arylglyoxals, and indole in the presence of TiO_2_–SO_3_H-catalyst (TSAC) as a recyclable heterogeneous catalyst under solvent-free conditions using microwave irradiation (180 W, 75 °C) for 6–8 min. A plausible reaction mechanism is shown in Scheme 64. Based on this mechanism, at first, 3 tautomerizes to intermediate 24. The primary condensation of 24 with benzene-1,2-diamine yielded benzo[*a*]phenazin-5-ol 212. A reaction pathway involves the formation of an intermediate 213 medium when reacted with the indole group after being added to the carbonyl group or upon the replacement of the side-chain hydroxy group. Based on the second route, arylglyoxal and indole developed the open shaft 213 or the OH group electron pair. This, then forms intermediate 214 informing the C–C and C–N bonds through the intermolecular [3 + 2] ring reaction, which is made after reacting with the intermediate middle benzo[*a*] phenazine-5-ol, caused by the loss of water during the ring formation process before the desired product is obtained.^95^

### 2.6 Synthesis of benzooxazinophenazines

**Scheme 65:**
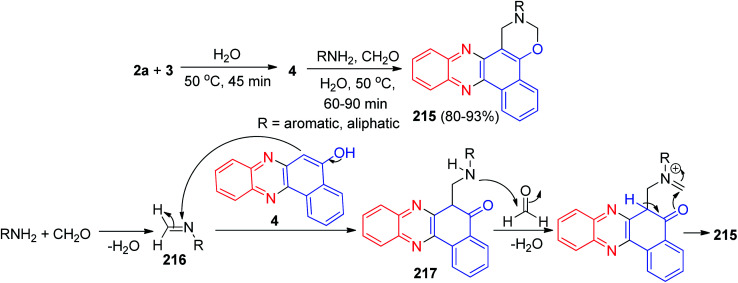
Synthesis of benzo[*a*][1,3]oxazino[6,5-*c*]phenazine derivatives 215.

In 2014, a catalyst-free multi-component reaction capable of affording a wide range of benzo[*a*][1,3]oxazino[6,5-*c*]phenazine derivatives 215 in 80–93% yields has been reported *via* the reaction of 3, 1,2-diaminobenzene, aromatic/aliphatic amines and formaldehyde at 50 °C in water within 60–90 min. It should be noted that he same reaction did not proceed with heterocyclic amines *viz.* 2-aminobenzothiazole, 5-amino-3-methylpyrazole, 1,3-dimethyl-5-aminouracil *etc.* A proposed mechanistic route for the formation of the products is exhibited in Scheme 65. At first, amination reaction occurs between the formaldehyde and amine followed by H_2_O elimination providing imine intermediate 216. 216 is then attacked by 4 to form 217 which further reacts with formaldehyde and eliminates H_2_O, further cyclization occurs to form the final product 215.^96^

**Scheme 66:**
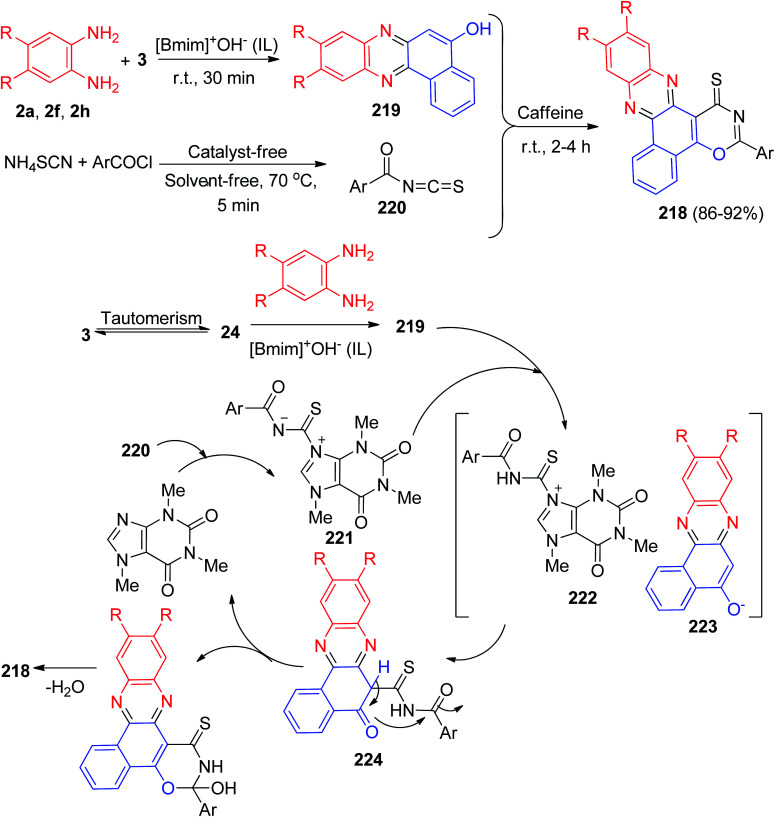
Caffeine catalyzed synthesis of benzo[*a*][1,3]oxazino[6,5-*c*]phenazine derivatives 218.

Next, Mohebat and Yazdani-Elah-Abadi synthesized benzo[*a*][1,3]oxazino[6,5-*c*]phenazine derivatives 218 in 86–92% yields by the one-pot, four-component sequential condensation between 3, aromatic 1,2-diamines, ammonium thiocyanate and acid chlorides in the presence of caffeine as a green and natural catalyst in a basic ionic liquid (1-butyl-3-methylimidazolium hydroxide) at room temperature for 2–4 h. The proposed mechanism is outlined in Scheme 66. Initially, 3 tautomerizes to intermediate 24. The condensation of 24 with benzene-1,2-diamine produces benzo[*a*]phenazin-5-ol 219. Then, the formation of aroyl isothiocyanate 220, followed by formation of the 1 : 1 adduct 221 and its subsequent protonation by benzo[*a*]phenazin-5-ol 219 produces 222. The positively charged ion 222 is attacked by the anion of benzo[*a*]phenazin-5-ol 223. Finally, intermediate 224 undergoes a cyclization reaction and elimination of water to produce 218.^97^

**Scheme 67:**
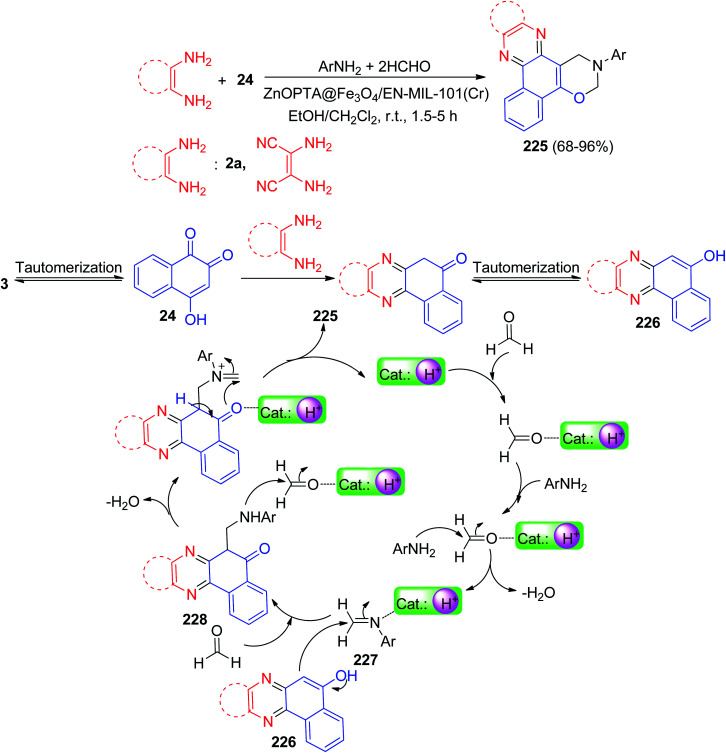
Synthesis 3-phenyl-3,4-dihydro-2*H*-benzo[*a*][1,3] oxazino[5,6-*c*]phenazine derivatives 225.

In 2020, Mohebat and co-workers reported a one-pot procedure for the synthesis of 3-phenyl-3,4-dihydro-2*H*-benzo[*a*][1,3]oxazino[5,6-*c*]phenazine derivatives 225 in 68–96% yields by four-component coupling reaction between benzo[*a*]phenazine-5-ol, formaldehyde and amine in the presence of a catalytic amount ZnOPTA@Fe_3_O_4_/EN-MIL-101(Cr) nanopowder in EtOH–CH_2_Cl_2_ at room temperature under stirring condition within 1.5–5 h. A plausible rational mechanism for the four-part reaction is illustrated in Scheme 67. Based on this mechanism, at first, 3 tautomerizes to intermediate 24. The condensation of 24 with 1,2-diamines produces benzo[*a*]phenazin-5-ol 226. At first, amination reaction occurs between the activated formaldehyde (by coordination with catalyst nanoparticle) and amine followed by H_2_O elimination provides imine intermediate 227 that is further activated by nanocatalyst. Intermediate 227 is then attacked by 226 to form 228 which further reacts with formaldehyde and eliminate H_2_O. Next cyclization occurs to form the final product.^98^

### 2.7 Synthesis of the other benzo[*a*]phenazine-5-ol derivatives

**Scheme 68:**
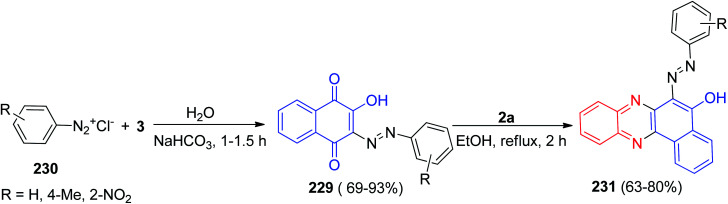
Synthesis of 2-hydroxy-6-arylazobenzo[*a*]phenazines 231.

In 2002, a series of 2-hydroxy-3-arylazo-1,4-naphthoquinones 229 in 69–93% yields were prepared by coupling of 3 with aryldiazonium chlorides 230 in water or aqueous alcohol (1 : 1) in the presence of sodium carbonate or hydrocarbonate for 1–1.5 h. Then, condensation of 229 with 2a in refluxing EtOH for 2 h led to the formation of 2-hydroxy-6-arylazobenzo[*a*]phenazines 231 in 63–80% yields (Scheme 68).^99^

**Scheme 69:**
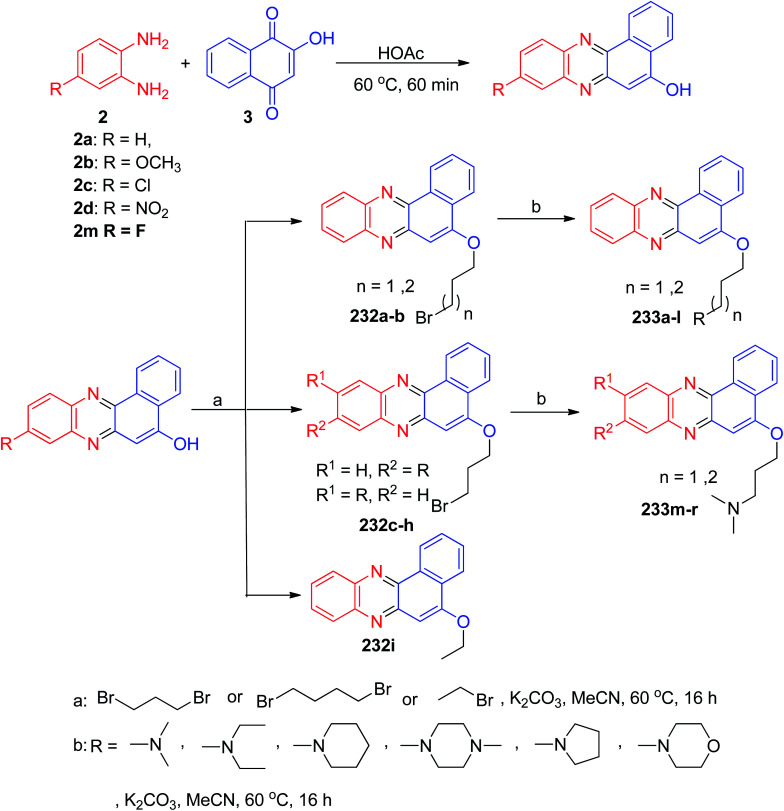
Synthesis of benzo[*a*]-phenazine derivatives 232–233.

In 2013, Huang and co-workers described synthesis of benzo[*a*]phenazine derivatives 232a–i in 50–68% yields *via* one-pot two-step procedure by the reaction of 3, 1,2-phenylene diamines and 1,3-dibromopropane or 1,4-dibromobutane or 2-bromoethane in CH_3_CN with K_2_CO_3_ at 60 °C for 16 h. Subsequent amination of 232 with secondary amines in CH_3_CN at 60 °C with K_2_CO_3_ afforded the desired compounds 233a–r in 55–78% yields after 16 h (Scheme 69). Most of derivatives showed good antiproliferative activity with a range of IC_50_ values of 1–10 μM on the four cancer cell lines HeLa, A549, MCF-7, and HL-60. Topoisomerase-mediated DNA relaxation assay results showed that derivatives could effectively inhibit the activity of both Topo I and Topo II, and the structure–activity relationship studies indicated the importance of introducing an alkylamino side chain.^100^

**Scheme 70:**
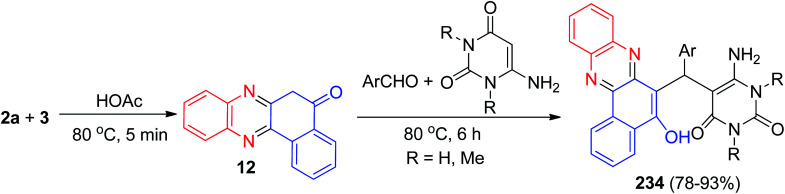
Synthesis of aminouracil-tethered tri-substituted methane derivatives 234.

In 2014, Cai and Lu reported synthesis of aminouracil-tethered tri-substituted methane derivatives 234 in 78–93% yields by the reaction of 3, 2a, aromatic aldehydes and aminouracil derivatives in AcOH at 80 °C for 6 h (Scheme 70).^101^

**Scheme 71:**
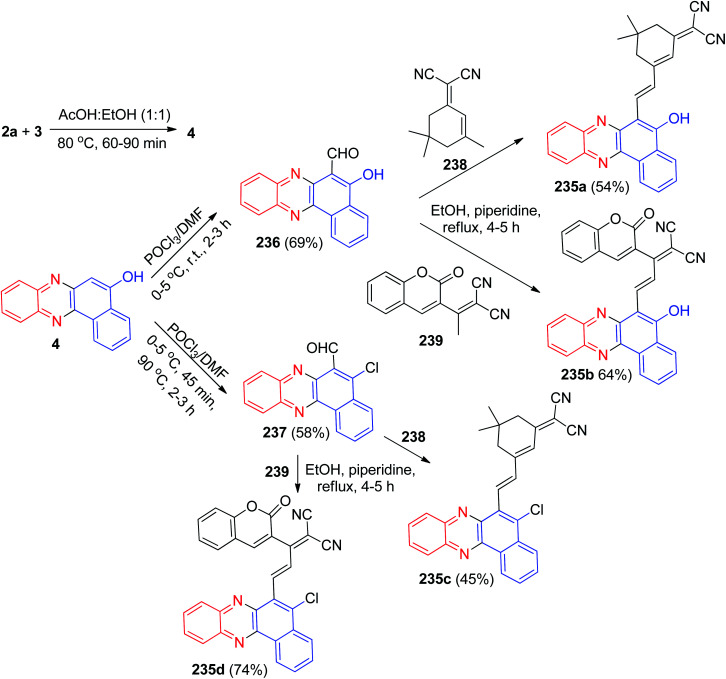
Synthesis and fluorescence of benzofused phenazine π-conjugated skeleton 235.

After that, benzofused phenazine π-conjugated skeleton 235a–d with a coumarin and isophoron core was synthesized by Sekar and co-workers. Benzo[*a*]phenazin-5-ol (4) was prepared by condensation of 3 with 2a in AcOH : EtOH (50 : 50) at 80 °C for 60–90 min. 5-Hydroxybenzo[*a*]phenazine-6-carbaldehyde 236 and 5-chloro[*a*]phenazine-6-carbaldehyde 237 were prepared by the Vilsmeier–Haack reaction. The compounds 236 or 237 on treatment with 238 or 239 active methylene compounds, respectively, in the DMSO/ethanol in the presence of catalytic amount of piperidine atreflux temperature (70–80 °C) for 4–5 h gave (*E*)-2-(3-(5-hydroxybenzo[*a*]phenazin-6-yl)-1-(2-oxo-2*H*-chromen-3-yl)allylidene)malononitrile 235a–b and (*E*)-2-(3-(5-chlorobenzo[*a*]phenazin-6-yl)-1-(2-oxo-2*H*-chromen-3-yl)allylidene) malononitrile 235c–d (Scheme 71).^102^

**Scheme 72:**
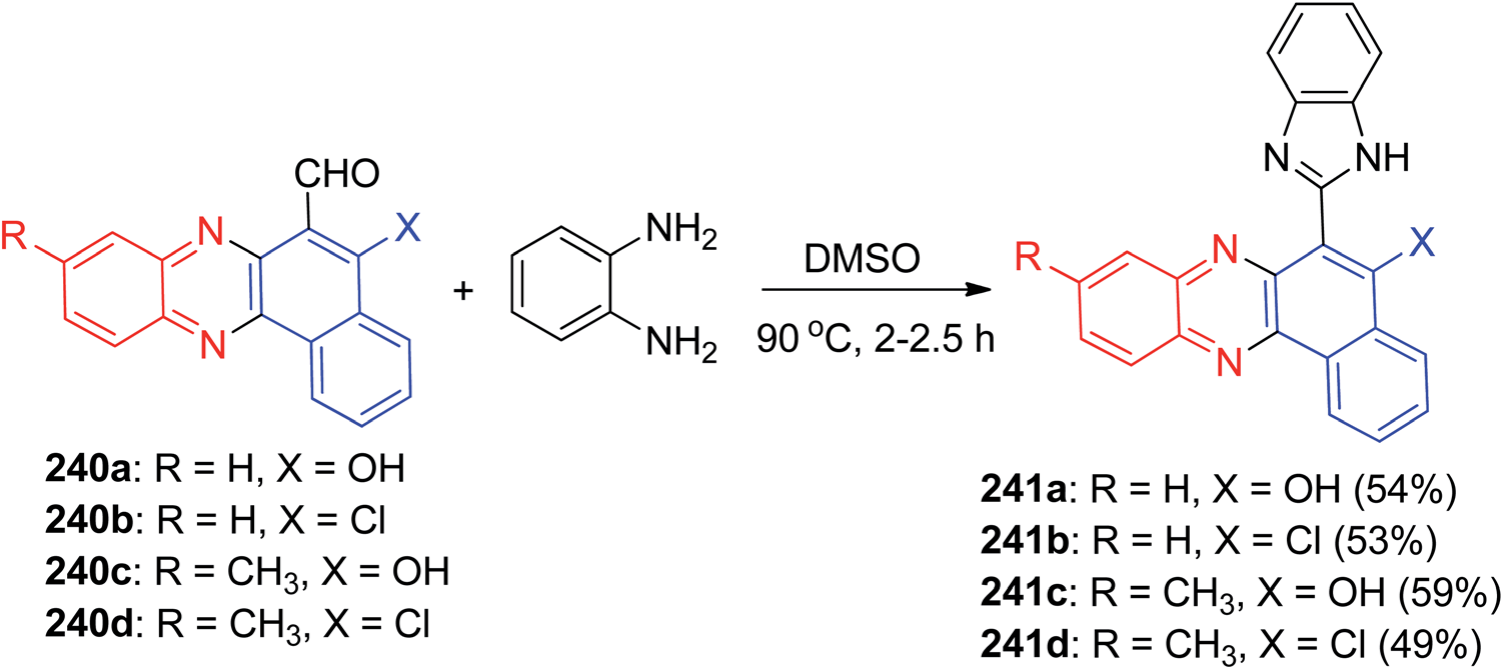
Synthesis of 6-(1*H*-benzo[*d*]imidazol-2-yl) benzo[*a*] phenazin-5-ols 241a–d.

In addition, Sekar and Choudhary prepared 5-hydroxybenzo[*a*]phenazine-6-carbaldehyde 240a–d by the Vilsmeyer Haack reaction. Then, these compounds on treatment with 2a in the presence of DMSO at 90 °C for 2–2.5 h gave 6-(1*H*-benzo[*d*]imidazol-2-yl) benzo[*a*]phenazin-5-ol derivatives 241a–d in 49–59% yields (Scheme 72). Also, the UV-vis absorption and fluorescence emission spectra of these compounds were studied in solvents of differing polarity; the dyes exhibited excited state intramolecular proton transfer.^103^

**Scheme 73:**
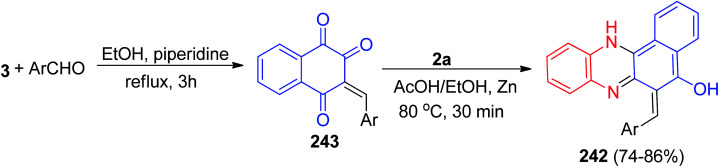
Synthesis of styryl-dihydrobenzo[*a*]phenazine chromophores 242.

Next, Sekar and co-workers have synthesized styryl-dihydrobenzo[*a*]phenazine chromophores 242 in 74–86% yields by a two-step process as outlined in Scheme 73. In the first step, 3 was treated with the corresponding aromatic aldehydes in the presence of a catalytic amount of piperidine and ethanol at reflux temperature for 3 h to yield 3-substituted 1,2,4-triketo naphthoquinone styryl derivatives 243. The substituted naphthoquinones 243 were conveniently converted to dihydrobenzo[*a*]phenazines 242 by refluxing with 2a in AcOH/EtOH at 80 °C and subsequently reduced by Zn. They respond to acids and bases through changes in absorption resulting in strong bathochromic shift (>437 nm). The emission quantum yields of the dyes were in the range 0.11–0.14.^104^

**Scheme 74:**
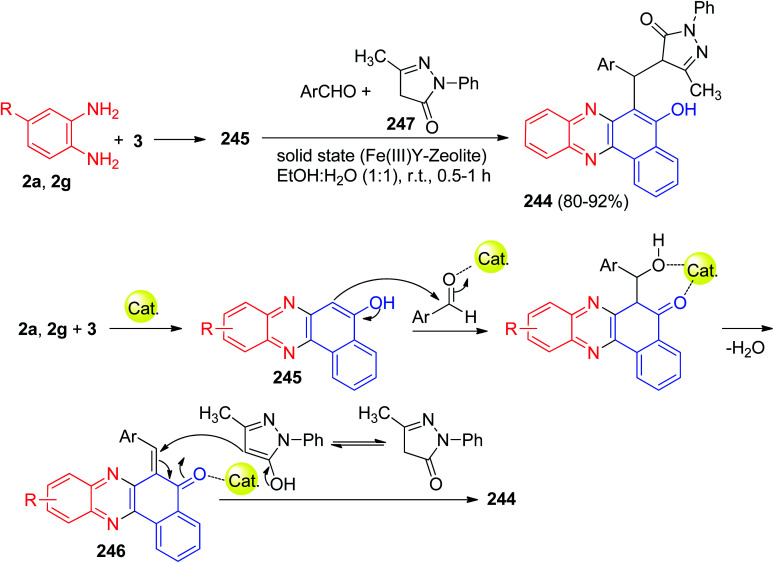
Fe(iii)Y-zeolite catalyzed synthesis of hydroxybenzo[*a*]phenazine pyrazol-5(4*H*)-one derivatives 244.

Later, Perumal *et al.* described the series of fabrication of nanofibrous scaffold loaded with potential biologically active hydroxybenzo[*a*]phenazine pyrazol-5(4*H*)-one derivatives 244 were designed, synthesized by a simple one-pot, two step four component condensation based on Michael type addition reaction of 3, benzene-1,2-diamines, aromatic aldehydes and 3-methyl-1-phenyl-1*H*-pyrazol-5(4*H*)-one as the substrates. The heterogeneous solid state catalyst (Fe(iii)Y-zeolite) could effectively catalyze the reaction in EtOH : H_2_O (1 : 1) at room temperature for 0.5–1 h to obtain the products with high yields (80–92%). Furthermore, the synthesized derivatives showed anticancer and antimicrobial activities. The formation of the products is expected to proceed through two steps. Initially there occurs condensation between 3 with 2 to give the intermediate benzo[*a*]phenazin-5-ol 245, the second step is an aldol-type condensation that takes place between aromatic aldehyde with 245, resulting *in situ* C=C bond formation of intermediate 246, which on further reaction undergoes Michael-type addition with another molecule of 3-methyl-1-phenyl-1*H*-pyrazol-5(4*H*)-one 247 keto–enol tautomerism under the influence of the Fe(iii)Y-zeolite solid state catalyst to furnish the desired product 244(Scheme 74).^105^

**Scheme 75:**
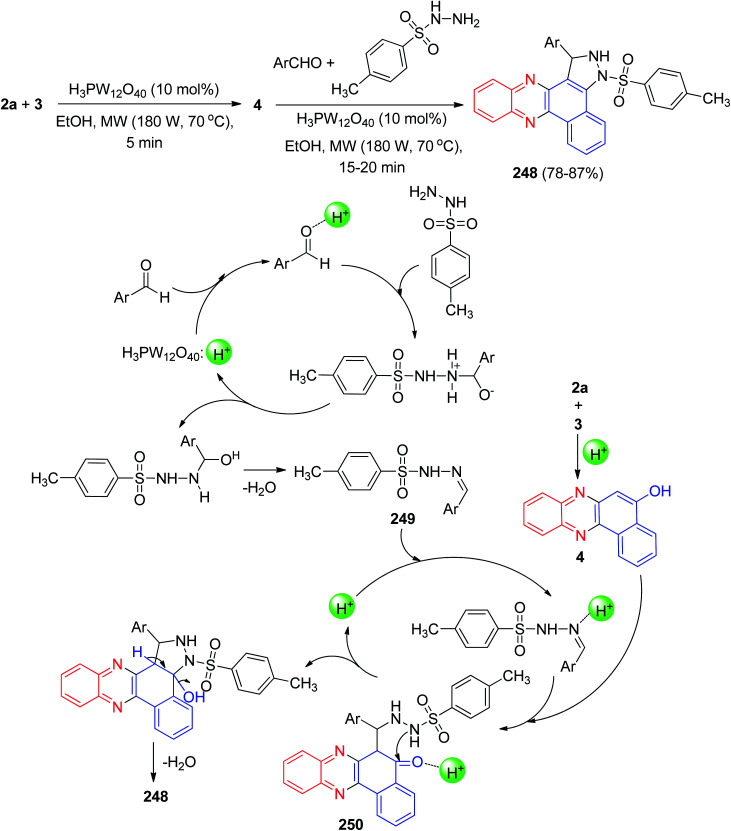
H_3_PW_12_O_40_ catalyzed synthesis of 11*H*-benzo[*a*]pyrazolo[3,4-*c*]phenazine derivatives 248.

In 2018, for the synthesis of pyrazolo-fused benzophenazines 248 in 78–87% yields, Mohebat and co-workers developed a multi-component condensation reaction involving 3, 2a, aromatic aldehydes, and 4-methylbenzenesulfonohydrazide in the presence of phosphotungstic acid (H_3_PW_12_O_40_) under microwave irradiation (180 W, max. 70 °C) in EtOH within 15–20 min. A detailed reaction mechanism is shown in Scheme 75. In this mechanism, the primary condensation of 3 with 2a gives 4. On the other hand, H_3_PW_12_O_40_ is an efficient catalyst to form the hydrazone 249, which readily forms *in situ* from the condensation of the aromatic aldehyde with 4-methylbenzenesulfonohydrazide. The Michael addition of 4 with 249 in the presence of the catalyst finally gives the intermediate 250, which then forms the inner molecular ring after a tautomeric proton shift to produce the corresponding product 248.^106^

**Scheme 76:**
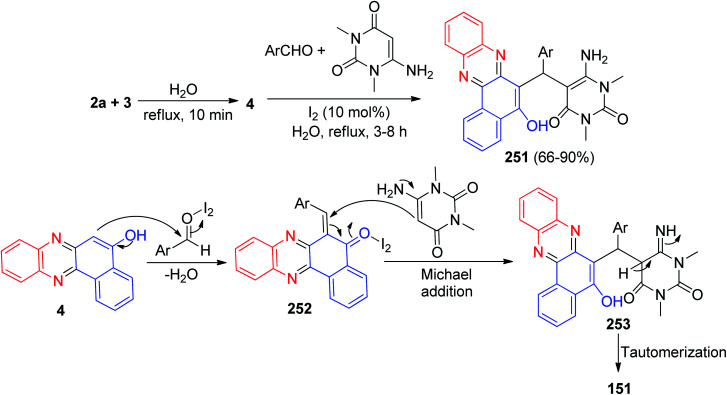
I_2_ catalyzed synthesis of aminouracil-tethered tri-substituted methane derivatives 251.

In 2019, Further, Parvin *et al.* developed a one-pot four-component reaction involving 3, 2a, aromatic aldehydes and aminouracil derivatives. The reaction was catalyzed by molecular iodine in water under reflux conditions for 3–8 h affording in aminouracil-tethered tri-substituted methane derivatives 251 in 66–90% yields. The proposed mechanism for the synthesis of 251 has been presented in Scheme 76. Firstly, iodine activates the carbonyl group of aldehyde as it acts as a mild Lewis acid by forming aldehyde–iodine complex and increases the electrophilicity of carbonyl carbon. The aldol condensation of aldehyde and 5-hydroxybenzophenazine (formed from the reaction of 3 and 2a) followed by dehydration resulted in 252. Then, molecular iodine also activates carbonyl group of 252 and facilitates the Michael addition with 1,3-dimethyl-6-aminouracil and provided 253. Next, tautomerization of 253 resulted in the final product 251.^107^

**Scheme 77:**
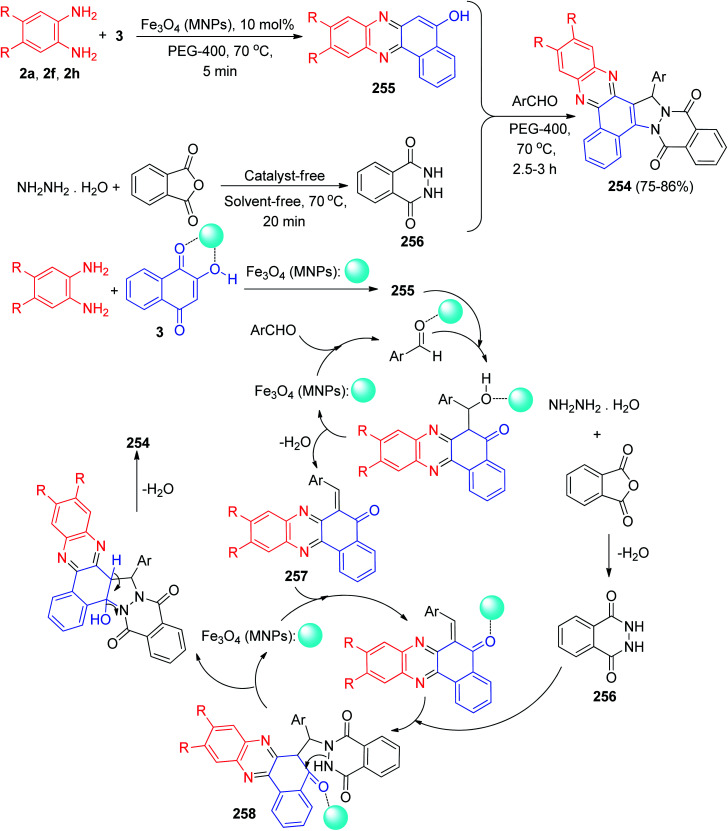
Fe_3_O_4_ MNPs catalyzed synthesis of benzo[*a*]phthalazino[2,3:1,2]pyrazolo[3,4-*c*]phenazines 254.

In 2020, benzo[*a*]phthalazino[2,3:1,2]pyrazolo[3,4-*c*]phenazines 254 are synthesized in 75–86% yields by using a single-pot, five-component reaction involving 3, aromatic 1,2-diamines, hydrazine hydrate, phthalic anhydride, and benzaldehydes catalyzed by magnetic iron(iii) oxide nanoparticles (Fe_3_O_4_ MNPs) in polyethylene glycol (PEG-400) as an inexpensive, non-toxic, and effective medium at 70 °C for 2.5–3 h. A detailed reaction mechanism is outlined in Scheme 77. The primary condensation of 3 with benzene-1,2-diamine in the presence of Fe_3_O_4_-MNPs gives benzo[*a*]phenazin-5-ol 255. Then, hydrazine hydrate condenses with phthalic anhydride to generate the phthalhydrazide 256 with the loss of water. On this mechanism, Fe_3_O_4_-MNPs is an efficient catalyst to form the olefin 257, which readily prepares *in situ* from Knoevenagel condensation of aromatic aldehyde with 255. The Michael addition of phthalhydrazide 256 with olefin 257 in the presence of the catalyst finally gives intermediate 258, which then makes the inner molecular ring to be formed after a tautomeric proton shift to produce the corresponding product 254.^108^

**Scheme 78:**
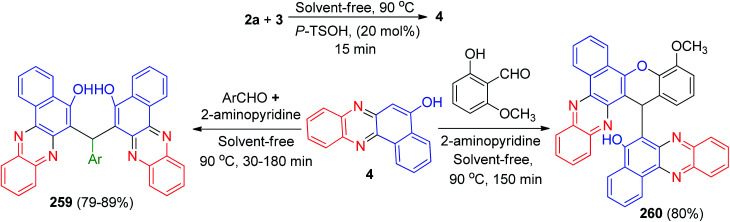
Synthesis of 6,6′-(arylmethylene)bis(benzo[*a*]phenazin-5-ol) derivatives 259.

**Scheme 79:**
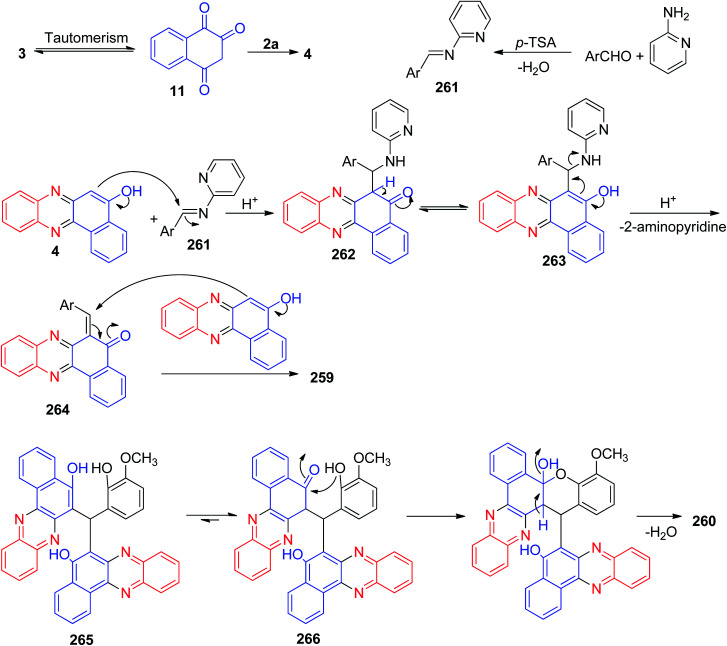
Proposed mechanism for the synthesis of 6,6'-(arylmethylene)bis(benzo[*a*]phenazin-5-ol) derivatives 252 and compound 260.

In 2021, Olyaei and his group synthesized 6,6′-(arylmethylene)bis(benzo[*a*]phenazin-5-ol) derivatives 259 in 79–89% yields *via* a sequential one-pot, two-step, pseudo-five-component tandem reaction starting from 3, 2a and aromatic aldehydes in the presence of 2-aminopyridine as co-catalyst and *p*-TsOH (20 mol%) as catalyst at 90 °C under solvent-free conditions within 30–180 min. Moreover, 6-(4-methoxy-16*H*-benzo[*a*]chromeno[2,3-*c*]phenazin-16-yl)benzo[*a*]phenazin-5-ol (260) prepared by the reaction of 3, 2a and 2-hydroxy-3-methoxybenzaldehyde in the same reaction condition after 150 min (Scheme 78). A reaction mechanism consistent with the above results is shown in Scheme 79. Initially, 3 tautomerizes to intermediate 11. The primary condensation of intermediate 11 with 2a affords 4. On the other hand, condensation of aromatic aldehyde with 2-aminopyridine in the presence of *p*-TsOH afforded Schiff base 261 as intermediate. Subsequently, nucleophilic addition of 4 to intermediate 261 led to the formation of intermediate 262. Intermediate 262 tautomerizes to intermediate 263. By leaving of 2-aminopyridine from intermediate 263, *ortho*-quinonemethide 264 was produced. It should be noted that, 2-aminopyridine might act as a good leaving group in the acidic environment. Finally, Michael addition of 4 to *o*-QM 264 afforded the corresponding product 259.

For the formation of compound 260, initially, intermediate 265 was formed according to the same proposed mechanism for the preparation of 259. Then, intermediate 265 tautomerizes to the keto form 266, which undergoes intramolecular cyclization *via* an oxygen atom attacking to the carbonyl group and elimination of water to afford the desired product 260.^109^

**Scheme 80:**
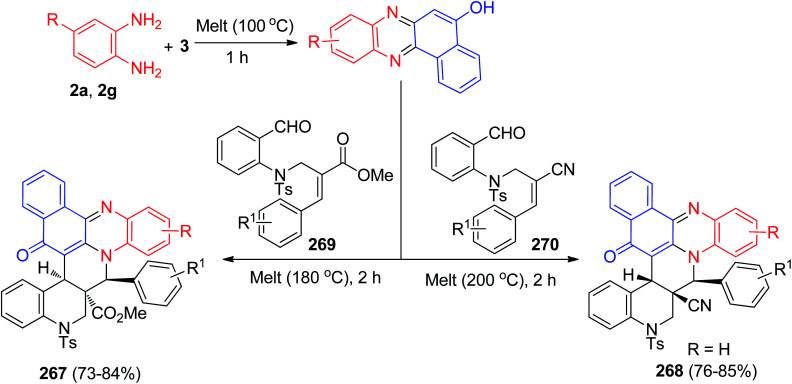
Synthesis of hybrid polycyclic quinolinobenzo[*a*]phenazinones 267–268.

Next, an efficient and versatile protocol for the synthesis of hybrid polycyclic quinolinobenzo[*a*]phenazinones 267 (73–84% yields) and 268 (76–85% yields) has been developed by the reaction of 3, benzene-1,2-diamines and *N*-allylated 2-aminoarylaldehyde derivatives 269 and 270 under solid-state melt reaction (SSMR) condition at 180–200 °C for 2 h (Scheme 80). The reaction was carried out *via* intramolecular domino Knoevenagel-hetero-Diels–Alder reaction involving the generation of six membered fused rings and three contiguous stereogenic centers.^110^

**Scheme 81:**
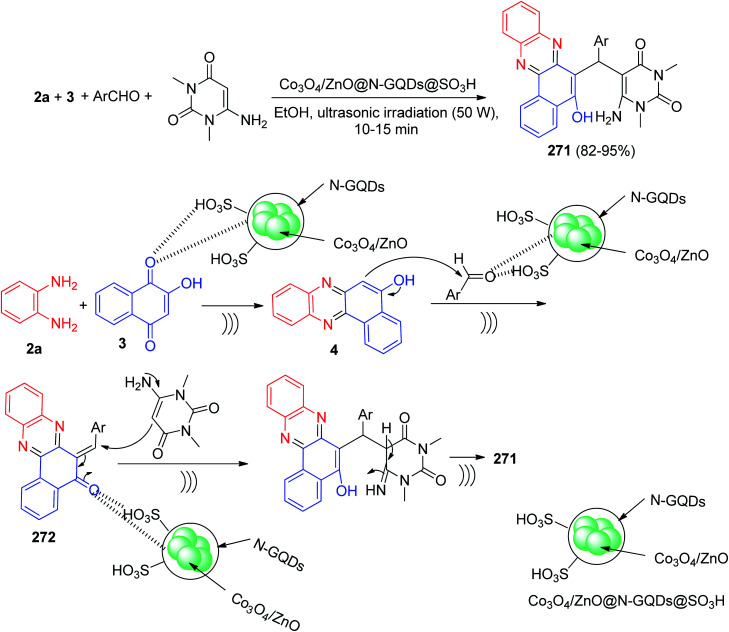
Synthesis of phenazinpyrimidines 271.

Recently, Safaei-Ghomi and his group synthesized phenazinpyrimidines 271 in 82–95% yields *via* one-pot four-component reaction of 3, 2a, aldehydes and 6-amino-1-3-dimethyluracil using Co_3_O_4_/ZnO@N-GQDs@SO_3_H nanocomposite as a robust heterogeneous catalyst under ultrasonic irradiations in EtOH for 10–15 min. Scheme 81 shows a plausible mechanism for this reaction in the presence of nanocatalyst. First, the formation of 4 can be explained *via* a condensation of 3 and 2a. Then the efficient Knoevenagel condensation of 4 and arylaldehyde created intermediate 272. Finally, the product 271 was formed by Michael addition/dehydration reactions between 6-amino-1,3-dimethyluracil and intermediate 272.^111^

## 3 Conclusions

Phenazine and its derivatives such as benzophenazins are a large group of natural and synthesized N-containing heterocycles. Benzophenazins have attracted interest because they exhibit a wide range of biological activities. In this article review, we focused on the important methods for synthesis of lawsone-based benzo[*a*]phenazin-5-ol derivatives and reported the different important reactions of them in synthesis of five and six membered fused heterocycles and the other derivatives. Moreover, the present work contributes the different classical methods with green approach, homogeneous and heterogeneous-catalyzed reactions, microwave irradiation and ultrasound-mediated reactions for the synthesis of benzophenazine derivatives. Thus, this review article will help not only to the synthetic chemists but also to the medicinal and pharmaceutical chemists to update information on recent developments in this field.

## Conflicts of interest

There are no conflicts to declare.

## Supplementary Material

## References

[cit1] Turner J. M., Messenger A. J. (1986). Adv. Microb. Physiol..

[cit2] Kerr J. R. (2000). Infect. Dis. Rev..

[cit3] Laursen J. B., Nielsen J. (2004). Chem. Rev..

[cit4] Guttenberger N., Blankenfeldt W., Breinbauer R. (2017). Bioorg. Med. Chem..

[cit5] Makgatho M. E., Anderson R., O'Sullivan J. F., Egan T. J., Freese J. A., Cornelius N., Van Rensburg C. E. J. (2000). Drug Dev. Res..

[cit6] de Andrade-Neto V. F., Goulart M. O. F., da Silva Filho J. F., da Silva M. J., Maria do Carmo F. R., Pinto A. V., Zalis M. G., Carvalho L. H., Krettli A. U. (2004). Bioorg. Med. Chem. Lett..

[cit7] Neves-Pinto C., Malta V. R. S., Pinto M. D. C. F. R., Santos R. H. A., de Castro S. L., Pinto A. V. (2002). J. Med. Chem..

[cit8] Wang W., Preville P., Morin N., Mounir S., Cai W., Siddiqui M. A. (2000). Bioorg. Med. Chem. Lett..

[cit9] Rewcastle G. W., Denny W. A., Baguley B. C. (1987). J. Med. Chem..

[cit10] Wang S., Miller W., Milton J., Vicker N., Stewart A., Charlton P., Mistry P., Hardick D., Denny W. A. (2002). Bioorg. Med. Chem. Lett..

[cit11] Ye L., Zhang H., Xu H., Zou Q., Cheng C., Dong D., Xu Y., Li R. (2010). Bioorg. Med. Chem. Lett..

[cit12] Wang J., Zhi X., Yu X., Xu H., Agric J. (2013). Food Chem..

[cit13] Muller M., Sorrell T. C. (1995). Prostaglandins.

[cit14] Sivakumar R., Naveenraj S., Anandan S. (2011). J. Lumin..

[cit15] Giddens S. R., Bean D. C. (2007). Int. J. Antimicrob. Agents.

[cit16] De Logu A., Palchykovska L. H., Kostina V. H., Sanna A., Meleddu R., Chisu L., Alexeeva I. V., Shved A. D. (2009). Int. J. Antimicrob. Agents.

[cit17] Shaikh A. M., Sharma B. K., Chacko S., Kamble R. M. (2017). New J. Chem..

[cit18] Wang C. , Mitchell W., D'Lavari M. and Tierney S., PCT Int. Appl., WO 2012123058 A1 20120920, 2012

[cit19] Fischer B. B., Krieger-Liszkay A., Eggen R. I. L. (2004). Environ. Sci. Technol..

[cit20] Katsuhei Y. , Kazuhiko S., Haruka E. and Kazutoshi H., Jpn. Kokai Tokkyo Koho, JP 2010088420 A 20100422, 2010

[cit21] Zhang G., Bala H., Cheng Y., Shi D., Lv X., Yu Q., Wang P. (2009). Chem. Commun..

[cit22] Pauliukaite R., Ghica M. E., Barsan M. M., Brett C. M. A. (2010). Anal. Lett..

[cit23] Liu H., Ying T., Sun K., Li H., Qi D. (1997). Anal. Chim. Acta.

[cit24] Liu H., Zhanen Z., Xiaolin Z., Deyao Q., Liu Y., Yu T., Deng J. (1997). Electrochim. Acta.

[cit25] Chaudhary A., Khurana J. M. (2018). Res. Chem. Intermed..

[cit26] Elhady H. A., El-Mekawy R. E., Fadda A. A. (2020). Polycyclic Aromat. Compd..

[cit27] Vickr N., Burgess L., Chuckowree I. S., Dodd R., Folkes A. J., Hardick D. J., Hancox T. C., Miller W. H., Milton J., Sohal S., Wang S., Wren S. P., Charlton P. A., Dangerefield W., Liddle C., Mistry P., Stewart A. J., Denny W. A. (2002). J. Med. Chem..

[cit28] Hamama W. S., Hassanien A. E.-D. E., Zoorob H. H. (2017). J. Heterocycl. Chem..

[cit29] Jordão A. K., Vargas M. D., Pinto A. C., da Silva F. de C., Ferreira V. F. (2015). RSC Adv..

[cit30] Olyaei A., Sadeghpour M., Khalaj M. (2020). RSC Adv..

[cit31] Sadeghpour M., Olyaei A., Adl A. (2021). New J. Chem..

[cit32] Rehberg G. M., Rutherford J. L. (1995). J. Heterocycl. Chem..

[cit33] Kaupp G., Naimi-Jamal M. R. (2002). Eur. J. Org. Chem..

[cit34] Jain R., Agarwal O. P., Jain S. C. (2013). Asian J. Chem..

[cit35] Choudhary A. S., Malik M. K., Patil S. R., Prabhu K. H., Deshmukh R. R., Sekar N. (2014). Can. Chem. Trans..

[cit36] Wang S.-L., Wu F.-Y., Cheng C., Zhang G., Liu Y.-P., Jiang B., Shi F., Tu S.-J. (2011). ACS Comb. Sci..

[cit37] Shaterian H. R., Moradi F., Mohammadnia M. (2012). C. R. Chim..

[cit38] Shaterian H. R., Mohammadnia M. (2013). J. Mol. Liq..

[cit39] Hasaninejad A., Firoozi S. (2013). Mol. Diversity.

[cit40] Yazdani Elah Abadi A., Maghsoodlou M.-T., Heydari R., Mohebat R. (2016). Res. Chem. Intermed..

[cit41] Esmaeilpour M., Sardarian A. R., Firouzabadi H. (2018). ChemistrySelect.

[cit42] Abolghassem S., Molaei S., Javanshir S. (2019). Heliyon.

[cit43] Naeimi H., Farahnak Zarabi M. (2019). RSC Adv..

[cit44] Ghorbani-Choghamarani A., Mohammadi M., Shiri L., Taherinia Z. (2019). Res. Chem. Intermed..

[cit45] Nikoorazm M., Khanmoradi M., Mohammadi M. (2020). Appl. Organomet. Chem..

[cit46] Nikoorazm M., Khanmoradi M. (2020). Catal. Lett..

[cit47] Mishra A., Pandey Y. K., Tufail F., Singh J., Singh J. (2020). Catal. Lett..

[cit48] Daraie M., Tamoradi T., Heravi M. M., Karmakar B. (2021). J. Mol. Struct..

[cit49] Farahnak Zarabi M., Naeimi H. (2021). Polycyclic Aromat. Compd..

[cit50] Taheri S., Mollabagher H., Seyed Mousavi S. A. H. (2021). Polycyclic Aromat. Compd..

[cit51] Mohebat R., Yazdani Elah Abadi A., Maghsoodlou M.-T. (2016). Res. Chem. Intermed..

[cit52] Khurana J. M., Chaudhary A., Lumb A., Nand B. (2012). Green Chem..

[cit53] Reddy M. V., Valasani K. R., Lim K. T., Jeong Y. T. (2015). New J. Chem..

[cit54] Rajeswari M., Khanna G., Chaudhary A., Khurana J. M. (2015). Synth. Commun..

[cit55] Yazdani-Elah-Abadi A., Mohebat R., Maghsoodlou M.-T., Heydari R. (2018). Polycyclic Aromat. Compd..

[cit56] Harichandran G., Parameswari P., Shanmugam P. (2018). Sens. Actuators, B.

[cit57] Nazeef M., Saquib M., Tiwari S. K., Yadav V., Ansari S., Sagir H., Hussain M. K., Siddiqui I. R. (2020). ChemistrySelect.

[cit58] Mohebat R., Yazdani Elah Abadi A., Maghsoodlou M.-T., Mohammadi M., Heydari R. (2016). Res. Chem. Intermed..

[cit59] Yazdani-Elah-Abadi A., Mohebat R., Kangani M. (2016). J. Chem. Res..

[cit60] Mohammadrezaei M., Mohebat R., Tabatabaee M. (2018). J. Chin. Chem. Soc..

[cit61] Yazdani-Elah-Abadi A., Lashkari M., Mohebat R. (2020). Org. Prep. Proced. Int..

[cit62] Taheri M., Mohebat R. (2020). Green Chem. Lett. Rev..

[cit63] Taheri M., Mohebat R., Moslemin M. H. (2021). Polycyclic Aromat. Compd..

[cit64] Mohammadrezaei M., Mohebat R., Tabatabaee M. (2019). Org. Prep. Proced. Int..

[cit65] Amanpour T., Mirzaei P., Bazgir A. (2012). Synthesis.

[cit66] Saluja P., Chaudhary A., Khurana J. M. (2014). Tetrahedron Lett..

[cit67] Shaabani A., Ghadari R., Arabieh M. (2014). Helv. Chim. Acta.

[cit68] Mohebat R., Yazdani-Elah-Abadi A., Maghsoodlou M.-T., Hazeri N. (2017). Chin. Chem. Lett..

[cit69] Tukhvatshin R. S., Kucherenko A. S., Nelyubina Y. V., Zlotin S. G. (2019). J. Org. Chem..

[cit70] Nagaraju P., Reddy P. N., Padmaja P., Ugale V. G. (2020). Chem. Data Collect..

[cit71] Choudhary A. S., Sekar N. (2015). J. Fluoresc..

[cit72] Mayakrishnan S., Arun Y., Balachandran C., Awale S., Maheswari N. U., Perumal P. T. (2017). ACS Omega.

[cit73] Kostenko A. A., Bykova K. A., Kucherenko A. S., Komogortsev A. N., Lichitsky B. V., Zlotin S. G. (2021). Org. Biomol. Chem..

[cit74] Mahdavinia G. H., Mirzazadeh M., Notash B. (2013). Tetrahedron Lett..

[cit75] Lu Y., Wang L., Wang X., Xi T., Liao J., Wang Z., Jiang F. (2017). Eur. J. Med. Chem..

[cit76] Hasaninejad A., Firoozi S., Mandegani F. (2013). Tetrahedron Lett..

[cit77] Bharti R., Parvin T. (2016). Mol. Diversity.

[cit78] Yazdani-Elah-Abadi A., Maghsoodlou M.-T., Mohebat R., Heydari R. (2017). Chin. Chem. Lett..

[cit79] Yazdani-Elah-Abadi A., Mohebat R., Kangani M. (2017). J. Chin. Chem. Soc..

[cit80] Safaei-Ghomi J., Bakhtiari A. (2019). Appl. Organomet. Chem..

[cit81] Verma K., Tailor Y. K., Khandelwal S., Agarwal M., Rushell E., Kumari Y., Awasthi K., Kumar M. (2018). RSC Adv..

[cit82] Mohebat R., Simin N., Yazdani-Elah-Abadi A. (2019). Polycyclic Aromat. Compd..

[cit83] Mohebat R., Yazdani Elah Abadi A., Maghsoodlou M.-T., Mohammadi M. (2016). Res. Chem. Intermed..

[cit84] Abbasi Pour S., Yazdani-Elah-Abadi A., Afradi M. (2017). Appl. Organomet. Chem..

[cit85] Mirmiran-Yazdi S.-A., Yazdani-Elah-Abadi A., Shams N., Mohebat R. (2017). Turk. J. Chem..

[cit86] Yazdani-Elah-Abadi A., Abbasi Pour S., Kangani M., Mohebat R. (2017). Monatsh. Chem..

[cit87] Tabibian M., Mohebat R., Tabatabaee M. (2018). Turk. J. Chem..

[cit88] Dehghan P., Mohebat R. (2020). Polycyclic Aromat. Compd..

[cit89] Dehghan P., Mohebat R. (2020). Polycyclic Aromat. Compd..

[cit90] Aggarwal K., Khurana J. M. (2015). J. Photochem. Photobiol., A.

[cit91] Aggarwal K., Khurana J. M. (2015). J. Lumin..

[cit92] Yazdani-Elah-Abadi A., Mohebat R., Maghsoodlou M.-T. (2016). RSC Adv..

[cit93] Zarei Haji Abadi M., Mohebat R., Mosslemin M. H. (2020). Polycyclic Aromat. Compd..

[cit94] Taheri M., Mohebat R., Moslemin M. H. (2021). Artif. Cells, Nanomed., Biotechnol..

[cit95] Taheri M., Mohebat R., Moslemin M. H. (2021). Curr. Org. Synth..

[cit96] Khanna G., Chaudhary A., Khurana J. M. (2014). Tetrahedron Lett..

[cit97] Mohebat R., Yazdani-Elah-Abadi A. (2017). Chin. Chem. Lett..

[cit98] Taheri M., Mohebat R., Moslemin M. H. (2020). Green Chem. Lett. Rev..

[cit99] Romanyuk A. L., Polishchuk O. P., Litvin B. L., Ganushchak N. I. (2002). Russ. J. Gen. Chem..

[cit100] Zhuo S.-T., Li C.-Y., Hu M.-H., Chen S.-B., Yao P.-F., Huang S.-L., Ou T.-M., Tan J.-H., An L.-K., Li D., Guand L.-Q., Huang Z.-S. (2013). Org. Biomol. Chem..

[cit101] Lu G.-p., Cai C. (2014). J. Heterocycl. Chem..

[cit102] Choudhary A. S., Patil S. R., Sekar N. (2015). J. Fluoresc..

[cit103] Choudhary A. S., Sekar N. (2015). J. Fluoresc..

[cit104] Patil S. R., Choudhary A. S., Sekar N. (2016). Tetrahedron.

[cit105] Kandhasamy S., Ramanathan G., Muthukumar T., Thyagarajan S., Umamaheshwari N., Santhanakrishnan V. P., Sivagnanam U. T., Perumal P. T. (2017). Mater. Sci. Eng., C.

[cit106] Mohebat R., Dehgan P., Yazdani-Elah-Abadi A. (2018). J. Chin. Chem. Soc..

[cit107] Kumari P., Bharti R., Parvin T. (2019). Mol. Diversity.

[cit108] Yazdani-Elah-Abadi A., Mohebatb R., Lashkari M. (2020). Polycyclic Aromat. Compd..

[cit109] Olyaei A., Aghajanzadeh A., Feizy E., Sadeghpour M. (2021). J. Chin. Chem. Soc..

[cit110] Bakthadoss M., Vinayagam V. (2021). Mol. Diversity.

[cit111] Safaei-Ghomi J., Pooramiri P., Babaei P. (2021). J. Chin. Chem. Soc..

